# Pivotal Role of Surface Terminations in MXene Thermodynamic Stability

**DOI:** 10.1021/acs.chemmater.4c02274

**Published:** 2024-10-11

**Authors:** Ervin Rems, Yong-Jie Hu, Yury Gogotsi, Robert Dominko

**Affiliations:** † National Institute of Chemistry, Ljubljana 1001, Slovenia; ‡ Faculty of Chemistry and Chemical Technology, University of Ljubljana, Ljubljana 1000, Slovenia; § Department of Materials Science and Engineering, Drexel University, Philadelphia, Pennsylvania 19104, United States; ∥ A.J. Drexel Nanomaterials Institute, Drexel University, Philadelphia, Pennsylvania 19104, United States; ⊥ ALISTORE - European Research Institute, CNRS FR 3104, Amiens, Cedex 80039, France

## Abstract

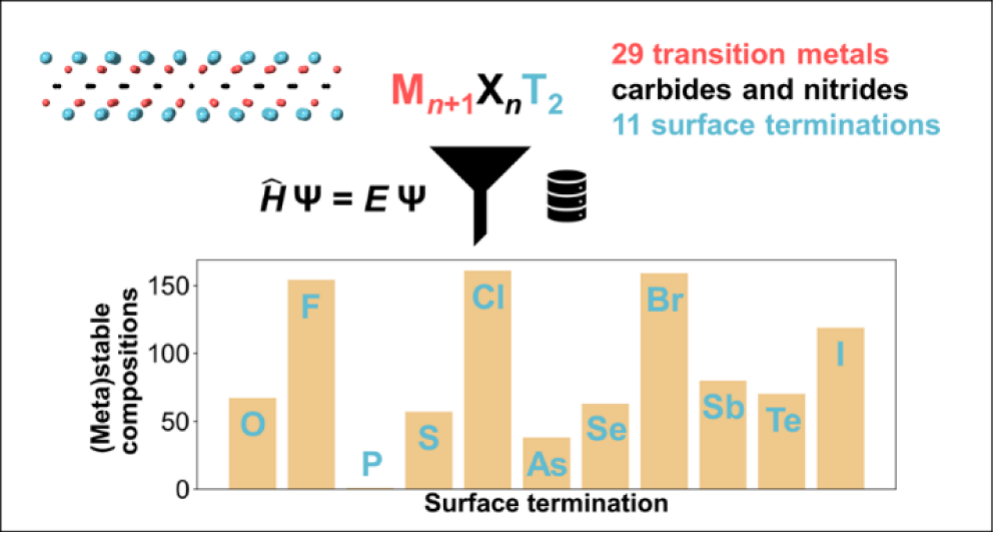

MXenes, i.e., two-dimensional transition metal carbides and nitrides, have been reported as promising materials for various applications, including energy storage, biomedicine, and electronics. The family of MXenes has proliferated, and the chemical space of synthesized MXenes has expanded to 13 transition metals and a dozen elements in surface terminations. The diverse chemistry of MXenes enables systematical tuning of MXene properties to meet the needs of target applications. However, synthesizing new MXene compositions largely relies on a trial-and-error approach. To overcome it, computational predictions of MXene compositions that are thermodynamically stable are crucial to rationalize experimental efforts. Here, we report a comprehensive computational screening for thermodynamically stable MXenes across 29 transition metals and 11 surface terminations. Density functional theory calculations are employed to compute the energy above the convex energy hull as a descriptor of thermodynamic stability. The results are analyzed to explore factors crucial for determining the thermodynamic stability of MXenes, by which the chemistry of surface terminations is found to play a crucial role. The insights on the chemistry of 998 MXene compositions predicted to be (meta)stable are given to systematically guide further research on MXene synthesis and application.

## 1 Introduction

MXenes, functional-group terminated two-dimensional (2D) transition metal carbides or nitrides, are a large and chemically diverse family of materials. Their versatile properties make them ideal for a wide range of applications, including energy generation and storage, catalysis, healthcare, photonics, and electronics.^1^

MXenes have a layered hexagonal structure with a composition M*_n_*_+1_X*_n_*T*_x_*, where M is a transition metal, X is C or N, T is a surface termination group, and *n* is an integer up to 4. The chemistry of M, X, and T sites plays a decisive role in determining the properties and applications of MXenes.^2^ For example, the surface terminations (T) impact the electrochemical and catalytic behavior of MXene. The Ti_3_C_2_T*_x_* (T = O, OH, F) MXene is a pseudocapacitive material that can be utilized in the electrodes of electrochemical supercapacitors,^3^ while Ti_3_C_2_T’*_x_* (T’ = Br, I) MXene exhibits faradaic electrochemical behavior with a flat voltage discharge plateau required for application as battery electrode material.^4^ By tuning the chemistry of transition metal (M), one can affect the catalytic activity of MXene. Mo_2_CT*_x_* exhibits significantly higher electrocatalytic activity for the hydrogen evolution reaction (HER) than Ti_2_CT*_x_*^5^. For instance, the interaction of MXene with electromagnetic waves can be affected by the chemistry of carbide/nitride layers (X). Ti_3_CNT*_x_* provides an exceptional electromagnetic interference (EMI) shielding performance, exceeding that of Ti_3_C_2_T*_x_* MXene.^6^

These examples illustrate the high tunability of MXene properties by exploring and exploiting the wide chemical space of its constituents. However, the whole range of possible MXene compositions is impossible to fully explore experimentally. A possible approach to speed up the discovery of new MXene compositions through self-driven laboratories (SDLs) is not yet fully functional.^7^ To achieve the best performance, SDLs should rely on a synergic combination of physics-based models and data-driven approaches. Specifically, *in silico* screening based on the density functional theory (DFT) calculations is valuable for predictive identification of (meta)stable target materials and some of their physical and chemical properties.

Indeed, many studies employ DFT to systematically explore the chemical space of MXenes. Various MXene compositions were screened to identify the most dynamically^8^ and thermodynamically^9^ stable MXenes. The feasibility of MXene synthesis through selective etching layered ceramic MAX-phase precursors with different compositions was also comprehensively investigated.^10,11^ DFT-screening studies were also performed to identify MXenes with application-dependent target properties, e.g., high-capacity battery electrode materials,^12,13^ catalysts with outstanding HER activity,^14^ CO_2_ capture ability,^15^ ferroelectric MXenes,^16^ and carbon monoxide sensors.^17^

DFT screening studies have successfully guided experimentalists in synthesizing new MXenes^18^ and selecting MXene compositions that yield the desired properties.^5,19^ The surge in interest to explore MXenes beyond the Ti_3_C_2_T*_x_* (T = O, OH, F) composition primarily centered around variations in the transition metal site (M), while the chemical diversity of surface termination atoms (T) received much less attention. The synthesis of MXenes through molten salt etching,^20,21^ chemical vapor deposition (CVD),^22^ or dry selective extraction (DSE),^23^ and chemical scissor-mediated structural editing of MXenes^24^ coupled with a systematic substitution of weakly bonded surface terminations with other elements^25^ enabled the introduction of P, S, Cl, Se, Br, Sb, Te, and I surface terminations. The reported computational studies, however, still largely focus on O and F terminations or even assume nonterminated MXene surfaces.

Thus, a substantial portion of demonstrated and hypothetical MXene compositions remains unexplored, prompting experimentalists to rely on heuristic approaches. To provide theory-grounded guidance to experimentalists, it is crucial to systematically sample the full compositional space of MXenes to identify synthesizable MXene compositions, predict their physical and chemical properties, and determine the most suitable compositions for specific applications. To fill the gap between the experimental reality and theoretical predictions in the literature, it is essential to account for the diverse surface chemistry of MXenes in the model.

**Figure 1:**
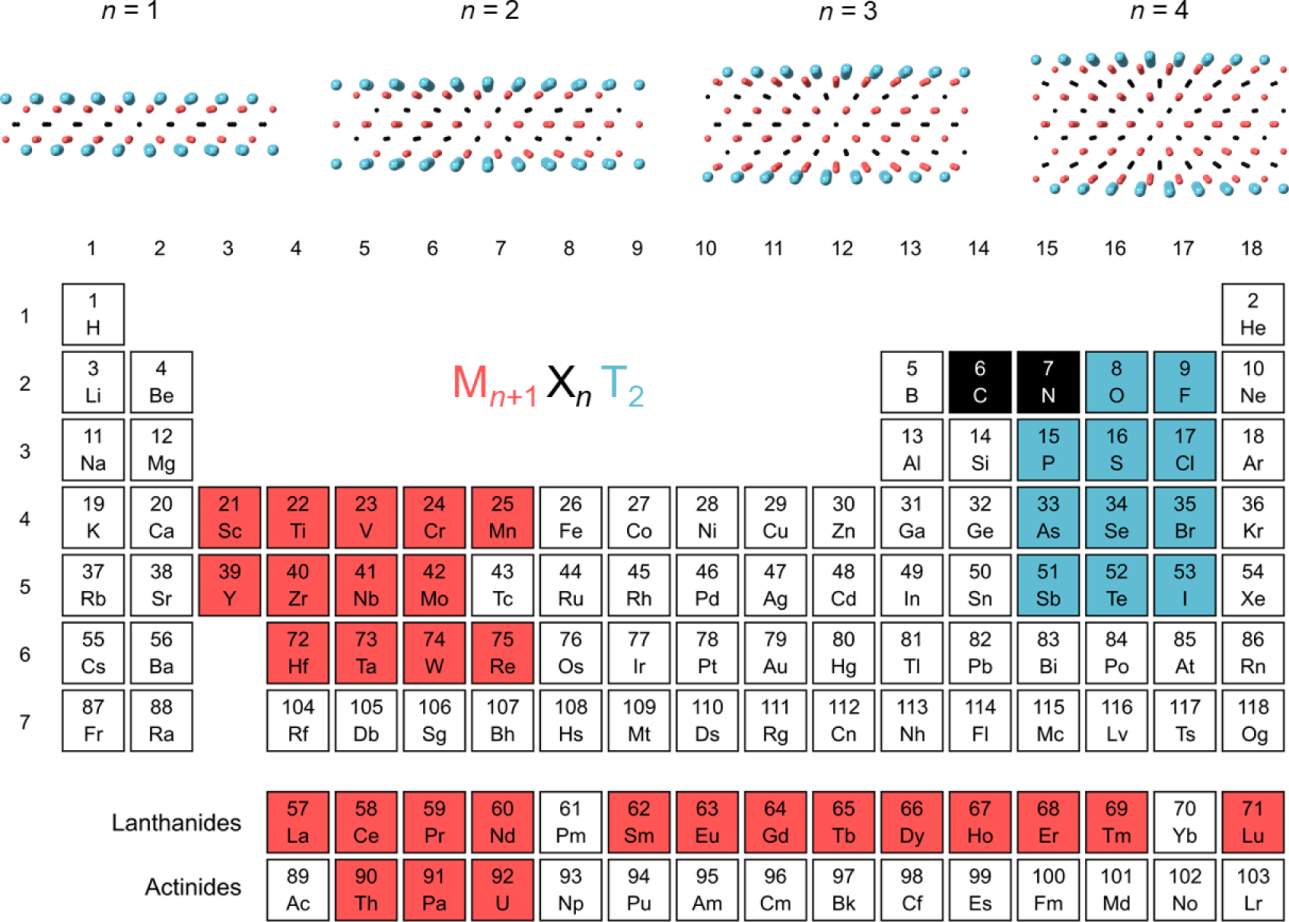
MXene compositional space explored in this work. Transition metals (M) are highlighted in red, carbon and nitrogen (X) in black, and surface terminations (T) in blue. Parameter *n*, which defines the number of transition metal and carbon/nitrogen layers, can be 1, 2, 3, or 4.

Here, we report a systematic assessment of thermodynamic stability across 2784 MXene chemical compositions, M*_n_*_+1_X*_n_*T*_x_*. The overview of compositional space considered in the work is shown in Figure 1 and includes 29 transition metals (M) and 11 surface terminations (T). M sites correspond to the metals of groups 3, 4, 5, 6, and 7, including the *f*-block elements. Note that elements with no stable isotopes are excluded. Additionally, Yb is not considered for methodological reasons (S1). Different chemistry of T sites (*T* = O, F, P, S, Cl, As, Se, Br, Sb, Te, I) as well as nonterminated surfaces are considered. Both carbides and nitrides are included (X = C, N), while *n* can be 1, 2, 3, or 4. Note that we consider a single M, single T, and single X MXenes in 4 basic structures, i.e., we disregard solid-solution and in-plane or out-of-plane ordered bimetal MXenes. We validate the results of the screening against experimental data and existing predictions. Next, we analyze the results to elucidate the factors affecting the thermodynamic stability of MXenes. Then, we identify and discuss thermodynamically (meta)stable MXene compositions. Finally, we give perspectives for further research on MXene synthesis.

## 2 Methodology

A specific MXene structure is thermodynamically stable if its ground-state energy cannot be lowered by separation into competing three-dimensional (3D) phases with the same average composition. Whether MXene can lower its energy by separating into a linear combination of materials with the same average composition is assessed through the convex hull analysis. Within the convex hull formalism, the ground state energies of all polymorphs in a specified chemical space are used to form the lower convex envelope joining these points in energy–composition space. This envelope is termed the convex hull of stability. The distance between the MXene ground state energy and the convex hull of stability quantifies its (meta)stability.^9,26^

In this work, the structure of each MXene composition is generated with three possible configurations of surface termination atoms (T), i.e., hcp (hexagonal close packing), fcc (face-centered cubic), and top. The lowest-energy surface configuration is taken as the ground state energy for each MXene composition. Note that all MXene structures adhere to the traditional structure type (rather than the twinned structure type)^27^ to ensure comparability among MXenes with varying thicknesses (*n*). The corresponding competing phases are determined from compositional phase diagrams. A phase diagram for every M–X–T chemical space is generated using the phase diagram code^28,29^ of the pymatgen library^30^ based on the DFT-computed data available in the Materials Project database.^31^ The structures and the ground state energies of the convex-hull materials closest to the MXene composition (i.e., the structures of the materials forming the lowest energy mixture at the corresponding average composition) are then determined. The energy above the hull Δ*E*_hull_ is computed as
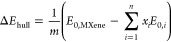
1where *m* is the number of atoms in the MXene supercell, *E*_0,MXene_ is the DFT ground state energy of MXene, *E*_0,*i*_ is the DFT ground state energy of a competing bulk phase, and *x*_*i*_ is the stochiometric coefficient.

**Figure 2:**
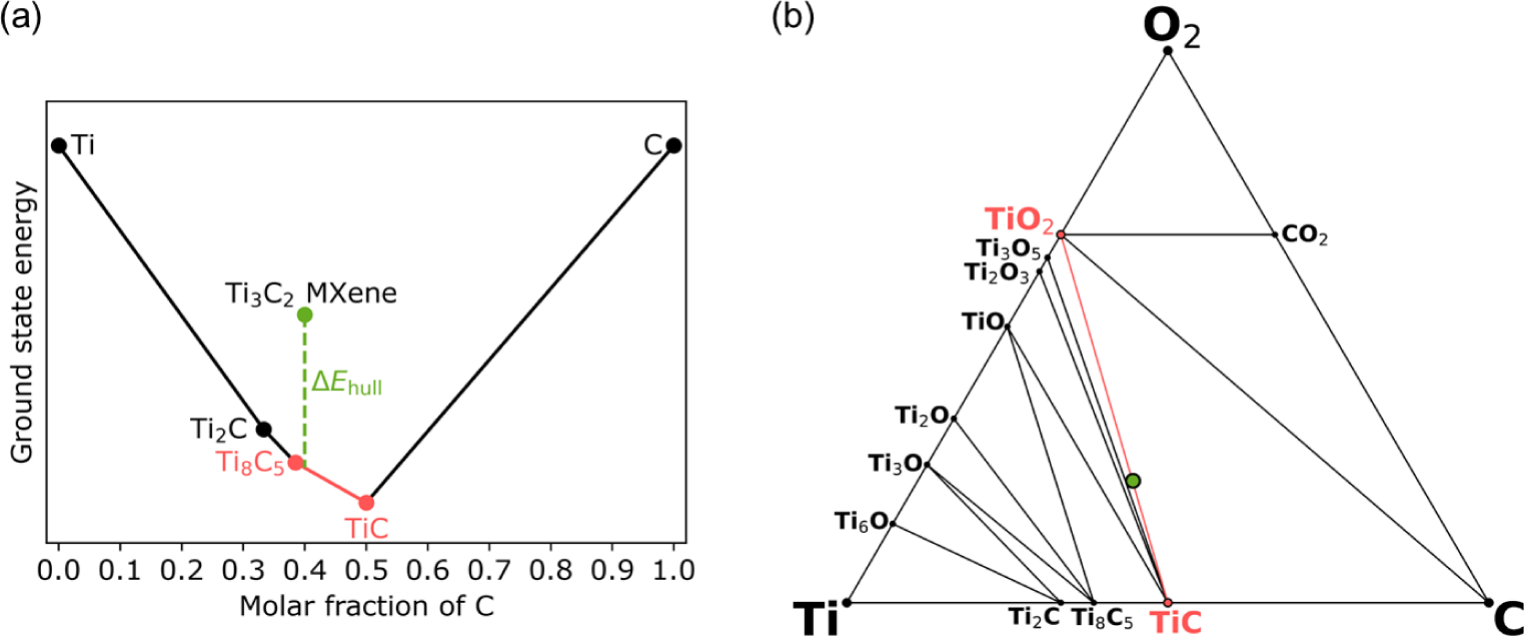
Computation of the energy above energy convex hull for (a) Ti_3_C_2_ and (b) Ti_3_C_2_O_2_ MXenes based on the convex hull binary Ti–C and ternary Ti–C–O phase diagrams, respectively. Black and red circular markers indicate materials on the hull, while green circular marker corresponds to MXene composition. The red color highlights materials on the hull closest to the MXene composition. The (a) vertical or (b) out-of-plane distance between the MXene composition marker and the hull is Δ*E*_hull_.

For instance, to evaluate the thermodynamic stability of nonterminated Ti_3_C_2_ MXene, we construct the Ti–C binary phase diagram (Figure 2a). First, all entries of the Materials Project database of the Ti–C chemical systems are retrieved (81 structures). Second, their DFT-computed ground state energies reported in the database are used to construct the compositional phase diagram based on the convex hull of stability (5 structures on the hull). Third, the composition of the Ti_3_C_2_ MXene is located on the phase diagram (green marker), and the most adjacent structures on the convex hull are identified, i.e., Ti_8_C_5_ and TiC. The corresponding structures are structurally relaxed, and their ground state energies are computed (*vide infra*). Δ*E*_hull_ is then computed as the distance between the ground state energy of Ti_3_C_2_ and the hull, following eq 1, as
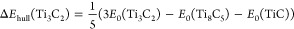
2where *E*_0_(Ti_3_C_2_), *E*_0_(Ti_8_C_5_), and *E*_0_(TiC) are DFT ground state energies (per formula unit) of Ti_3_C_2_, Ti_8_C_5_, and TiC, respectively. Similarly, the assessment of the thermodynamic stability is based on the Ti–C–O ternary phase diagram (Figure 2b). Among 246 structures in the Ti–C–O chemical systems, 14 are forming the convex hull. First, all entries of the Materials Project database in the Ti–C–O chemical systems are retrieved (246 structures). Δ*E*_hull_ is then computed with respect to the linear combination of TiC and TiO_2_ phases. The exact scheme is employed for all MXene compositions. A list of competing phases for all MXene compositions is given in Table S1.

**Figure 3:**
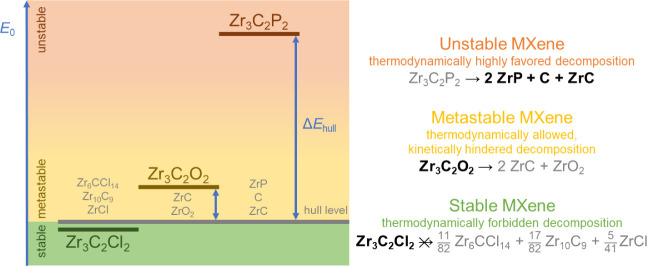
Assessment of the thermodynamic stability of MXenes through the energy above the convex energy hull formalism. MXenes with Δ*E*_hull_ ≤ 0 are stable (marked in green), MXenes with small positive Δ*E*_hull_ are metastable (marked in yellow), and MXenes with large positive Δ*E*_hull_ are unstable (marked in orange).

The value of Δ*E*_hull_ quantifies the thermodynamic stability of a MXene. A lower value of Δ*E*_hull_ indicates a lower thermodynamic driving force for transformation into another material, i.e., higher thermodynamic stability of a given MXene. Figure 3 illustrates the classification of MXenes into stable, metastable, and unstable materials. A MXene with Δ*E*_hull_ ≤ 0 is thermodynamically stable as it corresponds to the state with the lowest ground state energy at the corresponding chemical composition. For instance, the ground state energy of Zr_3_C_2_Cl_2_ is slightly lower than that of the corresponding mixture of Zr_6_CCl_14_, Zr_10_C_9_, and ZrCl, which prevents the decomposition of this MXene. A MXene with a small positive value of Δ*E*_hull_ is likely metastable. This means that the decomposition is thermodynamically allowed but kinetically hindered. Indeed, the first and best-known MXene, Ti_3_C_2_O_2_, is known to be metastable,^9^ similar to most 2D materials.^32^ Another example of such MXene is Zr_3_C_2_O_2_, whose decomposition into the mixture of ZrC + ZrO_2_ is thermodynamically possible but expected to be very slow. A large positive value of Δ*E*_hull_, however, indicates a strong thermodynamic driving force for the decomposition of MXene into other materials. Illustratively, the Zr_3_C_2_P_2_ MXene is predicted to, if hypothetically synthesized, spontaneously decompose into a mixture of ZrP, C, and ZrC and, therefore, classified as unstable. Distinguishing between metastable and unstable materials based on Δ*E*_hull_ is not straightforward and requires some heuristics as the Δ*E*_hull_ threshold, i.e., whether a specific positive Δ*E*_hull_ is deemed small or large, varies among different materials.^32,33^ For 2D materials and MXenes, a Δ*E*_hull_ threshold of 0.2 eV/atom or higher is generally used.^9,34^

DFT calculations are performed using the Vienna Ab initio Simulation Package (VASP)^35^ implementation of the projector augmented wave method.^36^ The generalized gradient approximation functional developed by Perdew, Burke, and Ernzerhof^37^ is chosen to describe the exchange-correlation interactions. The number of valence electrons of the pseudopotentials for different elements is given in Table S2. The energy convergence criterion of the electronic self-consistency is set as 10^–8^ eV. The norms of all the forces after structural relaxation are smaller than 0.01 eV Å^–1^. The tetrahedron method with Blöchl corrections and Gaussian smearing is used to integrate the Brillouin zone for total energy calculations and geometry relaxations, respectively. The width of the smearing is chosen as 0.05 eV. The energy cutoff on the wave function is taken as 600 eV. The conjugate gradient algorithm is employed for structural relaxation. The MXene monolayer is simulated via a supercell with a vacuum layer of at least 14 Å to prevent interactions between the 2D slab and its periodic images. A 12 × 12 × 1 k-point Γ-centered mesh is used for MXene calculations, while the automatic k-mesh generation scheme implemented in VASP with a length of 35 Å is used for other materials. If the automatic k-mesh generation scheme would result in less than 4 k-points, a 3 × 3 × 3 k-point Γ-centered mesh is used instead. Spin-polarized calculations are performed for all materials. Initial magnetic moments for MXenes are set as default in pymatgen,^30^ i.e., 5 μ_B_ for V, Cr, Mn, Mo, Ce, and W, 10 μ_B_ for Eu, and 0.6 μ_B_ for other elements, in a ferromagnetic configuration. Initial magnetic moments of other materials are taken as final magnetic moments reported in the Materials Project database, increased by 50%. Crystal orbital Hamilton population (COHP) analysis^38,39^ is performed using the Local-Orbital Basis Suite Towards Electronic-Structure Reconstruction (LOBSTER package);^40−42^ absolute charge spilling is below 3%.

## 3 Results and Discussion

### 3.1 Δ*E*_hull_ as the Descriptor of MXene Thermodynamic Stability

**Figure 4:**
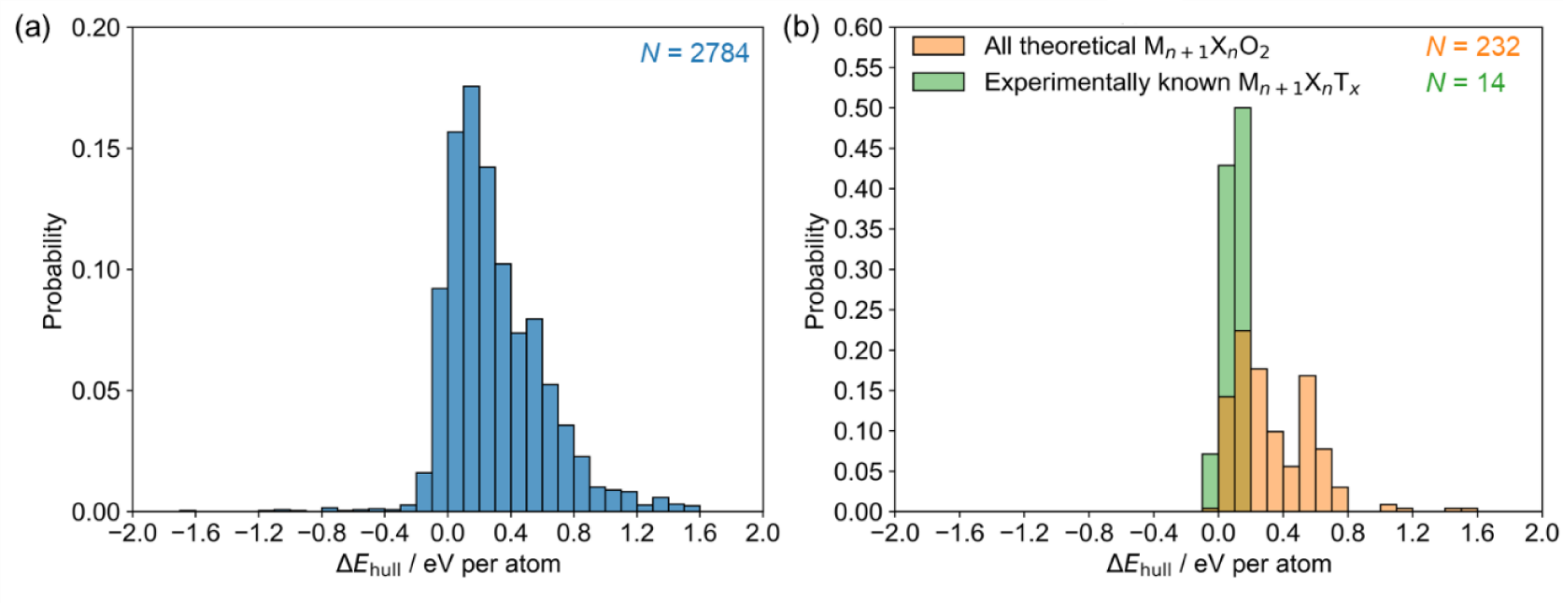
Probability distributions of Δ*E*_hull_ of various MXene compositions. The *N* value indicates the total number of entries for each distribution. Distribution (a) represents the range of MXene compositions studied in this work. Diagram (b) shows a comparison between the distribution of all O-terminated MXenes (M*_n_*_+1_X*_n_*O_2_) examined in this work (orange) and a subset of those compositions. The subset consists of M*_n_*_+1_X*_n_*O_2_ compositions equivalent to M*_n_*_+1_X*_n_*T*_x_* compositions experimentally obtained through aqueous chemical etching (green).

The data computed and analyzed in this work contains 2784 MXene compositions (Figure 1) and their Δ*E*_hull_. The distribution of Δ*E*_hull_ and corresponding basic statistical parameters for all 2784 MXene compositions are provided in Figure 4a and Table 1, respectively. The distribution is unimodal, leptokurtic, and positively skewed. Note that computed values Δ*E*_hull_ might be too low if competing phases are missing in the Materials Project database. Indeed, some of Δ*E*_hull_ values are very negative. This means that the corresponding MXene compositions are more stable than the lowest energy mixture of materials in the Materials Project database and, thus, the MXene composition additionally lowers the hull. Discovery of new structures that lower the hull is possible and expected due to an unavoidable lack of some unknown structures in the databases. However, the distance between the existing and the lowered hull is usually small, making the approach suitable for high-throughput materials discovery.^43,44^

**Table 1:** Basic Statistical Parameters for Distributions Shown in Figure 4

Figure	*N*	Arithmetic mean (eV per atom)	Standard deviation (eV per atom)	First quartile (eV per atom)	Second quartile (eV per atom)	Third quartile (eV per atom)
4(a)	2784	0.31	0.31	0.10	0.24	0.48
4(b) – green	232	0.34	0.25	0.14	0.26	0.53
4(b) – orange	14	0.10	0.05	0.08	0.11	0.13

Next, we consider the Δ*E*_hull_ distribution of experimentally known MXene compositions. Here, we only consider single transition metal MXene compositions synthesized through direct HF etching of MAX-phase precursors or involve MXene treatment in an aqueous medium. Experimentally known compositions with literature references are given in Table S3. The surface layer of these MXene is mainly composed of oxygen atoms,^9,45,46^ Thus, we approximate their compositions to M*_n_*_+1_X*_n_*O_2_. Δ*E*_hull_ distributions of all studied M*_n_*_+1_X*_n_*O_2_ compositions and experimentally known M*_n_*_+1_X*_n_*O_2_ compositions are shown in Figure 4b. The corresponding statistical parameters are provided in Table 1. The Δ*E*_hull_ distribution of experimentally known MXene compositions is shifted toward lower Δ*E*_hull_ values compared to the whole set of O-terminated MXenes. Additionally, the distribution of experimentally known MXenes is significantly narrower (5 times lower standard deviation). Such distribution is expected as low values of Δ*E*_hull_ indicate that the MXene structure is the most thermodynamically stable material at the given composition or very close to the stability of competing phases. These MXene compositions fulfill a necessary condition for synthesizability, i.e., they are thermodynamically (meta)stable.

**Figure 5:**
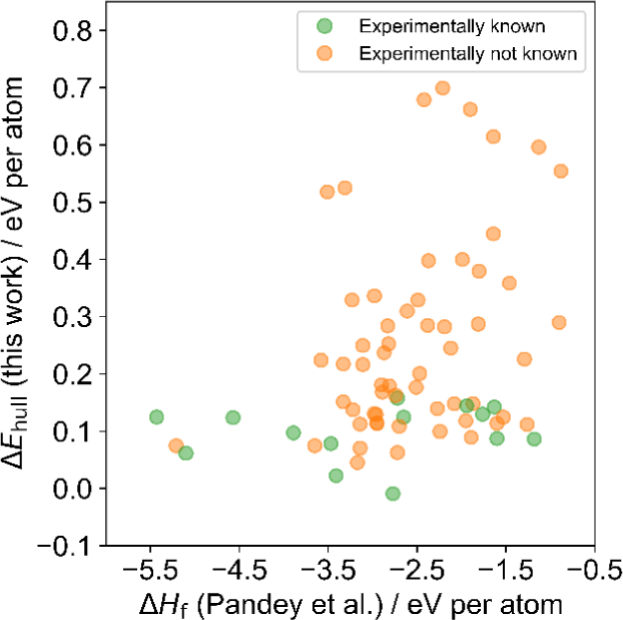
Comparison of M*_n_*_+1_X*_n_*O_2_ heat of formation from the literature Δ*H*_f_ and the energy above the convex energy hull Δ*E*_hull_. Heats of formation are adapted from reference.^47^ Experimentally synthesized MXene compositions are marked in green, while those whose synthesis has not been reported are marked in orange.

Another commonly used descriptor of the thermodynamic stability of a material is the energy of formation Δ*E*_f_ or, similarly, the enthalpy of formation Δ*H*_f_. Δ*E*_f_ (Δ*H*_f_) corresponds to the energy (heat) change during the formation of 1 mol of the substance from its constituent elements in their reference state. The Δ*H*_f_ descriptor was already employed in the stability screening of MXenes with O-, OH-, and F-terminated surfaces.^47^ While this descriptor is easier to compute and does not rely on the comprehensiveness of large materials databases such as Materials Project, its physical relevance is limited. This is because, in the context of thermodynamic stability, materials seldom compete directly with reference-state elements. Figure 5 shows a comparison between literature Δ*H*_f_ (abscissa)^47^ and Δ*E*_hull_ (ordinate) of this work for 72 O-terminated MXene compositions (M*_n_*_+1_X*_n_*O_2_, M ∈ {Sc, Ti, V, Cr, Mn, Y, Zr, Nb, Mo, Hf, Ta, W}, X ∈ {C, N}, *n* ∈ {1, 2, 3}). Both values are normalized to the number of atoms in the MXene supercell. Experimentally known compositions (Table S3) are marked in green, while those not reported are marked in orange. The correlation between Δ*H*_f_ and Δ*E*_hull_ is very weak. In this case, Δ*H*_f_ poorly distinguishes between experimentally known MXene compositions and those not reported to date. Δ*E*_hull_, on the other hand, classifies MXenes into groups of experimentally known and unknown much better. Therefore, it is suitable as an approximate predictive descriptor of MXene thermodynamic stability. Experimentally unknown MXene compositions with low values of Δ*E*_hull_ are present. Note that the absence of these compositions among experimentally reported does not necessarily mean they are impossible to synthesize. For instance, their synthesis has been probed using an unsuitable precursor or synthesis methods or has not has not been experimentally probed at all. Alternatively, they might be thermodynamically stable but not synthesizable. It is also possible that an important stable phase on the convex energy hull is missing in the Materials Project database, which would artificially lower the Δ*E*_hull_ value as the hull level would be inaccurately placed at too high energy values. Still, Δ*E*_hull_ serves as the most suitable descriptor for screening purposes, as established in computational materials science^26^ and herein demonstrated for the case of MXenes.

Every MXene composition can be classified into one of three distinct classes of thermodynamic stability: stable, metastable, or unstable, all determined by their respective Δ*E*_hull_. A composition is deemed stable if Δ*E*_hull_ ≤ 0. In this work, we use the Δ*E*_hull_ of the experimentally known MXene with the highest Δ*E*_hull_ as a guiding threshold to distinguish between metastable and unstable MXene compositions. This threshold of 0.158 eV per atom is attributed to the V_2_CO_2_ MXene composition.

Note that, in addition to thermodynamic stability, dynamic stability is a necessary condition for the overall stability of MXene. The evaluation of dynamic stability relies on compute-intensive phonon calculations or *ab initio* molecular dynamics (AIMD) simulations. These methods are less suitable for screening large compositional spaces, as in the present work. While dynamic stability assessment is not part of this work, it is recommended for narrower, more focused studies on MXenes of particular interest.

### 3.2 Correlations and Trends in MXene Thermodynamic Stability

We first analyze the factors that affect Δ*E*_hull_ and classification of MXene as (meta)stable or unstable. Here, we disregard compositions with nonterminated surfaces. First, the existence of linear or monotonic correlations between Δ*E*_hull_ and basic characteristics of MXene compositions is explored through Pearson and Spearman’s correlation coefficients, respectively. These coefficients range from −1 to 1, where −1 indicates a perfect negative correlation, 0 indicates no correlation, and 1 indicates a perfect positive correlation. In this analysis, we specifically consider basic atomic characteristics of M, X, and T (electronic affinity *E*_a_, first ionization energy *E*_ie_, Pauling electronegativity χ, van der Waals radius r, atomic number Z, and Mendeleev number MN^48^ and the parameter *n*. No strong direct relationship between these variables is observed. However, weak linear correlations are observed between Δ*E*_hull_ and *E*_a,T_, MN_T_, and χ_T_. Pearson correlation coefficients are −0.46, –0.36, and −0.32, respectively, indicating that higher values correlate with lower Δ*E*_hull_, i.e., higher stability. Similar behavior is observed for monotonic correlation (Spearman’s ρ of −0.49, –0.36, and −0.32, respectively). The negative correlation between *E*_a,T_ and Δ*E*_hull_ is reasonable, given that a higher electronic affinity of the surface termination atom leads to stronger electrostatic interaction between the transition metal and the surface termination. Other parameters have absolute values of Pearson correlation coefficient and Spearman’s ρ lower than 0.3, indicating no or very weak linear or monotonic correlation. However, the effect of transition metal on the thermodynamic stability of MXene is well-known.^1,9^ This indicates a complex, nonmonotonic relationship between those parameters and the stability of MXene.

To explore this complex relationship, we simplify the problem to distinguishing between (meta)stable and unstable compositions (i.e., classification instead of regression). We identify parameters that highly correlate with the stability class (unstable or (meta)stable) and build a simple decision tree model. The most powerful parameters for decision rules are *E*_a,T_, MN_T_, χ_T_, r_T_, and MN_M_, with gain ratios of 0.06, 0.05, 0.05, 0.03, and 0.02, respectively. Note that the information gain and Gini coefficient scorings give the same ranking of the top five parameters. Some parameters contain overlapping information (e.g., Mendeleev number and electronegativity are highly correlated) and are unlikely to form the foundation for additional decision rules. To identify additional decision rules, we build a simple decision tree as implemented in Orange.^49^

**Figure 6:**
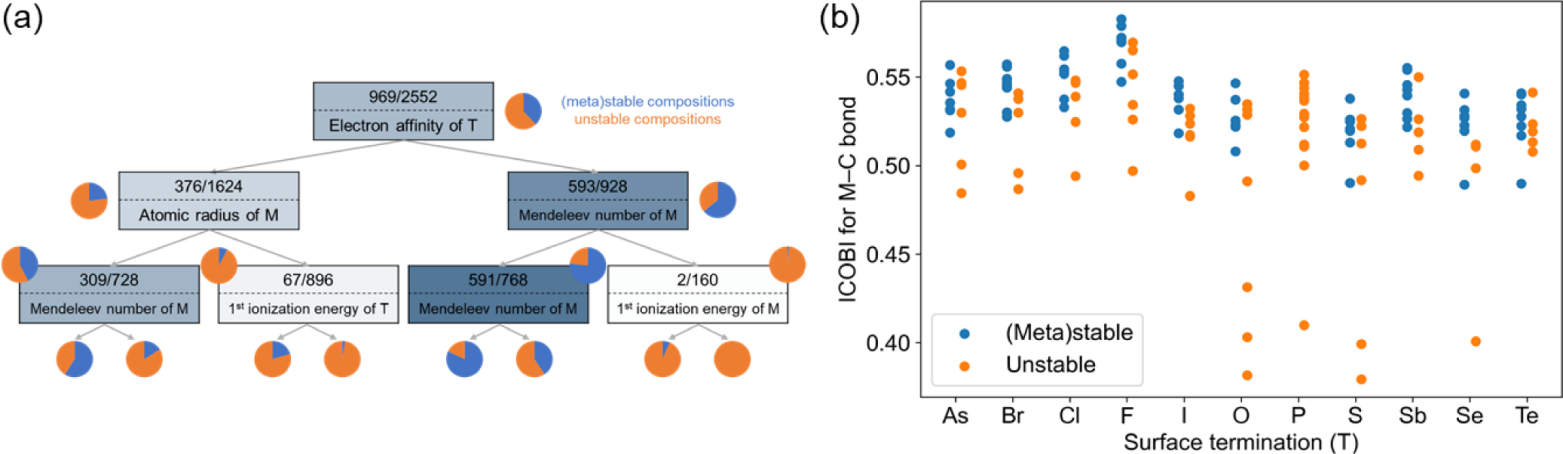
Understanding the trends in MXene thermodynamic stability: (a) The first three node levels of the decision tree for classifying MXenes as thermodynamically (meta)stable or unstable. M and T indicate a transition metal and a surface termination atom, respectively. (b) Integrated Crystal Orbital Bond Index for M–C bond as a function of surface termination for a subset of M_2_CT_2_ MXenes (only M of d block are considered).

The first tree node layers of the tree are illustrated in Figure 6a. The first four node layers are shown in Figure S1, and the performance metrics are summarized in Table S4. Importantly, the first decision rule in the tree is the *E*_a,T_. The decision rules on the second level are r_M_ and MN_M_. This demonstrates the high importance of both T and M for overall stability and shows some predictive power of simple characteristics of constituent atoms. Note that the parameter *n* and characteristic associated with X would only appear in the fourth layer of decision nodes, indicating worse predictive power of X and *n*.

The structure of materials is governed by the chemical bond between different atoms. We further explore the impact of interaction among different basic atomic characteristics of M, X, and T on the decision rules of a decision tree. Thus, we build chemically informed interacting parameters and use them to build a decision tree. The full list of tree parameters is given in Table S5. The first four node layers of the tree are shown in Figure S2, and key performance metrics are summarized in Table S6. Now, the most powerful parameters for decision rules are *E*_i,M_ – *E*_ea,T_, *E*_ea,X_ • *E*_ea,T_, χ_M_ – χ_T_, *E*_ea,M_ • *E*_ea,T_, and MN_X_ – MN_T_ with gain ratios of 0.10, 0.04, 0.03, 0.03, and 0.03, respectively. Again, the information gain and Gini coefficient scorings give the same ranking of the top five parameters. The resulting decision tree performs slightly better than the first tree (F_1_ scores are 0.917 and 0.926, respectively). More importantly, *E*_i,M_ – *E*_ea,T_ (i.e., the difference between the first ionization energy of the transition metal and the electron affinity of the surface termination) forms a more powerful decision rule than the *E*_a,T_ alone (compare first decision nodes in Figures 6 and S2).

We additionally verify the importance of parameters through a permutation feature importance analysis for both decision tree models. In the first decision tree (Figure 6a), the electron affinity of the surface termination is identified as the most important feature (0.316 ± 0.007 decrease in AUC, i.e., area under the receiver operating characteristic curve). In the second decision tree (Figure S2), the difference between the first ionization energy of the transition metal and the electron affinity of the surface termination is the most important feature (0.251 ± 0.005 decrease in AUC). The five most important features, i.e., those with the highest decrease in AUC for each of the trees, are provided in Tables S7 and S8.

The nature of chemical bonding is evaluated through the crystal orbital Hamilton population (COHP) analysis on an M_2_CT_2_ (only M of the d block are considered) subset. The influence of the strength of covalent and ionic bonding onto the Δ*E*_hull_ is evaluated through the sum of integrated crystal orbital Hamilton populations (ICOHP) and Madelung energy based on Löwdin partial charges, respectively. The absolute value of Pearson and Spearman correlation coefficients is lower than 0.1, showing no meaningful correlation with the covalency or ionicity of the MXene (see also Figure S3). Further, we analyze the correlation of Δ*E*_hull_ with ICOHP and integrated crystal orbital bond index (ICOBI)^50^ of M–C, M–T, and C–T bonds, Löwdin partial atom charges, as well as total ICOHP and Madelung energy. Here, ICOBI closely relates to the concept of the covalent bond order. The Δ*E*_hull_ correlates most strongly with M–C ICOBI and T–C ICOBI with Pearson coefficients of −0.46 and 0.36 and Spearman’s ρ of −0.47 and 0.54, respectively. The correlation of both ICOBI outperforms simple atomic characteristics used above, i.e., *E*_a,T_, MN_T_, and χ_T_. Their Pearson correlations with Δ*E*_hull_ on this subset are −0.34, – 0.21, and −0.19, respectively. Similarly, Spearman’s ρ are −0.35, – 0.17, and −0.12, respectively. Additionally, we note that (meta)stable MXenes are characterized by sufficiently high values of M–C ICOBI (Figure S4a) and sufficiently low values of C–T ICOBI (Figure S4b). On the other hand, no such requirement is observed for M–T ICOBI (Figure S4c), which spans across a wide range of values (0.21 < ICOBI_MT_ < 0.67). Importantly, the surface termination chemistry also significantly influences the bonding between the transition metal and carbon (M–C ICOBI), impacting the stability of the MXene (Figure 6b). Indeed, Pearson correlation coefficient between ICOBI_MC_ and ICOBI_MT_ is −0.37, indicating a moderate negative linear correlation.

The thermodynamics of MXenes, which compete with other possible phases in the same chemical space to form the most stable form, is too complex to be fully captured by descriptors used herein. Nevertheless, this analysis shows that surface terminations play a pivotal role in the overall stability of the MXene sheet.

**Figure 7:**
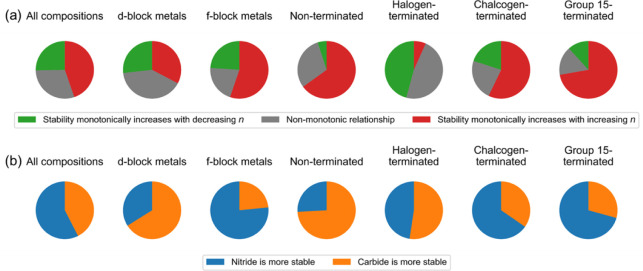
Effect of X and *n* parameters on the thermodynamic stability of M*_n_*_+1_X*_n_*T_2_ compositions described by Δ*E*_hull_. In (a), the green color indicates a share of compositions for which the thermodynamic stability increases monotonically with decreasing *n*, i.e., the thermodynamic stability increases in the order M_5_X_4_T_2_ < M_4_X_3_T_2_ < M_3_X_2_T_2_ < M_2_XT_2_, and *vice versa* for the red color. The gray color indicates a nonmonotonic relationship between *n* and Δ*E*_hull_ at fixed parameters M, X, and T. In (a), the blue color indicates a share of compositions for which the nitride is more stable than its carbide analog, and *vice versa* for the orange color. In both (a) and (b), the 1st pie chart corresponds to all MXene compositions, the 2nd and 3rd pie charts discriminate compositions based on the position of M in the d or f block of the periodic table, and the 4th through 7th pie charts discriminate compositions based on the chemistry of the T site.

The thicker MXenes, i.e., those with higher *n*, are sometimes expected to be more thermally stable as their structure resembles the structure of cubic carbides with a higher number of strong M–X bonds. However, the experimental analysis of their thermal stability shows that this is not a general rule that applies to any MXene material.^27,51^ We analyze the relationship between the computed Δ*E*_hull_ and the parameter *n* for a fixed set of M, X, and T. The results are shown in Figure 7a. In 70% of compositions, the relationship between Δ*E*_hull_ and *n* is monotonic. Whether the correlation is positive or negative, however, highly depends on the chemical composition of MXene. In the case of MXene based on f-block metals, thicker MXenes are thermodynamically preferred more often. On the other hand, the share of positive and negative correlations is approximately balanced (28% and 30%, respectively). If only MXenes with a nonterminated surface are considered, thicker MXenes are generally preferred. On the other hand, the termination of MXene sheets with halogen surface terminations reverses this trend to generally increased stability of thinner MXenes. The effect of surface terminations in thinner MXenes is stronger due to a higher ratio of surface termination in overall MXene composition. As MXenes with halogen terminated surfaces are indeed the most prevalent among (meta)stable MXenes (see Figure 9 for more details), this observation could be attributed to the more pronounced effect of stabilization through halogen termination of MXene sheets in thinner MXenes.

The effect of the X site chemistry, i.e., carbide versus nitride, is analyzed similarly. The results are shown in Figure 7b. In 57% of all compositions, nitride MXenes are thermodynamically preferred. This seems counterintuitive due to the strong prevalence of carbides among experimentally known MXenes. However, among MXenes with d-block elements on transition metal sites (f-block chemistry has yet to be experimentally explored within the MXene community), carbides are thermodynamically preferred to nitrides in 66% of cases. Thus, f-block MXenes could be a leap forward in expanding the family of MXene nitrides. Notably, the nitride MXenes are preferred among MXenes with chalcogen- and group-15-terminated surfaces, while this preference vanishes in halogen-terminated MXenes.

### 3.3 Overview of Thermodynamically (Meta)stable MXene Compositions

**Figure 8:**
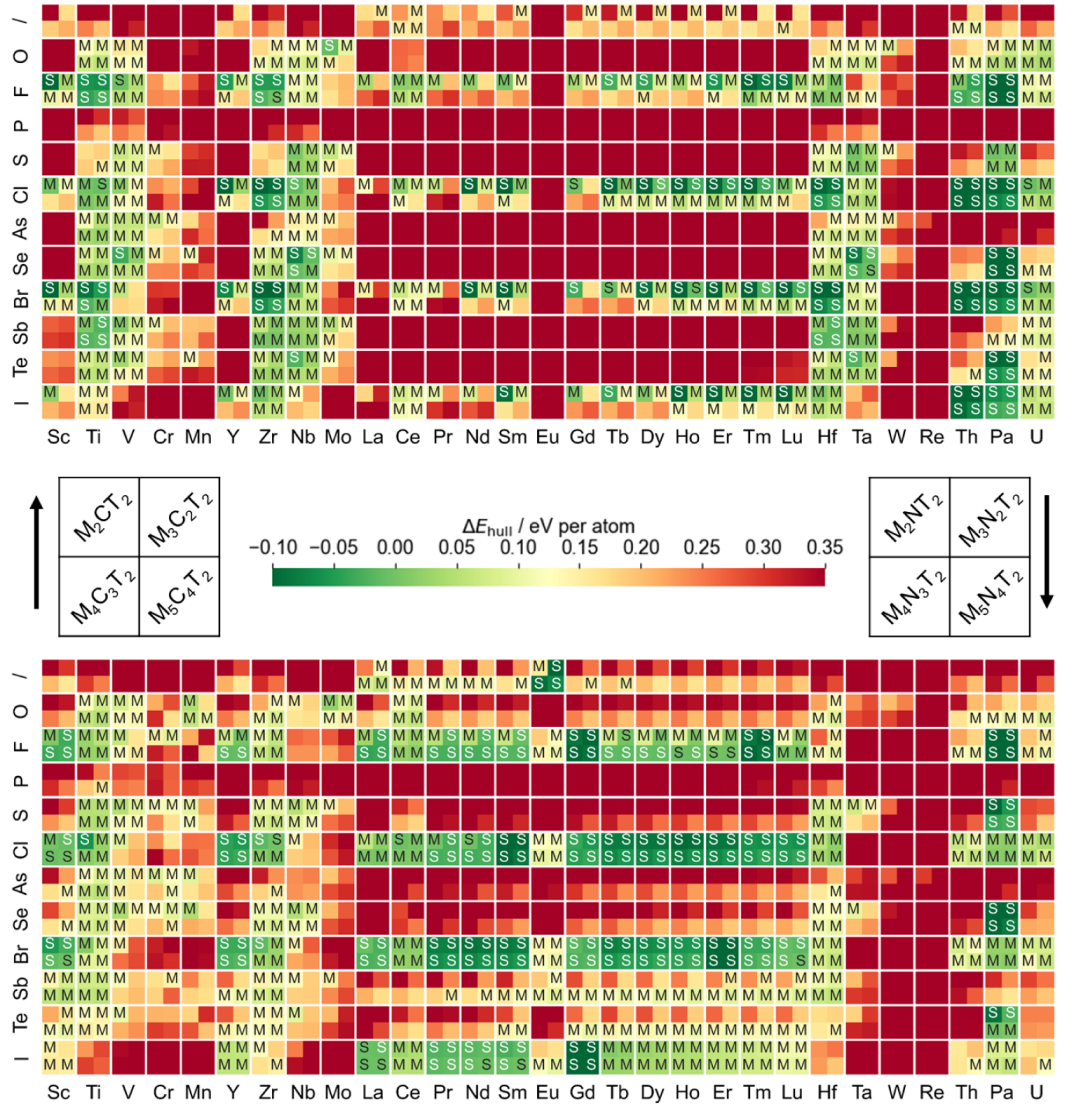
Heatmap of the energy above the hull Δ*E*_hull_ for all M*_n_*_+1_X*_n_*T_2_ compositions considered in this work. Columns and rows indicate the element on the transition metal (M) site and surface termination (T) site, respectively. The symbol “/” indicates MXenes with a nonterminated surface. The upper (lower) part corresponds to carbide (nitride) MXenes. Each M–T box shows Δ*E*_hull_ for four possible values of the parameter *n*: 1 (upper left), 2 (upper right), 3 (lower left), or 4 (lower right). The color indicates the Δ*E*_hull_ value. Compositions with Δ*E*_hull_ < – 0.10 eV per atom are colored as −0.10 eV per atom, and compositions with Δ*E*_hull_ > 0.35 eV per atom are colored as 0.35 eV per atom. Thermodynamically stable (Δ*E*_hull_ ≤ 0) and metastable compositions (0 < Δ*E*_hull_ ≤ 0.158 eV per atom) are labeled with letters S and M, respectively.

Δ*E*_hull_ values for all MXene compositions screened in this work are illustrated in Figure 8. The slash (/) indicates MXenes with a bare surface, i.e., no surface termination (T) atoms. Compositions, marked with letters S and M, are predicted to be thermodynamically stable and thermodynamically metastable, respectively. Among 2784 screened M*_n_*_+1_X*_n_*T_2_ compositions, 695 are predicted to be metastable and 303 to be stable, totaling 998 (meta)stable compositions. Additionally, 686 MXene compositions are predicted to be more stable than Ti_3_C_2_O_2_.

**Figure 9:**
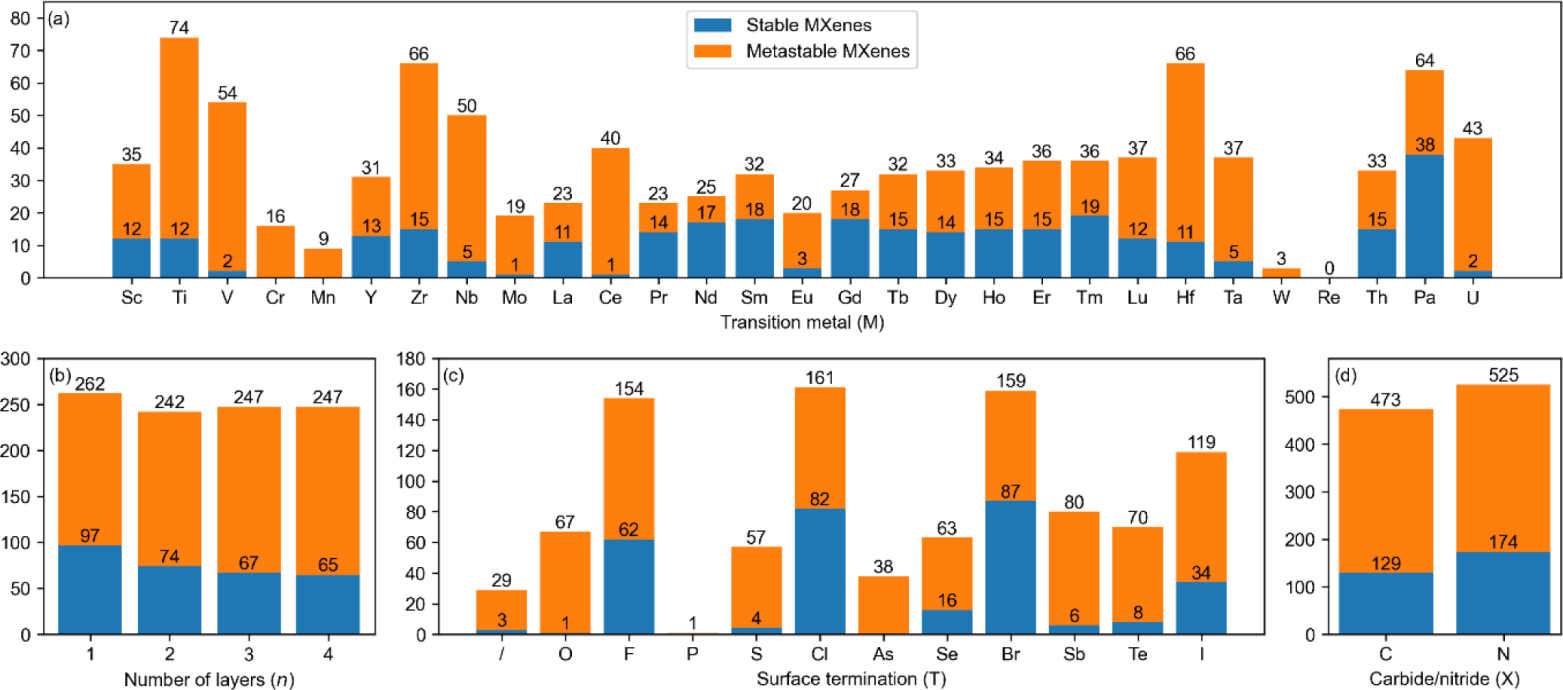
Number of stable (blue) and metastable (orange) MXenes composed of (a) specific transition metals (M) or (b) surface terminations (T). The symbol “/” indicates MXenes with a nonterminated surface. The number of stable and metastable MXenes with respect to the number of layers (*n*) and carbide/nitride MXenes are shown in (b) and (d), respectively. The number above the blue bar indicates the number of stable compositions. The number above the orange bar indicates the total number of metastable and stable compositions.

The number of stable and metastable M*_n_*_+1_X*_n_*T_2_ compositions depending on parameters M (transition metal), *n* (number of layers), T (surface termination), and X (carbide/nitride) is shown in Figure 9. The number of (meta)stable compositions does not vary significantly across different values of *n* or X. However, within a specific M–T combination, both *n* and X can greatly influence the Δ*E*_hull_.

The chemistry of M sites strongly affects the number of (meta)stable chemical compositions. Interestingly, Ti is the transition metal with the highest number of (meta)stable MXene compositions. Furthermore, Ti-based MXenes are unique in their ability to form at least one metastable composition with any surface terminations examined herein. Generally, the transition metals of group 4 of the periodic table (Ti, Zr, Hf) form the highest number of (meta)stable MXene compositions (206 (meta)stable compositions). Group 5 of the periodic table (V, Nb, Ta) also stands out, with many MXene compositions predicted to be (meta)stable (141 compositions). Indeed, all transition elements of groups 4 and 5 with stable isotopes are present among experimentally known single-atom MXene compositions (Table S3).

A recent perspective on the future of MXenes highlights the gap between the chemical diversity of transition metals in MAX phases and MXenes discovered to date.^52^ Specifically, Mn and all lanthanides, except La, Pm, and Eu, are found in experimentally synthesized MAX phases. Yet, no MXene composition with any of these transition metals was experimentally reported. We classify many MXene compositions with these transition metals as (meta)stable. Specifically, Mn_2_NO_2_, Mn_4_N_3_O_2_, Mn_5_N_4_O_2_, Mn_2_NS_2_, Mn_4_N_3_S_2_, Mn_2_NAs_2_, Mn_2_NSe_2_, Mn_2_CSe_2_, and Mn_2_CTe_2_ are predicted as metastable Mn MXene compositions. Note that Mn_2_NO_2_, for example, has already been identified as an MXene with highly desirable ferromagnetic ground states, Curie temperature well above room temperature,^53^ and a promising anode material for metal ion batteries.^54^ Its synthesis, however, has not been reported to date. Despite the chemical similarity between Re and Mn, Re is predicted to form no (meta)stable single transition metal MXene compositions. It might be possible, however, that Re can be introduced into the mixed-metal MXene structure. The interesting catalytic properties of Re compounds^55^ make this strategy worth exploring.

All elements of the lanthanide series, except Pm and Yb, are identified to form (meta)stable MXenes (note that Pm and Yb are excluded from our screening; see *Methodology*). This includes La and Eu, which were not reported in any of the experimentally known MAX phases.^56^ However, all (meta)stable Eu-based MXene compositions and 19 out of 22 (meta)stable La-based MXene compositions are nitride MXenes, while all lanthanide-based MAX phases were reported as carbides. This suggests that extending the lanthanide-based MAX phases to nitrides might unlock La and Eu MAX phases and, potentially, La and Eu MXenes. O-terminated lanthanide MXenes are predicted to be unstable (except metastable Ce*_n_*_+1_N*_n_*O_2_ compositions). Instead, (meta)stable lanthanide MXenes are mostly halogen terminated (F, Cl, Br, I). This implies that Lewis molten salt or halogen etching might be a more suitable method for synthesizing lanthanide-based MXenes. For instance, molten transition metal halide salts, such as CuCl_2_, or elemental halogens could be used to etch the A-layer of lanthanide-based MXenes to form halogen-terminated MXenes. Distinctive properties of lanthanides arise from their electronic structure with up to 7 unpaired electrons in the 4f subshell. As a result, lanthanide compounds are used in catalysts, phosphors, and magnets. DFT calculations could serve as a highly suitable approach for gaining insights into the catalytic properties and magnetism of lanthanide MXenes.

Th, Pa, and U, i.e., are the only elements of the actinide series with stable isotopes, all of which form (meta)stable MXene compositions. The surface of (meta)stable actinide MXene compositions can be capped with halogens or oxygen, and in the case of Pa, also with other chalcogens. Actinide MXenes would not be suitable for daily life application due to the radioactivity and toxicity of actinides. They might be, however, interesting for niche scientific applications.

**Figure 10:**
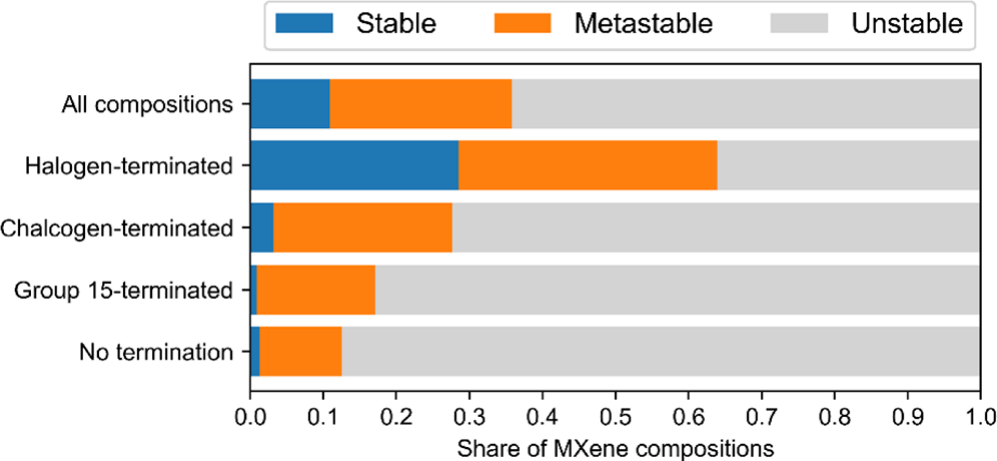
Share of thermodynamically stable, metastable, and unstable MXene compositions depending on the chemical nature of the surface termination atom.

The effect of the T element on the overall stability is very pronounced. The distribution of the number of stable, metastable, and unstable compositions for different groups of T is shown in Figure 10. MXenes with nonterminated surfaces are rarely (meta)stable. While surface termination of MXenes is sometimes seen as undesirable due to its impact on some of MXene properties,^57^ it is evident that the presence of surface termination groups generally increases the inherent thermodynamic stability of MXenes.

MXenes terminated with halogen atoms are more often classified as (meta)stable than group 15- and chalcogen-terminated MXenes. All halogen elements are identified in many different (meta)stable MXene compositions. This once again reinforces the interest in the synthesis of MXenes through molten salt etching and the DSE. MXenes with Br and I terminations were identified as promising materials for battery cathodes due to the relatively weak bonding between M and T atoms.^4^ Exploring halogen-terminated MXenes beyond Ti, many of which are predicted as (meta)stable, provides an opportunity for the systematical tuning of the strength of the M–T bond. This opens new horizons for the increase in performance of such batteries.

The number of (meta)stable chalcogen-terminated MXenes is somewhat lower, mainly due to the instability of f-block chalcogen-terminated MXenes. On the other hand, all chalcogenides (O, S, Se, Te) yield many different (meta)stable compositions with d-block metals that are interesting for further research. For instance, termination of Nb_2_C with S or Se was identified as favorable for superconductive behavior, while no superconductivity was observed in the case of O-terminated MXene.^25^ Chalcogen-terminated MXenes could be synthesized through the substitution of Cl/Br/I surface terminations of MXenes produced via Lewis molten salt etching or through the chemical scissor-mediated editing approach.^24^

MXenes terminated with atoms of group 15 of the periodic table (P, As, Sb) differ in the number of (meta)stable MXene compositions. P-terminated surface results in only one MXene classified as metastable, i.e., Ti_5_N_4_P_2_. Still, the synthesis of a Ti_3_C_2_(P_0.4_Br_0.6_)*_x_* MXene has been reported,^24^ demonstrating the possibility of a partially P-terminated surface by mixing different terminations. While no MXene with an As-terminated surface has been reported, probably due to the high toxicity of As and its compounds, we classify 38 As-terminated MXene compositions as metastable. Sb yields the highest number of (meta)stable MXenes among all nonhalogen-terminated MXenes and is the only element of group 15 that forms metastable lanthanide MXenes.

The dependence of MXene thermodynamic stability on the chemistry of surface terminations means that the stability of a certain M*_n_*_+1_X*_n_* composition can be tuned through MXene surface chemistry. Specifically, if the O, which is the most pronounced termination in MXenes synthesized through aqueous chemical etching, is substituted with another T atom, the same M*_n_*_+1_X*_n_* composition can transit from metastable to stable or from unstable to metastable state. This effect is most often observed for Cl and Br terminations. This suggests that the synthesis of M*_n_*_+1_X*_n_* compositions that are not accessible through aqueous etching might be possible through molten salt etching, gaseous halogen etching, or chemical vapor synthesis.

## 4 Conclusions

A wide range of hypothetical MXene structures have been screened to elucidate factors affecting their thermodynamic stability and identify stable MXene compositions. We have combined the database DFT data to construct 638 compositional phase diagrams and performed high-throughput DFT calculations of 7888 MXene structures as well as 687 thermodynamically competing phases to assess the thermodynamic stability of the MXene family. The pivotal role of the surface termination chemistry in the overall thermodynamic stability of MXenes has been shown, and many new (meta)stable MXene compositions across the wide MXene chemical space have been identified. This work lays the foundation for the materials genomics^31,58^ of MXenes, i.e., MXene genomics.

Further research should elucidate the synthesizability of (meta)stable compositions through the identification of the most suitable synthesis methods, precursors, reagents, and reaction conditions, combining first-principles calculations and the existing experimental knowledge, as reported for selective etching of layered solids recently.^59^ While many chemical and physical properties of MXenes can be efficiently predicted using a variety of simulation methods on different time and length scales, some properties required for target applications are too complex to be simulated computationally. In such instances, a synergistic approach that combines simulation, automated high-throughput experimentation, and machine learning should be the key to unlocking the full potential of MXenes.

## Supporting Information Available

Details on the exclusion of Yb-based MXenes from the study; list of competing phases used to compute energy above the hull for every MXene composition; the number of valence electrons of the pseudopotentials; list of literature reports on experimentally synthesized MXene composition in aqueous media; fist four node levels of the two decision trees, their performance metrics, and permutation feature importance analysis; list of parameters for building a decision tree with interacting parameters; plots showing energy above the hull vs ICOHP, Madelung energy, and ICOBI for M–C, C–T, and M–T bonds; Madelung energy vs ICOHP plot (PDF).
Structures of MXenes explored in this work with lowest-energy surface configuration in POSCAR format (ZIP).
