# Supplementary material for: Pivotal Role of Surface Terminations in MXene Thermodynamic Stability
Source: Chem Mater. 2024 Oct 11;36(20):10295–306. doi: 10.1021/acs.chemmater.4c02274 (PMC11500292; doi:10.1021/acs.chemmater.4c02274)
Supplement: Supplementary file 1 — cm4c02274_si_001.pdf [file cm4c02274_si_001.pdf]

Supporting Information

Pivotal Role of Surface Terminations in MXene Thermodynamic Stability

Ervin Rems[a,b], Yong-Jie Hu[c], Yury Gogotsi[c,d], Robert Dominko[a,b,e]

[a] National Institute of Chemistry,1000 Ljubljana, Slovenia

[b] Faculty of Chemistry and Chemical Technology, University of Ljubljana, 1000 Ljubljana, Slovenia

[c] Department of Materials Science and Engineering, Drexel University, Philadelphia, PA, 19104, USA

[d] A.J. Drexel Nanomaterials Institute, Drexel University, Philadelphia, PA, 19104, USA

[e] ALISTORE - European Research Institute, CNRS FR 3104, Amiens 80039 Cedex, France

S1: Exclusion of Yb MXenes

The Yb_2 pseudopotential (8 valence electrons) of VASP has been historically used in the DFT calculations within the Materials Project. This pseudopotential leads to incorrect thermodynamics of many systems with Yb in the 3+ oxidation state[1]. As Yb mostly forms compounds with Yb in 3+ oxidation state, this often leads to inaccurate thermodynamic predictions. As a result, all DFT calculations of Yb-containing materials, which were performed using the Yb_2 pseudopotential, were deprecated in the 2023.11.1 version of the Materials Project database[2]. Due to the unavailability of thermodynamics based on Yb_3 pseudopotential (9 valence electrons) calculations and unreliable thermodynamics based on the legacy Yb_2 pseudopotential calculations, we excluded Yb-containing compounds from this study.

Table S1: Competing phases of MXene composition used to compute energy above the hull

**MXene composition**	**Competing phases**		
**Sc_2C**	Sc_2C		
**Sc_2CO_2**	Sc_2CO	Sc_2O_3	C
**Sc_2CF_2**	Sc_4C_3	ScF_3	
**Sc_2CP_2**	ScP	C	
**Sc_2CS_2**	C	ScS	
**Sc_2CCl_2**	Sc_2CCl_2		
**Sc_2CAs_2**	ScAs	C	
**Sc_2CSe_2**	C	ScSe	
**Sc_2CBr_2**	Sc_3C_4	Sc_7CBr_{12}	Sc_4C_3
**Sc_2CSb_2**	ScSb	C	
**Sc_2CTe_2**	Sc_3C_4	Sc_2Te_3	C
**Sc_2CI_2**	Sc_2CI_3	Sc_7CI_{12}	Sc_4C_3
**Sc_3C_2**	Sc_2C	Sc_4C_3	
**$Sc_3C_2O_2$**	Sc_2CO	Sc_2O_3	C
**$Sc_3C_2F_2$**	Sc_3C_4	Sc_4C_3	ScF_3
**$Sc_3C_2P_2$**	Sc_3C_4	ScP	C
**$Sc_3C_2S_2$**	Sc_3C_4	C	ScS
**$Sc_3C_2Cl_2$**	Sc_2CCl_2	Sc_3C_4	Sc_4C_3
**$Sc_3C_2As_2$**	Sc_3C_4	ScAs	C
**$Sc_3C_2Se_2$**	Sc_3C_4	C	ScSe
**$Sc_3C_2Br_2$**	Sc_3C_4	Sc_7CBr_{12}	Sc_4C_3
**$Sc_3C_2Sb_2$**	Sc_3C_4	ScSb	C
**$Sc_3C_2Te_2$**	ScTe	Sc_3C_4	Sc_2Te_3
**$Sc_3C_2I_2$**	Sc_3C_4	Sc_2CI_3	Sc_4C_3
**Sc_4C_3**	Sc_4C_3		
**$Sc_4C_3O_2$**	Sc_2CO	C	
**$Sc_4C_3F_2$**	Sc_3C_4	Sc_4C_3	ScF_3

**$Sc_4C_3P_2$**	Sc_3C_4	ScP	C
**$Sc_4C_3S_2$**	Sc_3C_4	C	ScS
**$Sc_4C_3Cl_2$**	Sc_2CCl_2	Sc_3C_4	Sc_4C_3
**$Sc_4C_3As_2$**	Sc_3C_4	ScAs	C
**$Sc_4C_3Se_2$**	Sc_3C_4	C	ScSe
**$Sc_4C_3Br_2$**	Sc_3C_4	Sc_7CBr_{12}	Sc_4C_3
**$Sc_4C_3Sb_2$**	Sc_3C_4	ScSb	C
**$Sc_4C_3Te_2$**	ScTe	Sc_3C_4	Sc_2Te_3
**$Sc_4C_3I_2$**	Sc_3C_4	Sc_2CI_3	Sc_4C_3
**Sc_5C_4**	Sc_3C_4	Sc_4C_3	
**$Sc_5C_4O_2$**	Sc_3C_4	Sc_2CO	C
**$Sc_5C_4F_2$**	Sc_3C_4	Sc_4C_3	ScF_3
**$Sc_5C_4P_2$**	Sc_3C_4	ScP	
**$Sc_5C_4S_2$**	Sc_3C_4	ScS	
**$Sc_5C_4Cl_2$**	Sc_2CCl_2	Sc_3C_4	Sc_4C_3
**$Sc_5C_4As_2$**	Sc_3C_4	ScAs	
**$Sc_5C_4Se_2$**	Sc_3C_4	ScSe	
**$Sc_5C_4Br_2$**	Sc_3C_4	Sc_7CBr_{12}	Sc_4C_3
**$Sc_5C_4Sb_2$**	Sc_3C_4	ScSb	
**$Sc_5C_4Te_2$**	ScTe	Sc_3C_4	
**$Sc_5C_4I_2$**	Sc_3C_4	Sc_2CI_3	Sc_4C_3
**Sc_2N**	$Sc_{39}N_{34}$	Sc	
**Sc_2NO_2**	ScN	Sc_2O_3	N_2
**Sc_2NF_2**	$Sc_{39}N_{34}$	Sc	ScF_3
**Sc_2NP_2**	ScP	P	ScN
**Sc_2NS_2**	Sc_2S_3	N_2	ScN
**Sc_2NCl_2**	Sc_2NCl_2		
**Sc_2NAs_2**	ScAs	As	ScN
**Sc_2NSe_2**	Sc_2Se_3	N_2	ScN
**Sc_2NBr_2**	$Sc_{39}N_{34}$	$ScBr_3$	Sc
**Sc_2NSb_2**	ScSb	Sb	ScN
**Sc_2NTe_2**	Sc_2Te_3	Te	ScN
**Sc_2NI_2**	$Sc_{39}N_{34}$	$Sc_{12}I_{25}$	ScN
**Sc_3N_2**	$Sc_{39}N_{34}$	Sc	
**$Sc_3N_2O_2$**	ScN	Sc_2O_3	N_2
**$Sc_3N_2F_2$**	$Sc_{39}N_{34}$	Sc	ScF_3
**$Sc_3N_2P_2$**	ScP	P	ScN
**$Sc_3N_2S_2$**	Sc_2S_3	N_2	ScN
**$Sc_3N_2Cl_2$**	Sc_2NCl_2	ScN	
**$Sc_3N_2As_2$**	ScAs	As	ScN
**$Sc_3N_2Se_2$**	Sc_2Se_3	N_2	ScN

**$Sc_3N_2Br_2$**	$Sc_{39}N_{34}$	$ScBr_3$	Sc
**$Sc_3N_2Sb_2$**	ScSb	Sb	ScN
**$Sc_3N_2Te_2$**	Sc_2Te_3	Te	ScN
**$Sc_3N_2I_2$**	$Sc_{39}N_{34}$	$Sc_{12}I_{25}$	ScN
**Sc_4N_3**	$Sc_{39}N_{34}$	Sc	
**$Sc_4N_3O_2$**	ScN	Sc_2O_3	N_2
**$Sc_4N_3F_2$**	$Sc_{39}N_{34}$	ScN	ScF_3
**$Sc_4N_3P_2$**	ScP	P	ScN
**$Sc_4N_3S_2$**	Sc_2S_3	N_2	ScN
**$Sc_4N_3Cl_2$**	Sc_2NCl_2	ScN	
**$Sc_4N_3As_2$**	ScAs	As	ScN
**$Sc_4N_3Se_2$**	Sc_2Se_3	N_2	ScN
**$Sc_4N_3Br_2$**	$Sc_{39}N_{34}$	$ScBr_3$	ScN
**$Sc_4N_3Sb_2$**	ScSb	Sb	ScN
**$Sc_4N_3Te_2$**	Sc_2Te_3	Te	ScN
**$Sc_4N_3I_2$**	$Sc_{39}N_{34}$	$Sc_{12}I_{25}$	ScN
**Sc_5N_4**	$Sc_{39}N_{34}$	Sc	
**$Sc_5N_4O_2$**	ScN	Sc_2O_3	N_2
**$Sc_5N_4F_2$**	$Sc_{39}N_{34}$	ScN	ScF_3
**$Sc_5N_4P_2$**	ScP	P	ScN
**$Sc_5N_4S_2$**	Sc_2S_3	N_2	ScN
**$Sc_5N_4Cl_2$**	Sc_2NCl_2	ScN	
**$Sc_5N_4As_2$**	ScAs	As	ScN
**$Sc_5N_4Se_2$**	Sc_2Se_3	N_2	ScN
**$Sc_5N_4Br_2$**	$Sc_{39}N_{34}$	$ScBr_3$	ScN
**$Sc_5N_4Sb_2$**	ScSb	Sb	ScN
**$Sc_5N_4Te_2$**	Sc_2Te_3	Te	ScN
**$Sc_5N_4I_2$**	$Sc_{39}N_{34}$	$Sc_{12}I_{25}$	ScN
**Ti_2C**	Ti_2C		
**Ti_2CO_2**	TiO_2	TiC	
**Ti_2CF_2**	Ti_8C_5	TiF_3	TiC
**Ti_2CP_2**	TiP	C	
**Ti_2CS_2**	Ti_2S_3	Ti_2CS	C
**Ti_2CCl_2**	Ti_2CCl_2		
**Ti_2CAs_2**	TiAs	C	
**Ti_2CSe_2**	Ti_3Se_4	TiC	C
**Ti_2CBr_2**	$TiBr_3$	Ti_8C_5	TiC
**Ti_2CSb_2**	$TiSb_2$	TiC	
**Ti_2CTe_2**	$TiTe_2$	TiC	
**Ti_2CI_2**	Ti_8C_5	TiC	TiI_3
**Ti_3C_2**	Ti_8C_5	TiC	

**$Ti_3C_2O_2$**	TiO_2	TiC	
**$Ti_3C_2F_2$**	Ti_8C_5	TiF_3	TiC
**$Ti_3C_2P_2$**	TiP	C	TiC
**$Ti_3C_2S_2$**	Ti_2S_3	Ti_2CS	C
**$Ti_3C_2Cl_2$**	Ti_2CCl_2	TiC	
**$Ti_3C_2As_2$**	TiAs	C	TiC
**$Ti_3C_2Se_2$**	Ti_3Se_4	TiC	C
**$Ti_3C_2Br_2$**	$TiBr_3$	Ti_8C_5	TiC
**$Ti_3C_2Sb_2$**	$TiSb_2$	TiC	
**$Ti_3C_2Te_2$**	$TiTe_2$	TiC	
**$Ti_3C_2I_2$**	Ti_8C_5	TiC	TiI_3
**Ti_4C_3**	Ti_8C_5	TiC	
**$Ti_4C_3O_2$**	TiO_2	TiC	
**$Ti_4C_3F_2$**	Ti_8C_5	TiF_3	TiC
**$Ti_4C_3P_2$**	TiP	C	TiC
**$Ti_4C_3S_2$**	Ti_2CS	C	
**$Ti_4C_3Cl_2$**	Ti_2CCl_2	TiC	
**$Ti_4C_3As_2$**	TiAs	C	TiC
**$Ti_4C_3Se_2$**	Ti_3Se_4	TiC	C
**$Ti_4C_3Br_2$**	$TiBr_3$	Ti_8C_5	TiC
**$Ti_4C_3Sb_2$**	$TiSb_2$	TiC	
**$Ti_4C_3Te_2$**	$TiTe_2$	TiC	
**$Ti_4C_3I_2$**	Ti_8C_5	TiC	TiI_3
**Ti_5C_4**	Ti_8C_5	TiC	
**$Ti_5C_4O_2$**	TiO_2	TiC	
**$Ti_5C_4F_2$**	Ti_8C_5	TiF_3	TiC
**$Ti_5C_4P_2$**	TiP	C	TiC
**$Ti_5C_4S_2$**	Ti_2CS	TiC	C
**$Ti_5C_4Cl_2$**	Ti_2CCl_2	TiC	
**$Ti_5C_4As_2$**	TiAs	C	TiC
**$Ti_5C_4Se_2$**	Ti_3Se_4	TiC	C
**$Ti_5C_4Br_2$**	$TiBr_3$	Ti_8C_5	TiC
**$Ti_5C_4Sb_2$**	$TiSb_2$	TiC	
**$Ti_5C_4Te_2$**	$TiTe_2$	TiC	
**$Ti_5C_4I_2$**	Ti_8C_5	TiC	TiI_3
**Ti_2N**	Ti_2N		
**Ti_2NO_2**	TiN	TiO_2	
**Ti_2NF_2**	Ti_2N	TiF_3	TiN
**Ti_2NP_2**	TiP_2	TiN	
**Ti_2NS_2**	TiS_2	TiN	
**Ti_2NCl_2**	$TiCl_3$	Ti_2N	TiN

**Ti_2NAs_2**	$TiAs_2$	TiN	
**Ti_2NSe_2**	$TiSe_2$	TiN	
**Ti_2NBr_2**	$TiBr_3$	Ti_2N	TiN
**Ti_2NSb_2**	$TiSb_2$	TiN	
**Ti_2NTe_2**	$TiTe_2$	TiN	
**Ti_2NI_2**	TiI_3	Ti_2N	TiN
**Ti_3N_2**	Ti_2N	TiN	
**$Ti_3N_2O_2$**	TiN	TiO_2	
**$Ti_3N_2F_2$**	Ti_2N	TiF_3	TiN
**$Ti_3N_2P_2$**	TiP_2	TiN	
**$Ti_3N_2S_2$**	TiS_2	TiN	
**$Ti_3N_2Cl_2$**	$TiCl_3$	Ti_2N	TiN
**$Ti_3N_2As_2$**	$TiAs_2$	TiN	
**$Ti_3N_2Se_2$**	$TiSe_2$	TiN	
**$Ti_3N_2Br_2$**	$TiBr_3$	Ti_2N	TiN
**$Ti_3N_2Sb_2$**	$TiSb_2$	TiN	
**$Ti_3N_2Te_2$**	$TiTe_2$	TiN	
**$Ti_3N_2I_2$**	TiI_3	Ti_2N	TiN
**Ti_4N_3**	Ti_2N	TiN	
**$Ti_4N_3O_2$**	TiN	TiO_2	
**$Ti_4N_3F_2$**	Ti_2N	TiF_3	TiN
**$Ti_4N_3P_2$**	TiP_2	TiN	
**$Ti_4N_3S_2$**	TiS_2	TiN	
**$Ti_4N_3Cl_2$**	$TiCl_3$	Ti_2N	TiN
**$Ti_4N_3As_2$**	$TiAs_2$	TiN	
**$Ti_4N_3Se_2$**	$TiSe_2$	TiN	
**$Ti_4N_3Br_2$**	$TiBr_3$	Ti_2N	TiN
**$Ti_4N_3Sb_2$**	$TiSb_2$	TiN	
**$Ti_4N_3Te_2$**	$TiTe_2$	TiN	
**$Ti_4N_3I_2$**	TiI_3	Ti_2N	TiN
**Ti_5N_4**	Ti_2N	TiN	
**$Ti_5N_4O_2$**	TiN	TiO_2	
**$Ti_5N_4F_2$**	Ti_2N	TiF_3	TiN
**$Ti_5N_4P_2$**	TiP_2	TiN	
**$Ti_5N_4S_2$**	TiS_2	TiN	
**$Ti_5N_4Cl_2$**	$TiCl_3$	Ti_2N	TiN
**$Ti_5N_4As_2$**	$TiAs_2$	TiN	
**$Ti_5N_4Se_2$**	$TiSe_2$	TiN	
**$Ti_5N_4Br_2$**	$TiBr_3$	Ti_2N	TiN
**$Ti_5N_4Sb_2$**	$TiSb_2$	TiN	
**$Ti_5N_4Te_2$**	$TiTe_2$	TiN	

**$Ti_5N_4I_2$**	TiI_3	Ti_2N	TiN
**V_2C**	V_2C		
**V_2CO_2**	V_2O_3	V_6C_5	C
**V_2CF_2**	V_6C_5	C	VF_2
**V_2CP_2**	VP	C	
**V_2CS_2**	V_3S_4	V_6C_5	C
**V_2CCl_2**	VCl_3	V_6C_5	VCl_2
**V_2CAs_2**	VAs_2	V_2AsC	C
**V_2CSe_2**	V_5Se_8	V_6C_5	C
**V_2CBr_2**	V_6C_5	C	VBr_2
**V_2CSb_2**	VSb_2	V_6C_5	Sb
**V_2CTe_2**	V_6C_5	VTe_2	C
**V_2CI_2**	V_6C_5	C	VI_2
**V_3C_2**	V_2C	V_6C_5	
**$V_3C_2O_2$**	V_2O_3	V_6C_5	C
**$V_3C_2F_2$**	V_6C_5	C	VF_2
**$V_3C_2P_2$**	V_2PC	VP	C
**$V_3C_2S_2$**	V_3S_4	V_6C_5	C
**$V_3C_2Cl_2$**	VCl_3	V_6C_5	C
**$V_3C_2As_2$**	VAs_2	V_2AsC	C
**$V_3C_2Se_2$**	V_5Se_8	V_6C_5	C
**$V_3C_2Br_2$**	V_6C_5	C	VBr_2
**$V_3C_2Sb_2$**	VSb_2	V_6C_5	Sb
**$V_3C_2Te_2$**	V_6C_5	VTe_2	C
**$V_3C_2I_2$**	V_6C_5	C	VI_2
**V_4C_3**	V_2C	V_6C_5	
**$V_4C_3O_2$**	V_2O_3	V_6C_5	C
**$V_4C_3F_2$**	V_6C_5	C	VF_2
**$V_4C_3P_2$**	V_2PC	C	
**$V_4C_3S_2$**	V_3S_4	V_6C_5	C
**$V_4C_3Cl_2$**	VCl_3	V_6C_5	C
**$V_4C_3As_2$**	V_2AsC	C	
**$V_4C_3Se_2$**	V_5Se_8	V_6C_5	C
**$V_4C_3Br_2$**	V_6C_5	C	VBr_2
**$V_4C_3Sb_2$**	VSb_2	V_6C_5	Sb
**$V_4C_3Te_2$**	V_6C_5	VTe_2	C
**$V_4C_3I_2$**	V_6C_5	C	VI_2
**V_5C_4**	V_2C	V_6C_5	
**$V_5C_4O_2$**	V_2O_3	V_6C_5	C
**$V_5C_4F_2$**	V_6C_5	C	VF_2
**$V_5C_4P_2$**	V_2PC	V_6C_5	C

**$V_5C_4S_2$**	V_3S_4	V_6C_5	C
**$V_5C_4Cl_2$**	VCl_3	V_6C_5	C
**$V_5C_4As_2$**	V_6C_5	V_2AsC	C
**$V_5C_4Se_2$**	V_5Se_8	V_6C_5	C
**$V_5C_4Br_2$**	V_6C_5	C	VBr_2
**$V_5C_4Sb_2$**	VSb_2	V_6C_5	Sb
**$V_5C_4Te_2$**	V_6C_5	VTe_2	C
**$V_5C_4I_2$**	V_6C_5	C	VI_2
**V_2N**	V_2N		
**V_2NO_2**	V_2O_3	VN	N_2
**V_2NF_2**	VN	VF_2	
**V_2NP_2**	VP_2	VN	
**V_2NS_2**	VS_2	VN	
**V_2NCl_2**	VCl_3	V_2N	VN
**V_2NAs_2**	VAs_2	VN	
**V_2NSe_2**	VSe_2	VN	
**V_2NBr_2**	VBr_2	VN	
**V_2NSb_2**	V_2N	Sb	
**V_2NTe_2**	VTe_2	VN	
**V_2NI_2**	VI_2	VN	
**V_3N_2**	V_2N	VN	
**$V_3N_2O_2$**	V_2O_3	VN	N_2
**$V_3N_2F_2$**	VN	VF_2	
**$V_3N_2P_2$**	VP_2	VN	
**$V_3N_2S_2$**	VS_2	VN	
**$V_3N_2Cl_2$**	VCl_3	V_2N	VN
**$V_3N_2As_2$**	VAs_2	VN	
**$V_3N_2Se_2$**	VSe_2	VN	
**$V_3N_2Br_2$**	VBr_2	VN	
**$V_3N_2Sb_2$**	V_2N	Sb	VN
**$V_3N_2Te_2$**	VTe_2	VN	
**$V_3N_2I_2$**	VI_2	VN	
**V_4N_3**	V_2N	VN	
**$V_4N_3O_2$**	V_2O_3	VN	N_2
**$V_4N_3F_2$**	VN	VF_2	
**$V_4N_3P_2$**	VP_2	VN	
**$V_4N_3S_2$**	VS_2	VN	
**$V_4N_3Cl_2$**	VCl_3	V_2N	VN
**$V_4N_3As_2$**	VAs_2	VN	
**$V_4N_3Se_2$**	VSe_2	VN	
**$V_4N_3Br_2$**	VBr_2	VN	

**$V_4N_3Sb_2$**	V_2N	Sb	VN
**$V_4N_3Te_2$**	VTe_2	VN	
**$V_4N_3I_2$**	VI_2	VN	
**V_5N_4**	V_2N	VN	
**$V_5N_4O_2$**	V_2O_3	VN	N_2
**$V_5N_4F_2$**	VN	VF_2	
**$V_5N_4P_2$**	VP_2	VN	
**$V_5N_4S_2$**	VS_2	VN	
**$V_5N_4Cl_2$**	VCl_3	V_2N	VN
**$V_5N_4As_2$**	VAs_2	VN	
**$V_5N_4Se_2$**	VSe_2	VN	
**$V_5N_4Br_2$**	VBr_2	VN	
**$V_5N_4Sb_2$**	V_2N	Sb	VN
**$V_5N_4Te_2$**	VTe_2	VN	
**$V_5N_4I_2$**	VI_2	VN	
**Cr_2C**	Cr_7C_3	Cr_3C_2	
**Cr_2CO_2**	Cr_2O_3	Cr_3C_2	C
**Cr_2CF_2**	Cr_3C_2	C	CrF_2
**Cr_2CP_2**	CrP	C	
**Cr_2CS_2**	Cr_2S_3	Cr_3C_2	C
**Cr_2CCl_2**	Cr_3C_2	$CrCl_3$	C
**Cr_2CAs_2**	CrAs	C	
**Cr_2CSe_2**	Cr_3C_2	Cr_2Se_3	C
**Cr_2CBr_2**	$CrBr_3$	Cr_3C_2	C
**Cr_2CSb_2**	$CrSb_2$	Cr_3C_2	Sb
**Cr_2CTe_2**	Cr_5Te_8	Cr_3C_2	C
**Cr_2CI_2**	CrI_3	Cr_3C_2	C
**Cr_3C_2**	Cr_3C_2		
**$Cr_3C_2O_2$**	Cr_2O_3	Cr_3C_2	C
**$Cr_3C_2F_2$**	Cr_3C_2	C	CrF_2
**$Cr_3C_2P_2$**	Cr_3C_2	CrP	C
**$Cr_3C_2S_2$**	Cr_2S_3	Cr_3C_2	C
**$Cr_3C_2Cl_2$**	Cr_3C_2	$CrCl_3$	C
**$Cr_3C_2As_2$**	Cr_3C_2	CrAs	C
**$Cr_3C_2Se_2$**	Cr_3C_2	Cr_2Se_3	C
**$Cr_3C_2Br_2$**	$CrBr_3$	Cr_3C_2	C
**$Cr_3C_2Sb_2$**	Cr_3C_2	Sb	
**$Cr_3C_2Te_2$**	Cr_5Te_8	Cr_3C_2	C
**$Cr_3C_2I_2$**	CrI_3	Cr_3C_2	C
**Cr_4C_3**	Cr_3C_2	C	
**$Cr_4C_3O_2$**	Cr_2O_3	Cr_3C_2	C

**$Cr_4C_3F_2$**	Cr_3C_2	C	CrF_2
**$Cr_4C_3P_2$**	Cr_3C_2	CrP	C
**$Cr_4C_3S_2$**	Cr_2S_3	Cr_3C_2	C
**$Cr_4C_3Cl_2$**	Cr_3C_2	$CrCl_3$	C
**$Cr_4C_3As_2$**	Cr_3C_2	CrAs	C
**$Cr_4C_3Se_2$**	Cr_3C_2	Cr_2Se_3	C
**$Cr_4C_3Br_2$**	$CrBr_3$	Cr_3C_2	C
**$Cr_4C_3Sb_2$**	Cr_3C_2	C	Sb
**$Cr_4C_3Te_2$**	Cr_5Te_8	Cr_3C_2	C
**$Cr_4C_3I_2$**	CrI_3	Cr_3C_2	C
**Cr_5C_4**	Cr_3C_2	C	
**$Cr_5C_4O_2$**	Cr_2O_3	Cr_3C_2	C
**$Cr_5C_4F_2$**	Cr_3C_2	C	CrF_2
**$Cr_5C_4P_2$**	Cr_3C_2	CrP	C
**$Cr_5C_4S_2$**	Cr_2S_3	Cr_3C_2	C
**$Cr_5C_4Cl_2$**	Cr_3C_2	$CrCl_3$	C
**$Cr_5C_4As_2$**	Cr_3C_2	CrAs	C
**$Cr_5C_4Se_2$**	Cr_3C_2	Cr_2Se_3	C
**$Cr_5C_4Br_2$**	$CrBr_3$	Cr_3C_2	C
**$Cr_5C_4Sb_2$**	Cr_3C_2	C	Sb
**$Cr_5C_4Te_2$**	Cr_5Te_8	Cr_3C_2	C
**$Cr_5C_4I_2$**	CrI_3	Cr_3C_2	C
**Cr_2N**	Cr_2N		
**Cr_2NO_2**	CrN	Cr_2O_3	N_2
**Cr_2NF_2**	CrN	CrF_2	
**Cr_2NP_2**	CrP	P_3N_5	CrN
**Cr_2NS_2**	Cr_2S_3	N_2	CrN
**Cr_2NCl_2**	Cr_2N	CrN	$CrCl_3$
**Cr_2NAs_2**	CrAs	As	CrN
**Cr_2NSe_2**	$CrSe_2$	CrN	
**Cr_2NBr_2**	Cr_2N	$CrBr_3$	CrN
**Cr_2NSb_2**	Cr_2N	Sb	
**Cr_2NTe_2**	$CrTe_2$	CrN	
**Cr_2NI_2**	Cr_2N	CrI_3	CrN
**Cr_3N_2**	Cr_2N	CrN	
**$Cr_3N_2O_2$**	CrN	Cr_2O_3	N_2
**$Cr_3N_2F_2$**	CrN	CrF_2	
**$Cr_3N_2P_2$**	CrP	P_3N_5	CrN
**$Cr_3N_2S_2$**	Cr_2S_3	N_2	CrN
**$Cr_3N_2Cl_2$**	Cr_2N	CrN	$CrCl_3$
**$Cr_3N_2As_2$**	CrAs	As	CrN

**$Cr_3N_2Se_2$**	$CrSe_2$	CrN	
**$Cr_3N_2Br_2$**	Cr_2N	$CrBr_3$	CrN
**$Cr_3N_2Sb_2$**	Cr_2N	Sb	CrN
**$Cr_3N_2Te_2$**	$CrTe_2$	CrN	
**$Cr_3N_2I_2$**	Cr_2N	CrI_3	CrN
**Cr_4N_3**	Cr_2N	CrN	
**$Cr_4N_3O_2$**	CrN	Cr_2O_3	N_2
**$Cr_4N_3F_2$**	CrN	CrF_2	
**$Cr_4N_3P_2$**	CrP	P_3N_5	CrN
**$Cr_4N_3S_2$**	Cr_2S_3	N_2	CrN
**$Cr_4N_3Cl_2$**	Cr_2N	CrN	$CrCl_3$
**$Cr_4N_3As_2$**	CrAs	As	CrN
**$Cr_4N_3Se_2$**	$CrSe_2$	CrN	
**$Cr_4N_3Br_2$**	Cr_2N	$CrBr_3$	CrN
**$Cr_4N_3Sb_2$**	Cr_2N	Sb	CrN
**$Cr_4N_3Te_2$**	$CrTe_2$	CrN	
**$Cr_4N_3I_2$**	Cr_2N	CrI_3	CrN
**Cr_5N_4**	Cr_2N	CrN	
**$Cr_5N_4O_2$**	CrN	Cr_2O_3	N_2
**$Cr_5N_4F_2$**	CrN	CrF_2	
**$Cr_5N_4P_2$**	CrP	P_3N_5	CrN
**$Cr_5N_4S_2$**	Cr_2S_3	N_2	CrN
**$Cr_5N_4Cl_2$**	Cr_2N	CrN	$CrCl_3$
**$Cr_5N_4As_2$**	CrAs	As	CrN
**$Cr_5N_4Se_2$**	$CrSe_2$	CrN	
**$Cr_5N_4Br_2$**	Cr_2N	$CrBr_3$	CrN
**$Cr_5N_4Sb_2$**	Cr_2N	Sb	CrN
**$Cr_5N_4Te_2$**	$CrTe_2$	CrN	
**$Cr_5N_4I_2$**	Cr_2N	CrI_3	CrN
**Mn_2C**	$Mn_{23}C_6$	C	
**Mn_2CO_2**	C	MnO	
**Mn_2CF_2**	$Mn_{23}C_6$	C	MnF_2
**Mn_2CP_2**	MnP	C	
**Mn_2CS_2**	C	MnS	
**Mn_2CCl_2**	$Mn_{23}C_6$	C	$MnCl_2$
**Mn_2CAs_2**	MnAs	C	
**Mn_2CSe_2**	C	MnSe	
**Mn_2CBr_2**	$Mn_{23}C_6$	C	$MnBr_2$
**Mn_2CSb_2**	MnSb	C	
**Mn_2CTe_2**	$Mn_{23}C_6$	$MnTe_2$	C
**Mn_2CI_2**	$Mn_{23}C_6$	C	MnI_2

**Mn_3C_2**	$Mn_{23}C_6$	C	
**$Mn_3C_2O_2$**	$Mn_{23}C_6$	C	MnO
**$Mn_3C_2F_2$**	$Mn_{23}C_6$	C	MnF_2
**$Mn_3C_2P_2$**	Mn_2P	MnP	C
**$Mn_3C_2S_2$**	$Mn_{23}C_6$	C	MnS
**$Mn_3C_2Cl_2$**	$Mn_{23}C_6$	C	$MnCl_2$
**$Mn_3C_2As_2$**	$Mn_{23}C_6$	MnAs	C
**$Mn_3C_2Se_2$**	$Mn_{23}C_6$	C	MnSe
**$Mn_3C_2Br_2$**	$Mn_{23}C_6$	C	$MnBr_2$
**$Mn_3C_2Sb_2$**	$Mn_{23}C_6$	MnSb	C
**$Mn_3C_2Te_2$**	$Mn_{23}C_6$	$MnTe_2$	C
**$Mn_3C_2I_2$**	$Mn_{23}C_6$	C	MnI_2
**Mn_4C_3**	$Mn_{23}C_6$	C	
**$Mn_4C_3O_2$**	$Mn_{23}C_6$	C	MnO
**$Mn_4C_3F_2$**	$Mn_{23}C_6$	C	MnF_2
**$Mn_4C_3P_2$**	Mn_2P	C	
**$Mn_4C_3S_2$**	$Mn_{23}C_6$	C	MnS
**$Mn_4C_3Cl_2$**	$Mn_{23}C_6$	C	$MnCl_2$
**$Mn_4C_3As_2$**	$Mn_{23}C_6$	MnAs	C
**$Mn_4C_3Se_2$**	$Mn_{23}C_6$	C	MnSe
**$Mn_4C_3Br_2$**	$Mn_{23}C_6$	C	$MnBr_2$
**$Mn_4C_3Sb_2$**	$Mn_{23}C_6$	MnSb	C
**$Mn_4C_3Te_2$**	$Mn_{23}C_6$	$MnTe_2$	C
**$Mn_4C_3I_2$**	$Mn_{23}C_6$	C	MnI_2
**Mn_5C_4**	$Mn_{23}C_6$	C	
**$Mn_5C_4O_2$**	$Mn_{23}C_6$	C	MnO
**$Mn_5C_4F_2$**	$Mn_{23}C_6$	C	MnF_2
**$Mn_5C_4P_2$**	Mn_2P	$Mn_{23}C_6$	C
**$Mn_5C_4S_2$**	$Mn_{23}C_6$	C	MnS
**$Mn_5C_4Cl_2$**	$Mn_{23}C_6$	C	$MnCl_2$
**$Mn_5C_4As_2$**	$Mn_{23}C_6$	MnAs	C
**$Mn_5C_4Se_2$**	$Mn_{23}C_6$	C	MnSe
**$Mn_5C_4Br_2$**	$Mn_{23}C_6$	C	$MnBr_2$
**$Mn_5C_4Sb_2$**	$Mn_{23}C_6$	MnSb	C
**$Mn_5C_4Te_2$**	$Mn_{23}C_6$	$MnTe_2$	C
**$Mn_5C_4I_2$**	$Mn_{23}C_6$	C	MnI_2
**Mn_2N**	Mn_2N		
**Mn_2NO_2**	N_2	MnO	
**Mn_2NF_2**	MnN	MnF_2	
**Mn_2NP_2**	MnP	P_3N_5	MnN
**Mn_2NS_2**	MnS_2	MnN	

**Mn_2NCl_2**	MnN	$MnCl_2$	
**Mn_2NAs_2**	MnAs	As	MnN
**Mn_2NSe_2**	$MnSe_2$	MnN	
**Mn_2NBr_2**	$MnBr_2$	MnN	
**Mn_2NSb_2**	Mn_2N	Sb	
**Mn_2NTe_2**	$MnTe_2$	MnN	
**Mn_2NI_2**	MnI_2	MnN	
**Mn_3N_2**	Mn_2N	MnN	
**$Mn_3N_2O_2$**	MnN	N_2	MnO
**$Mn_3N_2F_2$**	MnN	MnF_2	
**$Mn_3N_2P_2$**	MnP	P_3N_5	MnN
**$Mn_3N_2S_2$**	MnS_2	MnN	
**$Mn_3N_2Cl_2$**	MnN	$MnCl_2$	
**$Mn_3N_2As_2$**	MnAs	As	MnN
**$Mn_3N_2Se_2$**	$MnSe_2$	MnN	
**$Mn_3N_2Br_2$**	$MnBr_2$	MnN	
**$Mn_3N_2Sb_2$**	Mn_2N	Sb	MnN
**$Mn_3N_2Te_2$**	$MnTe_2$	MnN	
**$Mn_3N_2I_2$**	MnI_2	MnN	
**Mn_4N_3**	Mn_2N	MnN	
**$Mn_4N_3O_2$**	MnN	N_2	MnO
**$Mn_4N_3F_2$**	MnN	MnF_2	
**$Mn_4N_3P_2$**	MnP	P_3N_5	MnN
**$Mn_4N_3S_2$**	MnS_2	MnN	
**$Mn_4N_3Cl_2$**	MnN	$MnCl_2$	
**$Mn_4N_3As_2$**	MnAs	As	MnN
**$Mn_4N_3Se_2$**	$MnSe_2$	MnN	
**$Mn_4N_3Br_2$**	$MnBr_2$	MnN	
**$Mn_4N_3Sb_2$**	Mn_2N	Sb	MnN
**$Mn_4N_3Te_2$**	$MnTe_2$	MnN	
**$Mn_4N_3I_2$**	MnI_2	MnN	
**Mn_5N_4**	Mn_2N	MnN	
**$Mn_5N_4O_2$**	MnN	N_2	MnO
**$Mn_5N_4F_2$**	MnN	MnF_2	
**$Mn_5N_4P_2$**	MnP	P_3N_5	MnN
**$Mn_5N_4S_2$**	MnS_2	MnN	
**$Mn_5N_4Cl_2$**	MnN	$MnCl_2$	
**$Mn_5N_4As_2$**	MnAs	As	MnN
**$Mn_5N_4Se_2$**	$MnSe_2$	MnN	
**$Mn_5N_4Br_2$**	$MnBr_2$	MnN	
**$Mn_5N_4Sb_2$**	Mn_2N	Sb	MnN

**$Mn_5N_4Te_2$**	$MnTe_2$	MnN	
**$Mn_5N_4I_2$**	MnI_2	MnN	
**Y_2C**	Y_2C		
**Y_2CO_2**	Y_3C_4	Y_4C_7	Y_2O_3
**Y_2CF_2**	Y_4C_5	Y_2C	YF_3
**Y_2CP_2**	YP	C	
**Y_2CS_2**	C	YS	
**Y_2CCl_2**	YCCl	Y_2C	YCl_3
**Y_2CAs_2**	YAs	C	
**Y_2CSe_2**	C	YSe	
**Y_2CBr_2**	YCBr	Y_3CBr_3	
**Y_2CSb_2**	YSb	C	
**Y_2CTe_2**	YTe	Y_4C_7	Y_2Te_3
**Y_2CI_2**	Y_2CI_2		
**Y_3C_2**	Y_2C	Y_4C_5	
**$Y_3C_2O_2$**	Y_2C	Y_2O_3	Y_4C_5
**$Y_3C_2F_2$**	Y_4C_5	Y_2C	YF_3
**$Y_3C_2P_2$**	Y_4C_7	YP	C
**$Y_3C_2S_2$**	Y_4C_7	C	YS
**$Y_3C_2Cl_2$**	YCCl	Y_2C	YCl_3
**$Y_3C_2As_2$**	Y_4C_7	YAs	C
**$Y_3C_2Se_2$**	Y_4C_7	C	YSe
**$Y_3C_2Br_2$**	YCBr	Y_3CBr_3	Y_4C_5
**$Y_3C_2Sb_2$**	Y_4C_7	YSb	C
**$Y_3C_2Te_2$**	YTe	Y_4C_7	Y_2Te_3
**$Y_3C_2I_2$**	Y_2CI_2	Y_2C	Y_4C_5
**Y_4C_3**	Y_2C	Y_4C_5	
**$Y_4C_3O_2$**	Y_2C	Y_2O_3	Y_4C_5
**$Y_4C_3F_2$**	Y_4C_5	Y_2C	YF_3
**$Y_4C_3P_2$**	Y_3C_4	Y_4C_7	YP
**$Y_4C_3S_2$**	Y_3C_4	Y_4C_7	YS
**$Y_4C_3Cl_2$**	YCCl	Y_2C	
**$Y_4C_3As_2$**	Y_3C_4	Y_4C_7	YAs
**$Y_4C_3Se_2$**	Y_3C_4	Y_4C_7	YSe
**$Y_4C_3Br_2$**	Y_2C	Y_3CBr_3	Y_4C_5
**$Y_4C_3Sb_2$**	Y_3C_4	Y_4C_7	YSb
**$Y_4C_3Te_2$**	Y_3C_4	YTe	Y_4C_7
**$Y_4C_3I_2$**	Y_2CI_2	Y_2C	Y_4C_5
**Y_5C_4**	Y_2C	Y_4C_5	
**$Y_5C_4O_2$**	Y_2C	Y_2O_3	Y_4C_5
**$Y_5C_4F_2$**	Y_4C_5	Y_2C	YF_3

**$Y_5C_4P_2$**	Y_3C_4	YP	
**$Y_5C_4S_2$**	Y_3C_4	YS	
**$Y_5C_4Cl_2$**	YCCl	Y_2C	Y_4C_5
**$Y_5C_4As_2$**	Y_3C_4	YAs	
**$Y_5C_4Se_2$**	Y_3C_4	YSe	
**$Y_5C_4Br_2$**	Y_2C	Y_3CBr_3	Y_4C_5
**$Y_5C_4Sb_2$**	Y_3C_4	YSb	
**$Y_5C_4Te_2$**	Y_3C_4	YTe	
**$Y_5C_4I_2$**	Y_2CI_2	Y_2C	Y_4C_5
**Y_2N**	YN	Y	
**Y_2NO_2**	YN	Y_2O_3	N_2
**Y_2NF_2**	Y	YN	YF_3
**Y_2NP_2**	YP	P_3N_5	YN
**Y_2NS_2**	$Y_4S_3N_2$	Y_2S_3	N_2
**Y_2NCl_2**	Y_2NCl_3	YN	Y
**Y_2NAs_2**	YAs	As	YN
**Y_2NSe_2**	$Y_4Se_3N_2$	Y_2Se_3	N_2
**Y_2NBr_2**	Y_2Br_3	YBr_3	YN
**Y_2NSb_2**	YSb	Sb	YN
**Y_2NTe_2**	Y_3Te_3N	YTe_3	YN
**Y_2NI_2**	YI_3	Y	YN
**Y_3N_2**	YN	Y	
**$Y_3N_2O_2$**	YN	Y_2O_3	N_2
**$Y_3N_2F_2$**	Y	YN	YF_3
**$Y_3N_2P_2$**	YP	P_3N_5	YN
**$Y_3N_2S_2$**	$Y_4S_3N_2$	N_2	YN
**$Y_3N_2Cl_2$**	Y_2NCl_3	YN	Y
**$Y_3N_2As_2$**	YAs	As	YN
**$Y_3N_2Se_2$**	$Y_4Se_3N_2$	N_2	YN
**$Y_3N_2Br_2$**	Y_2Br_3	YBr_3	YN
**$Y_3N_2Sb_2$**	YSb	Sb	YN
**$Y_3N_2Te_2$**	Y_3Te_3N	YTe_3	YN
**$Y_3N_2I_2$**	YI_3	Y	YN
**Y_4N_3**	YN	Y	
**$Y_4N_3O_2$**	YN	Y_2O_3	N_2
**$Y_4N_3F_2$**	Y	YN	YF_3
**$Y_4N_3P_2$**	YP	P_3N_5	YN
**$Y_4N_3S_2$**	$Y_4S_3N_2$	N_2	YN
**$Y_4N_3Cl_2$**	Y_2NCl_3	YN	Y
**$Y_4N_3As_2$**	YAs	As	YN
**$Y_4N_3Se_2$**	$Y_4Se_3N_2$	N_2	YN

**$Y_4N_3Br_2$**	Y_2Br_3	YBr_3	YN
**$Y_4N_3Sb_2$**	YSb	Sb	YN
**$Y_4N_3Te_2$**	Y_3Te_3N	YTe_3	YN
**$Y_4N_3I_2$**	YI_3	Y	YN
**Y_5N_4**	YN	Y	
**$Y_5N_4O_2$**	YN	Y_2O_3	N_2
**$Y_5N_4F_2$**	Y	YN	YF_3
**$Y_5N_4P_2$**	YP	P_3N_5	YN
**$Y_5N_4S_2$**	$Y_4S_3N_2$	N_2	YN
**$Y_5N_4Cl_2$**	Y_2NCl_3	YN	Y
**$Y_5N_4As_2$**	YAs	As	YN
**$Y_5N_4Se_2$**	$Y_4Se_3N_2$	N_2	YN
**$Y_5N_4Br_2$**	Y_2Br_3	YBr_3	YN
**$Y_5N_4Sb_2$**	YSb	Sb	YN
**$Y_5N_4Te_2$**	Y_3Te_3N	YTe_3	YN
**$Y_5N_4I_2$**	YI_3	Y	YN
**Zr_2C**	$Zr_{10}C_9$	Zr	
**Zr_2CO_2**	ZrC	ZrO_2	
**Zr_2CF_2**	$Zr_{10}C_9$	Zr	ZrF_4
**Zr_2CP_2**	ZrP	C	
**Zr_2CS_2**	Zr_2CS	ZrS_2	C
**Zr_2CCl_2**	Zr_6CCl_{14}	$Zr_{10}C_9$	ZrCl
**Zr_2CAs_2**	ZrAs	C	
**Zr_2CSe_2**	$ZrSe_2$	ZrC	
**Zr_2CBr_2**	$ZrBr_3$	$Zr_{10}C_9$	ZrBr
**Zr_2CSb_2**	$ZrSb_2$	ZrC	
**Zr_2CTe_2**	$ZrTe_2$	ZrC	
**Zr_2CI_2**	Zr_6CI_{14}	$Zr_{10}C_9$	Zr
**Zr_3C_2**	$Zr_{10}C_9$	Zr	
**$Zr_3C_2O_2$**	ZrC	ZrO_2	
**$Zr_3C_2F_2$**	$Zr_{10}C_9$	Zr	ZrF_4
**$Zr_3C_2P_2$**	ZrP	C	ZrC
**$Zr_3C_2S_2$**	Zr_2CS	ZrS_2	C
**$Zr_3C_2Cl_2$**	Zr_6CCl_{14}	$Zr_{10}C_9$	ZrCl
**$Zr_3C_2As_2$**	ZrAs	C	ZrC
**$Zr_3C_2Se_2$**	$ZrSe_2$	ZrC	
**$Zr_3C_2Br_2$**	$ZrBr_3$	$Zr_{10}C_9$	ZrBr
**$Zr_3C_2Sb_2$**	$ZrSb_2$	ZrC	
**$Zr_3C_2Te_2$**	$ZrTe_2$	ZrC	
**$Zr_3C_2I_2$**	Zr_6CI_{14}	$Zr_{10}C_9$	Zr
**Zr_4C_3**	$Zr_{10}C_9$	Zr	

**$Zr_4C_3O_2$**	ZrC	ZrO_2	
**$Zr_4C_3F_2$**	$Zr_{10}C_9$	Zr	ZrF_4
**$Zr_4C_3P_2$**	ZrP	C	ZrC
**$Zr_4C_3S_2$**	Zr_2CS	C	
**$Zr_4C_3Cl_2$**	Zr_6CCl_{14}	$Zr_{10}C_9$	ZrC
**$Zr_4C_3As_2$**	$ZrAs$	C	ZrC
**$Zr_4C_3Se_2$**	$ZrSe_2$	ZrC	
**$Zr_4C_3Br_2$**	$ZrBr_3$	$Zr_{10}C_9$	
**$Zr_4C_3Sb_2$**	$ZrSb_2$	ZrC	
**$Zr_4C_3Te_2$**	$ZrTe_2$	ZrC	
**$Zr_4C_3I_2$**	Zr_6CI_{14}	$Zr_{10}C_9$	ZrC
**Zr_5C_4**	$Zr_{10}C_9$	Zr	
**$Zr_5C_4O_2$**	ZrC	ZrO_2	
**$Zr_5C_4F_2$**	$Zr_{10}C_9$	Zr	ZrF_4
**$Zr_5C_4P_2$**	ZrP	C	ZrC
**$Zr_5C_4S_2$**	Zr_2CS	ZrC	C
**$Zr_5C_4Cl_2$**	Zr_6CCl_{14}	$Zr_{10}C_9$	ZrC
**$Zr_5C_4As_2$**	$ZrAs$	C	ZrC
**$Zr_5C_4Se_2$**	$ZrSe_2$	ZrC	
**$Zr_5C_4Br_2$**	$ZrBr_3$	$Zr_{10}C_9$	ZrC
**$Zr_5C_4Sb_2$**	$ZrSb_2$	ZrC	
**$Zr_5C_4Te_2$**	$ZrTe_2$	ZrC	
**$Zr_5C_4I_2$**	Zr_6CI_{14}	$Zr_{10}C_9$	ZrC
**Zr_2N**	Zr_2N		
**Zr_2NO_2**	ZrN	ZrO_2	
**Zr_2NF_2**	Zr_2N	ZrN	ZrF_4
**Zr_2NP_2**	ZrP_2	ZrN	
**Zr_2NS_2**	ZrS_2	Zr_3S_4	Zr_2SN_2
**Zr_2NCl_2**	ZrN	Zr_6NCl_{15}	Zr_2N
**Zr_2NAs_2**	$ZrAs_2$	ZrN	
**Zr_2NSe_2**	$ZrSe_2$	ZrN	
**Zr_2NBr_2**	$ZrBr_3$	Zr_2N	ZrN
**Zr_2NSb_2**	$ZrSb_2$	ZrN	
**Zr_2NTe_2**	$ZrTe_2$	ZrN	
**Zr_2NI_2**	ZrI_2	ZrN	
**Zr_3N_2**	Zr_2N	ZrN	
**$Zr_3N_2O_2$**	ZrN	ZrO_2	
**$Zr_3N_2F_2$**	Zr_2N	ZrN	ZrF_4
**$Zr_3N_2P_2$**	ZrP_2	ZrN	
**$Zr_3N_2S_2$**	Zr_3S_4	Zr_2SN_2	ZrN
**$Zr_3N_2Cl_2$**	ZrN	Zr_6NCl_{15}	Zr_2N

**$Zr_3N_2As_2$**	$ZrAs_2$	ZrN	
**$Zr_3N_2Se_2$**	$ZrSe_2$	ZrN	
**$Zr_3N_2Br_2$**	$ZrBr_3$	Zr_2N	ZrN
**$Zr_3N_2Sb_2$**	$ZrSb_2$	ZrN	
**$Zr_3N_2Te_2$**	$ZrTe_2$	ZrN	
**$Zr_3N_2I_2$**	ZrI_2	ZrN	
**Zr_4N_3**	Zr_2N	ZrN	
**$Zr_4N_3O_2$**	ZrN	ZrO_2	
**$Zr_4N_3F_2$**	Zr_2N	ZrN	ZrF_4
**$Zr_4N_3P_2$**	ZrP_2	ZrN	
**$Zr_4N_3S_2$**	Zr_3S_4	Zr_2SN_2	ZrN
**$Zr_4N_3Cl_2$**	ZrN	Zr_6NCl_{15}	Zr_2N
**$Zr_4N_3As_2$**	$ZrAs_2$	ZrN	
**$Zr_4N_3Se_2$**	$ZrSe_2$	ZrN	
**$Zr_4N_3Br_2$**	$ZrBr_3$	Zr_2N	ZrN
**$Zr_4N_3Sb_2$**	$ZrSb_2$	ZrN	
**$Zr_4N_3Te_2$**	$ZrTe_2$	ZrN	
**$Zr_4N_3I_2$**	ZrI_2	ZrN	
**Zr_5N_4**	Zr_2N	ZrN	
**$Zr_5N_4O_2$**	ZrN	ZrO_2	
**$Zr_5N_4F_2$**	Zr_2N	ZrN	ZrF_4
**$Zr_5N_4P_2$**	ZrP_2	ZrN	
**$Zr_5N_4S_2$**	Zr_3S_4	Zr_2SN_2	ZrN
**$Zr_5N_4Cl_2$**	ZrN	Zr_6NCl_{15}	Zr_2N
**$Zr_5N_4As_2$**	$ZrAs_2$	ZrN	
**$Zr_5N_4Se_2$**	$ZrSe_2$	ZrN	
**$Zr_5N_4Br_2$**	$ZrBr_3$	Zr_2N	ZrN
**$Zr_5N_4Sb_2$**	$ZrSb_2$	ZrN	
**$Zr_5N_4Te_2$**	$ZrTe_2$	ZrN	
**$Zr_5N_4I_2$**	ZrI_2	ZrN	
**Nb_2C**	Nb_2C		
**Nb_2CO_2**	$Nb_{12}O_{29}$	Nb_6C_5	C
**Nb_2CF_2**	Nb_6C_5	Nb_2F_5	
**Nb_2CP_2**	NbP	C	
**Nb_2CS_2**	Nb_3S_4	Nb_6C_5	C
**Nb_2CCl_2**	Nb_3Cl_7	Nb_3Cl_8	Nb_6C_5
**Nb_2CAs_2**	NbAs	C	
**Nb_2CSe_2**	Nb_3Se_4	Nb_6C_5	C
**Nb_2CBr_2**	Nb_3Br_8	Nb_6C_5	Nb_2C
**Nb_2CSb_2**	Nb_6C_5	$NbSb_2$	C
**Nb_2CTe_2**	$NbTe_2$	Nb_6C_5	C

**Nb_2CI_2**	Nb_3I_8	Nb_6C_5	Nb_2C
**Nb_3C_2**	Nb_2C	Nb_6C_5	
**$Nb_3C_2O_2$**	$Nb_{12}O_{29}$	Nb_6C_5	C
**$Nb_3C_2F_2$**	Nb_6C_5	NbF_5	Nb_2F_5
**$Nb_3C_2P_2$**	Nb_2PC	NbP	C
**$Nb_3C_2S_2$**	Nb_3S_4	Nb_6C_5	C
**$Nb_3C_2Cl_2$**	Nb_3Cl_8	Nb_6C_5	C
**$Nb_3C_2As_2$**	NbAs	Nb_2AsC	C
**$Nb_3C_2Se_2$**	Nb_3Se_4	Nb_6C_5	C
**$Nb_3C_2Br_2$**	Nb_3Br_8	Nb_6C_5	C
**$Nb_3C_2Sb_2$**	Nb_6C_5	$NbSb_2$	C
**$Nb_3C_2Te_2$**	$NbTe_2$	Nb_6C_5	C
**$Nb_3C_2I_2$**	Nb_3I_8	Nb_6C_5	C
**Nb_4C_3**	Nb_2C	Nb_6C_5	
**$Nb_4C_3O_2$**	$Nb_{12}O_{29}$	Nb_6C_5	C
**$Nb_4C_3F_2$**	Nb_6C_5	NbF_5	
**$Nb_4C_3P_2$**	Nb_2PC	C	
**$Nb_4C_3S_2$**	Nb_3S_4	Nb_6C_5	C
**$Nb_4C_3Cl_2$**	Nb_3Cl_8	Nb_6C_5	C
**$Nb_4C_3As_2$**	Nb_2AsC	C	
**$Nb_4C_3Se_2$**	Nb_3Se_4	Nb_6C_5	C
**$Nb_4C_3Br_2$**	Nb_3Br_8	Nb_6C_5	C
**$Nb_4C_3Sb_2$**	Nb_6C_5	$NbSb_2$	C
**$Nb_4C_3Te_2$**	$NbTe_2$	Nb_6C_5	C
**$Nb_4C_3I_2$**	Nb_3I_8	Nb_6C_5	C
**Nb_5C_4**	Nb_2C	Nb_6C_5	
**$Nb_5C_4O_2$**	$Nb_{12}O_{29}$	Nb_6C_5	C
**$Nb_5C_4F_2$**	Nb_6C_5	NbF_5	C
**$Nb_5C_4P_2$**	Nb_2PC	Nb_6C_5	C
**$Nb_5C_4S_2$**	Nb_3S_4	Nb_6C_5	C
**$Nb_5C_4Cl_2$**	Nb_3Cl_8	Nb_6C_5	C
**$Nb_5C_4As_2$**	Nb_6C_5	Nb_2AsC	C
**$Nb_5C_4Se_2$**	Nb_3Se_4	Nb_6C_5	C
**$Nb_5C_4Br_2$**	Nb_3Br_8	Nb_6C_5	C
**$Nb_5C_4Sb_2$**	Nb_6C_5	$NbSb_2$	C
**$Nb_5C_4Te_2$**	$NbTe_2$	Nb_6C_5	C
**$Nb_5C_4I_2$**	Nb_3I_8	Nb_6C_5	C
**Nb_2N**	Nb_2N		
**Nb_2NO_2**	NbO_2	NbN	
**Nb_2NF_2**	Nb_2F_5	NbN	Nb_2N
**Nb_2NP_2**	Nb_5N_6	NbP	P_3N_5

**Nb_2NS_2**	$Nb_{25}S_{48}$	Nb_3S_5	Nb_5N_6
**Nb_2NCl_2**	NbN	Nb_3Cl_7	Nb_2N
**Nb_2NAs_2**	$NbAs_2$	NbN	
**Nb_2NSe_2**	$NbSe_2$	NbN	
**Nb_2NBr_2**	Nb_2N	Nb_3Br_8	NbN
**Nb_2NSb_2**	$NbSb_2$	NbN	
**Nb_2NTe_2**	$NbTe_2$	NbN	
**Nb_2NI_2**	Nb_3I_8	Nb_2N	NbN
**Nb_3N_2**	Nb_2N	NbN	
**$Nb_3N_2O_2$**	NbO_2	NbN	
**$Nb_3N_2F_2$**	Nb_2F_5	NbN	Nb_2N
**$Nb_3N_2P_2$**	Nb_5N_6	NbP	P_3N_5
**$Nb_3N_2S_2$**	Nb_3S_5	Nb_5N_6	NbN
**$Nb_3N_2Cl_2$**	NbN	Nb_3Cl_7	Nb_2N
**$Nb_3N_2As_2$**	$NbAs_2$	NbN	
**$Nb_3N_2Se_2$**	$NbSe_2$	NbN	
**$Nb_3N_2Br_2$**	Nb_2N	Nb_3Br_8	NbN
**$Nb_3N_2Sb_2$**	$NbSb_2$	NbN	
**$Nb_3N_2Te_2$**	$NbTe_2$	NbN	
**$Nb_3N_2I_2$**	Nb_3I_8	Nb_2N	NbN
**Nb_4N_3**	Nb_2N	NbN	
**$Nb_4N_3O_2$**	NbO_2	NbN	
**$Nb_4N_3F_2$**	Nb_2F_5	NbN	Nb_2N
**$Nb_4N_3P_2$**	Nb_5N_6	NbP	P_3N_5
**$Nb_4N_3S_2$**	Nb_3S_5	Nb_5N_6	NbN
**$Nb_4N_3Cl_2$**	NbN	Nb_3Cl_7	Nb_2N
**$Nb_4N_3As_2$**	$NbAs_2$	NbN	
**$Nb_4N_3Se_2$**	$NbSe_2$	NbN	
**$Nb_4N_3Br_2$**	Nb_2N	Nb_3Br_8	NbN
**$Nb_4N_3Sb_2$**	$NbSb_2$	NbN	
**$Nb_4N_3Te_2$**	$NbTe_2$	NbN	
**$Nb_4N_3I_2$**	Nb_3I_8	Nb_2N	NbN
**Nb_5N_4**	Nb_2N	NbN	
**$Nb_5N_4O_2$**	NbO_2	NbN	
**$Nb_5N_4F_2$**	Nb_2F_5	NbN	Nb_2N
**$Nb_5N_4P_2$**	Nb_5N_6	NbP	P_3N_5
**$Nb_5N_4S_2$**	Nb_3S_5	Nb_5N_6	NbN
**$Nb_5N_4Cl_2$**	NbN	Nb_3Cl_7	Nb_2N
**$Nb_5N_4As_2$**	$NbAs_2$	NbN	
**$Nb_5N_4Se_2$**	$NbSe_2$	NbN	
**$Nb_5N_4Br_2$**	Nb_2N	Nb_3Br_8	NbN

**$Nb_5N_4Sb_2$**	$NbSb_2$	NbN	
**$Nb_5N_4Te_2$**	$NbTe_2$	NbN	
**$Nb_5N_4I_2$**	Nb_3I_8	Nb_2N	NbN
**Mo_2C**	Mo_2C		
**Mo_2CO_2**	Mo_2C	C	MoO_2
**Mo_2CF_2**	Mo_2C	C	MoF_3
**Mo_2CP_2**	MoP	C	
**Mo_2CS_2**	Mo_2C	C	MoS_2
**Mo_2CCl_2**	Mo_2C	C	$MoCl_2$
**Mo_2CAs_2**	Mo_5As_4	Mo_2As_3	C
**Mo_2CSe_2**	Mo_2C	$MoSe_2$	C
**Mo_2CBr_2**	Mo_2C	C	$MoBr_2$
**Mo_2CSb_2**	Mo_2C	Sb_7Mo_3	C
**Mo_2CTe_2**	Mo_2C	Te_2Mo	C
**Mo_2CI_2**	Mo_2C	C	MoI_2
**Mo_3C_2**	Mo_2C	C	
**$Mo_3C_2O_2$**	Mo_2C	C	MoO_2
**$Mo_3C_2F_2$**	Mo_2C	C	MoF_3
**$Mo_3C_2P_2$**	Mo_2C	MoP	C
**$Mo_3C_2S_2$**	Mo_2C	C	MoS_2
**$Mo_3C_2Cl_2$**	Mo_2C	C	$MoCl_2$
**$Mo_3C_2As_2$**	Mo_5As_4	Mo_2C	C
**$Mo_3C_2Se_2$**	Mo_2C	$MoSe_2$	C
**$Mo_3C_2Br_2$**	Mo_2C	C	$MoBr_2$
**$Mo_3C_2Sb_2$**	Mo_2C	Sb_7Mo_3	C
**$Mo_3C_2Te_2$**	Mo_2C	Te_2Mo	C
**$Mo_3C_2I_2$**	Mo_2C	C	MoI_2
**Mo_4C_3**	Mo_2C	C	
**$Mo_4C_3O_2$**	Mo_2C	C	MoO_2
**$Mo_4C_3F_2$**	Mo_2C	C	MoF_3
**$Mo_4C_3P_2$**	Mo_2C	MoP	C
**$Mo_4C_3S_2$**	Mo_2C	C	MoS_2
**$Mo_4C_3Cl_2$**	Mo_2C	C	$MoCl_2$
**$Mo_4C_3As_2$**	Mo_5As_4	Mo_2C	C
**$Mo_4C_3Se_2$**	Mo_2C	$MoSe_2$	C
**$Mo_4C_3Br_2$**	Mo_2C	C	$MoBr_2$
**$Mo_4C_3Sb_2$**	Mo_2C	Sb_7Mo_3	C
**$Mo_4C_3Te_2$**	Mo_2C	Te_2Mo	C
**$Mo_4C_3I_2$**	Mo_2C	C	MoI_2
**Mo_5C_4**	Mo_2C	C	
**$Mo_5C_4O_2$**	Mo_2C	C	MoO_2

**$Mo_5C_4F_2$**	Mo_2C	C	MoF_3
**$Mo_5C_4P_2$**	Mo_2C	MoP	C
**$Mo_5C_4S_2$**	Mo_2C	C	MoS_2
**$Mo_5C_4Cl_2$**	Mo_2C	C	$MoCl_2$
**$Mo_5C_4As_2$**	Mo_5As_4	Mo_2C	C
**$Mo_5C_4Se_2$**	Mo_2C	$MoSe_2$	C
**$Mo_5C_4Br_2$**	Mo_2C	C	$MoBr_2$
**$Mo_5C_4Sb_2$**	Mo_2C	Sb_7Mo_3	C
**$Mo_5C_4Te_2$**	Mo_2C	Te_2Mo	C
**$Mo_5C_4I_2$**	Mo_2C	C	MoI_2
**Mo_2N**	MoN	Mo	
**Mo_2NO_2**	MoN	MoO_2	
**Mo_2NF_2**	MoN	Mo	MoF_3
**Mo_2NP_2**	MoP	P_3N_5	MoN
**Mo_2NS_2**	MoS_2	MoN	
**Mo_2NCl_2**	MoN	$MoCl_2$	
**Mo_2NAs_2**	$MoAs_2$	MoN	
**Mo_2NSe_2**	$MoSe_2$	MoN	
**Mo_2NBr_2**	$MoBr_2$	MoN	
**Mo_2NSb_2**	MoN	Sb_7Mo_3	Mo
**Mo_2NTe_2**	MoN	Te_2Mo	
**Mo_2NI_2**	MoI_2	MoN	
**Mo_3N_2**	MoN	Mo	
**$Mo_3N_2O_2$**	MoN	MoO_2	
**$Mo_3N_2F_2$**	MoN	Mo	MoF_3
**$Mo_3N_2P_2$**	MoP	P_3N_5	MoN
**$Mo_3N_2S_2$**	MoS_2	MoN	
**$Mo_3N_2Cl_2$**	MoN	$MoCl_2$	
**$Mo_3N_2As_2$**	$MoAs_2$	MoN	
**$Mo_3N_2Se_2$**	$MoSe_2$	MoN	
**$Mo_3N_2Br_2$**	$MoBr_2$	MoN	
**$Mo_3N_2Sb_2$**	MoN	Sb_7Mo_3	Mo
**$Mo_3N_2Te_2$**	MoN	Te_2Mo	
**$Mo_3N_2I_2$**	MoI_2	MoN	
**Mo_4N_3**	MoN	Mo	
**$Mo_4N_3O_2$**	MoN	MoO_2	
**$Mo_4N_3F_2$**	MoN	Mo	MoF_3
**$Mo_4N_3P_2$**	MoP	P_3N_5	MoN
**$Mo_4N_3S_2$**	MoS_2	MoN	
**$Mo_4N_3Cl_2$**	MoN	$MoCl_2$	
**$Mo_4N_3As_2$**	$MoAs_2$	MoN	

**$Mo_4N_3Se_2$**	$MoSe_2$	MoN	
**$Mo_4N_3Br_2$**	$MoBr_2$	MoN	
**$Mo_4N_3Sb_2$**	MoN	Sb_7Mo_3	Mo
**$Mo_4N_3Te_2$**	MoN	Te_2Mo	
**$Mo_4N_3I_2$**	MoI_2	MoN	
**Mo_5N_4**	MoN	Mo	
**$Mo_5N_4O_2$**	MoN	MoO_2	
**$Mo_5N_4F_2$**	MoN	Mo	MoF_3
**$Mo_5N_4P_2$**	MoP	P_3N_5	MoN
**$Mo_5N_4S_2$**	MoS_2	MoN	
**$Mo_5N_4Cl_2$**	MoN	$MoCl_2$	
**$Mo_5N_4As_2$**	$MoAs_2$	MoN	
**$Mo_5N_4Se_2$**	$MoSe_2$	MoN	
**$Mo_5N_4Br_2$**	$MoBr_2$	MoN	
**$Mo_5N_4Sb_2$**	MoN	Sb_7Mo_3	Mo
**$Mo_5N_4Te_2$**	MoN	Te_2Mo	
**$Mo_5N_4I_2$**	MoI_2	MoN	
**Hf_2C**	HfC	Hf	
**Hf_2CO_2**	HfC	HfO_2	
**Hf_2CF_2**	Hf	HfC	HfF_4
**Hf_2CP_2**	HfP	C	
**Hf_2CS_2**	Hf_2CS	HfS_2	C
**Hf_2CCl_2**	Hf_6CCl_{14}	Hf	HfC
**Hf_2CAs_2**	$HfAs_2$	HfC	
**Hf_2CSe_2**	HfC	$HfSe_2$	
**Hf_2CBr_2**	$HfBr_4$	Hf	HfC
**Hf_2CSb_2**	$HfSb_2$	HfC	
**Hf_2CTe_2**	$HfTe_2$	HfC	
**Hf_2CI_2**	Hf	HfC	HfI_3
**Hf_3C_2**	HfC	Hf	
**$Hf_3C_2O_2$**	HfC	HfO_2	
**$Hf_3C_2F_2$**	Hf	HfC	HfF_4
**$Hf_3C_2P_2$**	HfP	C	HfC
**$Hf_3C_2S_2$**	Hf_2CS	HfS_2	C
**$Hf_3C_2Cl_2$**	Hf_6CCl_{14}	Hf	HfC
**$Hf_3C_2As_2$**	$HfAs_2$	HfC	
**$Hf_3C_2Se_2$**	HfC	$HfSe_2$	
**$Hf_3C_2Br_2$**	$HfBr_4$	Hf	HfC
**$Hf_3C_2Sb_2$**	$HfSb_2$	HfC	
**$Hf_3C_2Te_2$**	$HfTe_2$	HfC	
**$Hf_3C_2I_2$**	Hf	HfC	HfI_3

**Hf_4C_3**	HfC	Hf	
**$Hf_4C_3O_2$**	HfC	HfO_2	
**$Hf_4C_3F_2$**	Hf	HfC	HfF_4
**$Hf_4C_3P_2$**	HfP	C	HfC
**$Hf_4C_3S_2$**	Hf_2CS	C	
**$Hf_4C_3Cl_2$**	Hf_6CCl_{14}	Hf	HfC
**$Hf_4C_3As_2$**	$HfAs_2$	HfC	
**$Hf_4C_3Se_2$**	HfC	$HfSe_2$	
**$Hf_4C_3Br_2$**	$HfBr_4$	Hf	HfC
**$Hf_4C_3Sb_2$**	$HfSb_2$	HfC	
**$Hf_4C_3Te_2$**	$HfTe_2$	HfC	
**$Hf_4C_3I_2$**	Hf	HfC	HfI_3
**Hf_5C_4**	HfC	Hf	
**$Hf_5C_4O_2$**	HfC	HfO_2	
**$Hf_5C_4F_2$**	Hf	HfC	HfF_4
**$Hf_5C_4P_2$**	HfP	C	HfC
**$Hf_5C_4S_2$**	Hf_2CS	HfC	C
**$Hf_5C_4Cl_2$**	Hf_6CCl_{14}	Hf	HfC
**$Hf_5C_4As_2$**	$HfAs_2$	HfC	
**$Hf_5C_4Se_2$**	HfC	$HfSe_2$	
**$Hf_5C_4Br_2$**	$HfBr_4$	Hf	HfC
**$Hf_5C_4Sb_2$**	$HfSb_2$	HfC	
**$Hf_5C_4Te_2$**	$HfTe_2$	HfC	
**$Hf_5C_4I_2$**	Hf	HfC	HfI_3
**Hf_2N**	Hf_2N		
**Hf_2NO_2**	HfN	HfO_2	
**Hf_2NF_2**	Hf_3N_2	HfF_4	
**Hf_2NP_2**	HfP_2	Hf_3N_4	HfP
**Hf_2NS_2**	HfS	Hf_2SN_2	HfS_2
**Hf_2NCl_2**	Hf_3N_2	$HfCl_4$	
**Hf_2NAs_2**	$HfAs_2$	HfN	
**Hf_2NSe_2**	$HfSe_2$	HfN	
**Hf_2NBr_2**	Hf_3N_2	$HfBr_4$	
**Hf_2NSb_2**	$HfSb_2$	HfN	
**Hf_2NTe_2**	$HfTe_2$	HfN	
**Hf_2NI_2**	HfI_3	Hf_3N_2	HfN
**Hf_3N_2**	Hf_3N_2		
**$Hf_3N_2O_2$**	HfN	HfO_2	
**$Hf_3N_2F_2$**	Hf_3N_2	HfN	HfF_4
**$Hf_3N_2P_2$**	HfP_2	Hf_3N_4	HfP
**$Hf_3N_2S_2$**	HfS	Hf_2SN_2	

**$Hf_3N_2Cl_2$**	Hf_3N_2	HfN	$HfCl_4$
**$Hf_3N_2As_2$**	$HfAs_2$	HfN	
**$Hf_3N_2Se_2$**	$HfSe_2$	HfN	
**$Hf_3N_2Br_2$**	Hf_3N_2	$HfBr_4$	HfN
**$Hf_3N_2Sb_2$**	$HfSb_2$	HfN	
**$Hf_3N_2Te_2$**	$HfTe_2$	HfN	
**$Hf_3N_2I_2$**	HfI_3	Hf_3N_2	HfN
**Hf_4N_3**	Hf_3N_2	HfN	
**$Hf_4N_3O_2$**	HfN	HfO_2	
**$Hf_4N_3F_2$**	Hf_3N_2	HfN	HfF_4
**$Hf_4N_3P_2$**	HfP_2	Hf_3N_4	HfP
**$Hf_4N_3S_2$**	HfS	Hf_2SN_2	HfN
**$Hf_4N_3Cl_2$**	Hf_3N_2	HfN	$HfCl_4$
**$Hf_4N_3As_2$**	$HfAs_2$	HfN	
**$Hf_4N_3Se_2$**	$HfSe_2$	HfN	
**$Hf_4N_3Br_2$**	Hf_3N_2	$HfBr_4$	HfN
**$Hf_4N_3Sb_2$**	$HfSb_2$	HfN	
**$Hf_4N_3Te_2$**	$HfTe_2$	HfN	
**$Hf_4N_3I_2$**	HfI_3	Hf_3N_2	HfN
**Hf_5N_4**	Hf_3N_2	HfN	
**$Hf_5N_4O_2$**	HfN	HfO_2	
**$Hf_5N_4F_2$**	Hf_3N_2	HfN	HfF_4
**$Hf_5N_4P_2$**	Hf_3N_4	HfP	
**$Hf_5N_4S_2$**	HfS	Hf_2SN_2	HfN
**$Hf_5N_4Cl_2$**	Hf_3N_2	HfN	$HfCl_4$
**$Hf_5N_4As_2$**	$HfAs_2$	HfN	
**$Hf_5N_4Se_2$**	$HfSe_2$	HfN	
**$Hf_5N_4Br_2$**	Hf_3N_2	$HfBr_4$	HfN
**$Hf_5N_4Sb_2$**	$HfSb_2$	HfN	
**$Hf_5N_4Te_2$**	$HfTe_2$	HfN	
**$Hf_5N_4I_2$**	HfI_3	Hf_3N_2	HfN
**Ta_2C**	Ta_2C		
**Ta_2CO_2**	TaC	Ta_2C	Ta_2O_5
**Ta_2CF_2**	TaC	TaF_5	Ta_2C
**Ta_2CP_2**	TaP	C	
**Ta_2CS_2**	Ta_2CS_2		
**Ta_2CCl_2**	Ta_2Cl_5	TaC	Ta_2C
**Ta_2CAs_2**	$TaAs_2$	TaC	
**Ta_2CSe_2**	TaC	$TaSe_2$	
**Ta_2CBr_2**	Ta_2Br_5	TaC	Ta_2C
**Ta_2CSb_2**	$TaSb_2$	TaC	

**Ta_2CTe_2**	$TaTe_2$	TaC	
**Ta_2CI_2**	TaC	TaI_5	Ta_2C
**Ta_3C_2**	TaC	Ta_2C	
**$Ta_3C_2O_2$**	TaC	Ta_2C	Ta_2O_5
**$Ta_3C_2F_2$**	TaC	TaF_5	Ta_2C
**$Ta_3C_2P_2$**	TaP	C	TaC
**$Ta_3C_2S_2$**	Ta_2CS_2	TaC	
**$Ta_3C_2Cl_2$**	Ta_2Cl_5	TaC	Ta_2C
**$Ta_3C_2As_2$**	$TaAs_2$	TaC	
**$Ta_3C_2Se_2$**	TaC	$TaSe_2$	
**$Ta_3C_2Br_2$**	Ta_2Br_5	TaC	Ta_2C
**$Ta_3C_2Sb_2$**	$TaSb_2$	TaC	
**$Ta_3C_2Te_2$**	$TaTe_2$	TaC	
**$Ta_3C_2I_2$**	TaC	TaI_5	Ta_2C
**Ta_4C_3**	TaC	Ta_2C	
**$Ta_4C_3O_2$**	TaC	Ta_2C	Ta_2O_5
**$Ta_4C_3F_2$**	TaC	TaF_5	Ta_2C
**$Ta_4C_3P_2$**	TaP	C	TaC
**$Ta_4C_3S_2$**	Ta_2CS_2	TaC	
**$Ta_4C_3Cl_2$**	Ta_2Cl_5	TaC	Ta_2C
**$Ta_4C_3As_2$**	$TaAs_2$	TaC	
**$Ta_4C_3Se_2$**	TaC	$TaSe_2$	
**$Ta_4C_3Br_2$**	Ta_2Br_5	TaC	Ta_2C
**$Ta_4C_3Sb_2$**	$TaSb_2$	TaC	
**$Ta_4C_3Te_2$**	$TaTe_2$	TaC	
**$Ta_4C_3I_2$**	TaC	TaI_5	Ta_2C
**Ta_5C_4**	TaC	Ta_2C	
**$Ta_5C_4O_2$**	TaC	Ta_2C	Ta_2O_5
**$Ta_5C_4F_2$**	TaC	TaF_5	Ta_2C
**$Ta_5C_4P_2$**	TaP	C	TaC
**$Ta_5C_4S_2$**	Ta_2CS_2	TaC	
**$Ta_5C_4Cl_2$**	Ta_2Cl_5	TaC	Ta_2C
**$Ta_5C_4As_2$**	$TaAs_2$	TaC	
**$Ta_5C_4Se_2$**	TaC	$TaSe_2$	
**$Ta_5C_4Br_2$**	Ta_2Br_5	TaC	Ta_2C
**$Ta_5C_4Sb_2$**	$TaSb_2$	TaC	
**$Ta_5C_4Te_2$**	$TaTe_2$	TaC	
**$Ta_5C_4I_2$**	TaC	TaI_5	Ta_2C
**Ta_2N**	Ta_2N		
**Ta_2NO_2**	Ta_2N	TaN	Ta_2O_5
**Ta_2NF_2**	Ta_2N	TaF_5	TaN

**Ta_2NP_2**	Ta_3N_5	TaP	P
**Ta_2NS_2**	Ta_5N_6	$Ta_{27}S_{50}$	TaN
**Ta_2NCl_2**	Ta_2Cl_5	Ta_2N	TaN
**Ta_2NAs_2**	$TaAs_2$	TaN	
**Ta_2NSe_2**	$TaSe_2$	TaN	
**Ta_2NBr_2**	Ta_2Br_5	Ta_2N	TaN
**Ta_2NSb_2**	$TaSb_2$	TaN	
**Ta_2NTe_2**	$TaTe_2$	TaN	
**Ta_2NI_2**	Ta_3I_7	Ta_2N	TaN
**Ta_3N_2**	Ta_2N	TaN	
**$Ta_3N_2O_2$**	Ta_2N	TaN	Ta_2O_5
**$Ta_3N_2F_2$**	Ta_2N	TaF_5	TaN
**$Ta_3N_2P_2$**	Ta_3N_5	TaP	P
**$Ta_3N_2S_2$**	Ta_5N_6	$Ta_{27}S_{50}$	TaN
**$Ta_3N_2Cl_2$**	Ta_2Cl_5	Ta_2N	TaN
**$Ta_3N_2As_2$**	$TaAs_2$	TaN	
**$Ta_3N_2Se_2$**	$TaSe_2$	TaN	
**$Ta_3N_2Br_2$**	Ta_2Br_5	Ta_2N	TaN
**$Ta_3N_2Sb_2$**	$TaSb_2$	TaN	
**$Ta_3N_2Te_2$**	$TaTe_2$	TaN	
**$Ta_3N_2I_2$**	Ta_3I_7	Ta_2N	TaN
**Ta_4N_3**	Ta_2N	TaN	
**$Ta_4N_3O_2$**	Ta_2N	TaN	Ta_2O_5
**$Ta_4N_3F_2$**	Ta_2N	TaF_5	TaN
**$Ta_4N_3P_2$**	Ta_5N_6	Ta_3N_5	TaP
**$Ta_4N_3S_2$**	Ta_5N_6	$Ta_{27}S_{50}$	TaN
**$Ta_4N_3Cl_2$**	Ta_2Cl_5	Ta_2N	TaN
**$Ta_4N_3As_2$**	$TaAs_2$	TaN	
**$Ta_4N_3Se_2$**	$TaSe_2$	TaN	
**$Ta_4N_3Br_2$**	Ta_2Br_5	Ta_2N	TaN
**$Ta_4N_3Sb_2$**	$TaSb_2$	TaN	
**$Ta_4N_3Te_2$**	$TaTe_2$	TaN	
**$Ta_4N_3I_2$**	Ta_3I_7	Ta_2N	TaN
**Ta_5N_4**	Ta_2N	TaN	
**$Ta_5N_4O_2$**	Ta_2N	TaN	Ta_2O_5
**$Ta_5N_4F_2$**	Ta_2N	TaF_5	TaN
**$Ta_5N_4P_2$**	Ta_5N_6	Ta_3N_5	TaP
**$Ta_5N_4S_2$**	Ta_5N_6	$Ta_{27}S_{50}$	TaN
**$Ta_5N_4Cl_2$**	Ta_2Cl_5	Ta_2N	TaN
**$Ta_5N_4As_2$**	$TaAs_2$	TaN	
**$Ta_5N_4Se_2$**	$TaSe_2$	TaN	

**$Ta_5N_4Br_2$**	Ta_2Br_5	Ta_2N	TaN
**$Ta_5N_4Sb_2$**	$TaSb_2$	TaN	
**$Ta_5N_4Te_2$**	$TaTe_2$	TaN	
**$Ta_5N_4I_2$**	Ta_3I_7	Ta_2N	TaN
**W_2C**	WC	W	
**W_2CO_2**	WC	WO_2	
**W_2CF_2**	WC	W	WF_4
**W_2CP_2**	PW	C	
**W_2CS_2**	WC	WS_2	
**W_2CCl_2**	W_6CCl_{18}	WC	W
**W_2CAs_2**	As_3W_2	WC	As
**W_2CSe_2**	WC	WSe_2	
**W_2CBr_2**	WC	WBr_2	
**W_2CSb_2**	WC	Sb	W
**W_2CTe_2**	WC	Te_2W	
**W_2CI_2**	WC	WI_2	
**W_3C_2**	WC	W	
**$W_3C_2O_2$**	WC	WO_2	
**$W_3C_2F_2$**	WC	W	WF_4
**$W_3C_2P_2$**	WC	PW	C
**$W_3C_2S_2$**	WC	WS_2	
**$W_3C_2Cl_2$**	W_6CCl_{18}	WC	W
**$W_3C_2As_2$**	As_3W_2	WC	As
**$W_3C_2Se_2$**	WC	WSe_2	
**$W_3C_2Br_2$**	WC	WBr_2	
**$W_3C_2Sb_2$**	WC	Sb	W
**$W_3C_2Te_2$**	WC	Te_2W	
**$W_3C_2I_2$**	WC	WI_2	
**W_4C_3**	WC	W	
**$W_4C_3O_2$**	WC	WO_2	
**$W_4C_3F_2$**	WC	W	WF_4
**$W_4C_3P_2$**	WC	PW	C
**$W_4C_3S_2$**	WC	WS_2	
**$W_4C_3Cl_2$**	W_6CCl_{18}	WC	W
**$W_4C_3As_2$**	As_3W_2	WC	As
**$W_4C_3Se_2$**	WC	WSe_2	
**$W_4C_3Br_2$**	WC	WBr_2	
**$W_4C_3Sb_2$**	WC	Sb	W
**$W_4C_3Te_2$**	WC	Te_2W	
**$W_4C_3I_2$**	WC	WI_2	
**W_5C_4**	WC	W	

**$W_5C_4O_2$**	WC	WO_2	
**$W_5C_4F_2$**	WC	W	WF_4
**$W_5C_4P_2$**	WC	PW	C
**$W_5C_4S_2$**	WC	WS_2	
**$W_5C_4Cl_2$**	W_6CCl_{18}	WC	W
**$W_5C_4As_2$**	As_3W_2	WC	As
**$W_5C_4Se_2$**	WC	WSe_2	
**$W_5C_4Br_2$**	WC	WBr_2	
**$W_5C_4Sb_2$**	WC	Sb	W
**$W_5C_4Te_2$**	WC	Te_2W	
**$W_5C_4I_2$**	WC	WI_2	
**W_2N**	W_2N_3	W	
**W_2NO_2**	W_2N_3	W	WO_2
**W_2NF_2**	W_2N_3	W	WF_4
**W_2NP_2**	PW	P_2W	W_2N_3
**W_2NS_2**	W_2N_3	WS_2	W
**W_2NCl_2**	W_2N_3	W	WCl_2
**W_2NAs_2**	As_3W_2	W_2N_3	
**W_2NSe_2**	W_2N_3	WSe_2	W
**W_2NBr_2**	WBr_2	W_2N_3	W
**W_2NSb_2**	W_2N_3	Sb	W
**W_2NTe_2**	W_2N_3	Te_2W	W
**W_2NI_2**	WI_2	W_2N_3	W
**W_3N_2**	W_2N_3	W	
**$W_3N_2O_2$**	W_2N_3	W	WO_2
**$W_3N_2F_2$**	W_2N_3	W	WF_4
**$W_3N_2P_2$**	PW	P_2W	W_2N_3
**$W_3N_2S_2$**	W_2N_3	WS_2	W
**$W_3N_2Cl_2$**	W_2N_3	W	WCl_2
**$W_3N_2As_2$**	As_3W_2	W_2N_3	W
**$W_3N_2Se_2$**	W_2N_3	WSe_2	W
**$W_3N_2Br_2$**	WBr_2	W_2N_3	W
**$W_3N_2Sb_2$**	W_2N_3	Sb	W
**$W_3N_2Te_2$**	W_2N_3	Te_2W	W
**$W_3N_2I_2$**	WI_2	W_2N_3	W
**W_4N_3**	W_2N_3	W	
**$W_4N_3O_2$**	W_2N_3	W	WO_2
**$W_4N_3F_2$**	W_2N_3	W	WF_4
**$W_4N_3P_2$**	PW	W_2N_3	
**$W_4N_3S_2$**	W_2N_3	WS_2	W
**$W_4N_3Cl_2$**	W_2N_3	W	WCl_2

**$W_4N_3As_2$**	As_3W_2	W_2N_3	W
**$W_4N_3Se_2$**	W_2N_3	WSe_2	W
**$W_4N_3Br_2$**	WBr_2	W_2N_3	W
**$W_4N_3Sb_2$**	W_2N_3	Sb	W
**$W_4N_3Te_2$**	W_2N_3	Te_2W	W
**$W_4N_3I_2$**	WI_2	W_2N_3	W
**W_5N_4**	W_2N_3	W	
**$W_5N_4O_2$**	W_2N_3	W	WO_2
**$W_5N_4F_2$**	W_2N_3	W	WF_4
**$W_5N_4P_2$**	PW	W_2N_3	W
**$W_5N_4S_2$**	W_2N_3	WS_2	W
**$W_5N_4Cl_2$**	W_2N_3	W	WCl_2
**$W_5N_4As_2$**	As_3W_2	W_2N_3	W
**$W_5N_4Se_2$**	W_2N_3	WSe_2	W
**$W_5N_4Br_2$**	WBr_2	W_2N_3	W
**$W_5N_4Sb_2$**	W_2N_3	Sb	W
**$W_5N_4Te_2$**	W_2N_3	Te_2W	W
**$W_5N_4I_2$**	WI_2	W_2N_3	W
**Re_2C**	Re_2C		
**Re_2CO_2**	ReO_3	Re_2C	C
**Re_2CF_2**	Re_2C	C	ReF_6
**Re_2CP_2**	Re_2P	Re_3P_4	C
**Re_2CS_2**	Re_2C	C	ReS_2
**Re_2CCl_2**	Re_2C	C	$ReCl_3$
**Re_2CAs_2**	Re_3As_7	C	Re_2C
**Re_2CSe_2**	Re_2C	C	$ReSe_2$
**Re_2CBr_2**	Re_2C	C	Re_3Br_7
**Re_2CSb_2**	Sb	Re_2C	
**Re_2CTe_2**	Re_2Te_5	C	Re_2C
**Re_2CI_2**	Re_2C	C	ReI_3
**Re_3C_2**	Re_2C	C	
**$Re_3C_2O_2$**	ReO_3	Re_2C	C
**$Re_3C_2F_2$**	Re_2C	C	ReF_6
**$Re_3C_2P_2$**	Re_2P	Re_3P_4	C
**$Re_3C_2S_2$**	Re_2C	C	ReS_2
**$Re_3C_2Cl_2$**	Re_2C	C	$ReCl_3$
**$Re_3C_2As_2$**	Re_3As_7	C	Re_2C
**$Re_3C_2Se_2$**	Re_2C	C	$ReSe_2$
**$Re_3C_2Br_2$**	Re_2C	C	Re_3Br_7
**$Re_3C_2Sb_2$**	C	Sb	Re_2C
**$Re_3C_2Te_2$**	Re_2Te_5	C	Re_2C

**$Re_3C_2I_2$**	Re_2C	C	ReI_3
**Re_4C_3**	Re_2C	C	
**$Re_4C_3O_2$**	ReO_3	Re_2C	C
**$Re_4C_3F_2$**	Re_2C	C	ReF_6
**$Re_4C_3P_2$**	Re_2P	C	
**$Re_4C_3S_2$**	Re_2C	C	ReS_2
**$Re_4C_3Cl_2$**	Re_2C	C	$ReCl_3$
**$Re_4C_3As_2$**	Re_3As_7	C	Re_2C
**$Re_4C_3Se_2$**	Re_2C	C	$ReSe_2$
**$Re_4C_3Br_2$**	Re_2C	C	Re_3Br_7
**$Re_4C_3Sb_2$**	C	Sb	Re_2C
**$Re_4C_3Te_2$**	Re_2Te_5	C	Re_2C
**$Re_4C_3I_2$**	Re_2C	C	ReI_3
**Re_5C_4**	Re_2C	C	
**$Re_5C_4O_2$**	ReO_3	Re_2C	C
**$Re_5C_4F_2$**	Re_2C	C	ReF_6
**$Re_5C_4P_2$**	Re_2P	C	Re_2C
**$Re_5C_4S_2$**	Re_2C	C	ReS_2
**$Re_5C_4Cl_2$**	Re_2C	C	$ReCl_3$
**$Re_5C_4As_2$**	Re_3As_7	C	Re_2C
**$Re_5C_4Se_2$**	Re_2C	C	$ReSe_2$
**$Re_5C_4Br_2$**	Re_2C	C	Re_3Br_7
**$Re_5C_4Sb_2$**	C	Sb	Re_2C
**$Re_5C_4Te_2$**	Re_2Te_5	C	Re_2C
**$Re_5C_4I_2$**	Re_2C	C	ReI_3
**Re_2N**	Re_3N	ReN	
**Re_2NO_2**	Re_3N	ReO_3	ReN
**Re_2NF_2**	Re_3N	ReN	ReF_6
**Re_2NP_2**	Re_2P	Re_3P_4	P_3N_5
**Re_2NS_2**	ReS_2	ReN	
**Re_2NCl_2**	Re_3N	ReN	$ReCl_3$
**Re_2NAs_2**	Re_3N	Re_3As_7	ReN
**Re_2NSe_2**	$ReSe_2$	ReN	
**Re_2NBr_2**	Re_3N	Re_3Br_7	ReN
**Re_2NSb_2**	Re_3N	Sb	ReN
**Re_2NTe_2**	Re_3N	Re_2Te_5	ReN
**Re_2NI_2**	Re_3N	IN_4	ReI_3
**Re_3N_2**	Re_3N	ReN	
**$Re_3N_2O_2$**	Re_3N	ReO_3	ReN
**$Re_3N_2F_2$**	Re_3N	ReN	ReF_6
**$Re_3N_2P_2$**	Re_2P	Re_3N	P_3N_5

**$Re_3N_2S_2$**	ReS_2	ReN	
**$Re_3N_2Cl_2$**	Re_3N	ReN	$ReCl_3$
**$Re_3N_2As_2$**	Re_3N	Re_3As_7	ReN
**$Re_3N_2Se_2$**	$ReSe_2$	ReN	
**$Re_3N_2Br_2$**	Re_3N	Re_3Br_7	ReN
**$Re_3N_2Sb_2$**	Re_3N	Sb	ReN
**$Re_3N_2Te_2$**	Re_3N	Re_2Te_5	ReN
**$Re_3N_2I_2$**	Re_3N	IN_4	ReI_3
**Re_4N_3**	Re_3N	ReN	
**$Re_4N_3O_2$**	Re_3N	ReO_3	ReN
**$Re_4N_3F_2$**	Re_3N	ReN	ReF_6
**$Re_4N_3P_2$**	Re_2P	Re_3N	P_3N_5
**$Re_4N_3S_2$**	ReS_2	ReN	
**$Re_4N_3Cl_2$**	Re_3N	ReN	$ReCl_3$
**$Re_4N_3As_2$**	Re_3N	Re_3As_7	ReN
**$Re_4N_3Se_2$**	$ReSe_2$	ReN	
**$Re_4N_3Br_2$**	Re_3N	Re_3Br_7	ReN
**$Re_4N_3Sb_2$**	Re_3N	Sb	ReN
**$Re_4N_3Te_2$**	Re_3N	Re_2Te_5	ReN
**$Re_4N_3I_2$**	Re_3N	IN_4	ReI_3
**Re_5N_4**	Re_3N	ReN	
**$Re_5N_4O_2$**	Re_3N	ReO_3	ReN
**$Re_5N_4F_2$**	Re_3N	ReN	ReF_6
**$Re_5N_4P_2$**	Re_2P	Re_3N	P_3N_5
**$Re_5N_4S_2$**	ReS_2	ReN	
**$Re_5N_4Cl_2$**	Re_3N	ReN	$ReCl_3$
**$Re_5N_4As_2$**	Re_3N	Re_3As_7	ReN
**$Re_5N_4Se_2$**	$ReSe_2$	ReN	
**$Re_5N_4Br_2$**	Re_3N	Re_3Br_7	ReN
**$Re_5N_4Sb_2$**	Re_3N	Sb	ReN
**$Re_5N_4Te_2$**	Re_3N	Re_2Te_5	ReN
**$Re_5N_4I_2$**	Re_3N	IN_4	ReI_3
**La_2C**	La_2C_3	La	
**La_2CO_2**	La_2O_3	La_2C_3	
**La_2CF_2**	La_2C_3	LaF_3	La
**La_2CP_2**	LaP	C	
**La_2CS_2**	C	LaS	
**La_2CCl_2**	$La_4C_2Cl_5$	$La_{14}C_{14}Cl_9$	La
**La_2CAs_2**	LaAs	C	
**La_2CSe_2**	C	LaSe	
**La_2CBr_2**	$La_4C_2Br_5$	$La_3C_2Br_3$	La

**La_2CSb_2**	$LaSb$	C	
**La_2CTe_2**	$LaTe$	C	
**La_2CI_2**	$La_4C_2I_5$	$La_3C_2I_3$	La
**La_3C_2**	La_2C_3	La	
**$La_3C_2O_2$**	La_2O_3	La_2C_3	La
**$La_3C_2F_2$**	La_2C_3	LaF_3	La
**$La_3C_2P_2$**	LaP	LaC_2	
**$La_3C_2S_2$**	LaC_2	LaS	
**$La_3C_2Cl_2$**	$La_4C_2Cl_5$	$La_{14}C_{14}Cl_9$	La
**$La_3C_2As_2$**	$LaAs$	LaC_2	
**$La_3C_2Se_2$**	LaC_2	$LaSe$	
**$La_3C_2Br_2$**	La_2C_3	$La_3C_2Br_3$	La
**$La_3C_2Sb_2$**	$LaSb$	LaC_2	
**$La_3C_2Te_2$**	$LaTe$	LaC_2	
**$La_3C_2I_2$**	$La_3C_2I_3$	La_2C_3	La
**La_4C_3**	La_2C_3	La	
**$La_4C_3O_2$**	La_2O_3	La_2C_3	La
**$La_4C_3F_2$**	La_2C_3	LaF_3	La
**$La_4C_3P_2$**	La_2C_3	LaP	
**$La_4C_3S_2$**	La_2C_3	LaS	
**$La_4C_3Cl_2$**	$La_4C_2Cl_5$	$La_{14}C_{14}Cl_9$	La
**$La_4C_3As_2$**	La_2C_3	$LaAs$	
**$La_4C_3Se_2$**	La_2C_3	$LaSe$	
**$La_4C_3Br_2$**	La_2C_3	$La_3C_2Br_3$	La
**$La_4C_3Sb_2$**	La_4Sb_3	$LaSb$	LaC_2
**$La_4C_3Te_2$**	La_2C_3	$LaTe$	
**$La_4C_3I_2$**	$La_3C_2I_3$	La_2C_3	La
**La_5C_4**	La_2C_3	La	
**$La_5C_4O_2$**	La_2O_3	La_2C_3	La
**$La_5C_4F_2$**	La_2C_3	LaF_3	La
**$La_5C_4P_2$**	La_2C_3	La_2PC	LaP
**$La_5C_4S_2$**	La_2C_3	La	LaS
**$La_5C_4Cl_2$**	La_2C_2Cl	La	
**$La_5C_4As_2$**	La_2C_3	$LaAs$	La
**$La_5C_4Se_2$**	La_2C_3	La	$LaSe$
**$La_5C_4Br_2$**	La_2C_3	$La_3C_2Br_3$	La
**$La_5C_4Sb_2$**	La_4Sb_3	La_2C_3	LaC_2
**$La_5C_4Te_2$**	La_2C_3	$LaTe$	La
**$La_5C_4I_2$**	$La_3C_2I_3$	La_2C_3	La
**La_2N**	LaN	La	
**La_2NO_2**	LaN	La_2O_3	N_2

**La_2NF_2**	LaN	LaF_3	La
**La_2NP_2**	LaP	P_3N_5	LaN
**La_2NS_2**	La_3S_3N	N_2	
**La_2NCl_2**	La_2NCl_3	LaN	La
**La_2NAs_2**	LaAs	N_2	
**La_2NSe_2**	La_5Se_6N	$La_4Se_3N_2$	N_2
**La_2NBr_2**	La_3Br_6N	La	LaN
**La_2NSb_2**	$LaSb_2$	LaN	
**La_2NTe_2**	$La_4Te_3N_2$	$LaTe_2$	N_2
**La_2NI_2**	LaI_2	LaN	
**La_3N_2**	LaN	La	
**$La_3N_2O_2$**	LaN	La_2O_3	N_2
**$La_3N_2F_2$**	LaN	LaF_3	La
**$La_3N_2P_2$**	LaP	P_3N_5	LaN
**$La_3N_2S_2$**	$La_4S_3N_2$	N_2	LaN
**$La_3N_2Cl_2$**	La_2NCl_3	LaN	La
**$La_3N_2As_2$**	LaAs	N_2	LaN
**$La_3N_2Se_2$**	$La_4Se_3N_2$	N_2	LaN
**$La_3N_2Br_2$**	La_3Br_6N	La	LaN
**$La_3N_2Sb_2$**	$LaSb_2$	LaN	
**$La_3N_2Te_2$**	$La_4Te_3N_2$	N_2	LaN
**$La_3N_2I_2$**	LaI_2	LaN	
**La_4N_3**	LaN	La	
**$La_4N_3O_2$**	LaN	La_2O_3	N_2
**$La_4N_3F_2$**	LaN	LaF_3	La
**$La_4N_3P_2$**	LaP	P_3N_5	LaN
**$La_4N_3S_2$**	$La_4S_3N_2$	N_2	LaN
**$La_4N_3Cl_2$**	La_2NCl_3	LaN	La
**$La_4N_3As_2$**	LaAs	N_2	LaN
**$La_4N_3Se_2$**	$La_4Se_3N_2$	N_2	LaN
**$La_4N_3Br_2$**	La_3Br_6N	La	LaN
**$La_4N_3Sb_2$**	$LaSb_2$	LaN	
**$La_4N_3Te_2$**	$La_4Te_3N_2$	N_2	LaN
**$La_4N_3I_2$**	LaI_2	LaN	
**La_5N_4**	LaN	La	
**$La_5N_4O_2$**	LaN	La_2O_3	N_2
**$La_5N_4F_2$**	LaN	LaF_3	La
**$La_5N_4P_2$**	LaP	P_3N_5	LaN
**$La_5N_4S_2$**	$La_4S_3N_2$	N_2	LaN
**$La_5N_4Cl_2$**	La_2NCl_3	LaN	La
**$La_5N_4As_2$**	LaAs	N_2	LaN

**$La_5N_4Se_2$**	$La_4Se_3N_2$	N_2	LaN
**$La_5N_4Br_2$**	La_3Br_6N	La	LaN
**$La_5N_4Sb_2$**	$LaSb_2$	LaN	
**$La_5N_4Te_2$**	$La_4Te_3N_2$	N_2	LaN
**$La_5N_4I_2$**	LaI_2	LaN	
**Ce_2C**	Ce_2C_3	Ce	
**Ce_2CO_2**	Ce_2O_3	Ce_2C_3	
**Ce_2CF_2**	Ce_2C_3	Ce	CeF_3
**Ce_2CP_2**	CeP	C	
**Ce_2CS_2**	C	CeS	
**Ce_2CCl_2**	Ce_3CCl_5	Ce_2C_2Cl	Ce
**Ce_2CAs_2**	CeAs	C	
**Ce_2CSe_2**	C	CeSe	
**Ce_2CBr_2**	Ce_2Br_5	$Ce_3C_2Br_3$	Ce
**Ce_2CSb_2**	CeSb	C	
**Ce_2CTe_2**	CeTe	C	
**Ce_2CI_2**	$Ce_{12}C_6I_{17}$	Ce_2C_3	Ce
**Ce_3C_2**	Ce_2C_3	Ce	
**$Ce_3C_2O_2$**	Ce_2O_3	Ce_2C_3	CeO
**$Ce_3C_2F_2$**	Ce_2C_3	Ce	CeF_3
**$Ce_3C_2P_2$**	Ce_2C_3	CeP	C
**$Ce_3C_2S_2$**	Ce_2C_3	C	CeS
**$Ce_3C_2Cl_2$**	Ce_3CCl_5	Ce_2C_2Cl	Ce
**$Ce_3C_2As_2$**	Ce_2C_3	CeAs	C
**$Ce_3C_2Se_2$**	Ce_2C_3	C	CeSe
**$Ce_3C_2Br_2$**	$Ce_3C_2Br_3$	$Ce_4C_4Br_3$	Ce
**$Ce_3C_2Sb_2$**	Ce_2C_3	CeSb	C
**$Ce_3C_2Te_2$**	CeTe	Ce_2C_3	C
**$Ce_3C_2I_2$**	$Ce_{12}C_6I_{17}$	Ce_2C_3	Ce
**Ce_4C_3**	Ce_2C_3	Ce	
**$Ce_4C_3O_2$**	Ce_2C_3	CeO	
**$Ce_4C_3F_2$**	Ce_2C_3	Ce	CeF_3
**$Ce_4C_3P_2$**	Ce_2C_3	CeP	
**$Ce_4C_3S_2$**	Ce_2C_3	CeS	
**$Ce_4C_3Cl_2$**	Ce_3CCl_5	Ce_2C_2Cl	Ce
**$Ce_4C_3As_2$**	Ce_2C_3	CeAs	
**$Ce_4C_3Se_2$**	Ce_2C_3	CeSe	
**$Ce_4C_3Br_2$**	Ce_2C_3	$Ce_4C_4Br_3$	Ce
**$Ce_4C_3Sb_2$**	Ce_2C_3	CeSb	
**$Ce_4C_3Te_2$**	CeTe	Ce_2C_3	
**$Ce_4C_3I_2$**	$Ce_{12}C_6I_{17}$	Ce_2C_3	Ce

**Ce_5C_4**	Ce_2C_3	Ce	
**$Ce_5C_4O_2$**	Ce_2C_3	Ce	CeO
**$Ce_5C_4F_2$**	Ce_2C_3	Ce	CeF_3
**$Ce_5C_4P_2$**	Ce_2C_3	CeP	Ce
**$Ce_5C_4S_2$**	Ce_2C_3	Ce	CeS
**$Ce_5C_4Cl_2$**	Ce_2C_2Cl	Ce	
**$Ce_5C_4As_2$**	Ce_2C_3	CeAs	Ce
**$Ce_5C_4Se_2$**	Ce_2C_3	Ce	CeSe
**$Ce_5C_4Br_2$**	Ce_2C_3	$Ce_4C_4Br_3$	Ce
**$Ce_5C_4Sb_2$**	Ce_2Sb	Ce_2C_3	CeSb
**$Ce_5C_4Te_2$**	CeTe	Ce_2C_3	Ce
**$Ce_5C_4I_2$**	$Ce_{12}C_6I_{17}$	Ce_2C_3	Ce
**Ce_2N**	CeN	Ce	
**Ce_2NO_2**	CeN_2	Ce_5O_9	CeN
**Ce_2NF_2**	Ce	CeN	CeF_3
**Ce_2NP_2**	CeP	P_3N_5	CeN
**Ce_2NS_2**	Ce_3S_3N	N_2	
**Ce_2NCl_2**	Ce_2NCl_3	Ce	CeN
**Ce_2NAs_2**	$CeAs_2$	CeN	
**Ce_2NSe_2**	$CeSe_2$	$Ce_4Se_3N_2$	N_2
**Ce_2NBr_2**	Ce_2Br_3N	Ce	CeN
**Ce_2NSb_2**	$CeSb_2$	CeN	
**Ce_2NTe_2**	$Ce_4Te_3N_2$	Ce_2Te_5	CeN
**Ce_2NI_2**	CeI_3	Ce	CeN
**Ce_3N_2**	CeN	Ce	
**$Ce_3N_2O_2$**	CeN_2	Ce_5O_9	CeN
**$Ce_3N_2F_2$**	Ce	CeN	CeF_3
**$Ce_3N_2P_2$**	CeP	P_3N_5	CeN
**$Ce_3N_2S_2$**	Ce_3S_3N	CeN_2	CeN
**$Ce_3N_2Cl_2$**	Ce_2NCl_3	Ce	CeN
**$Ce_3N_2As_2$**	$CeAs_2$	CeN	
**$Ce_3N_2Se_2$**	Ce_2SeN_2	$Ce_4Se_3N_2$	
**$Ce_3N_2Br_2$**	Ce_2Br_3N	Ce	CeN
**$Ce_3N_2Sb_2$**	$CeSb_2$	CeN	
**$Ce_3N_2Te_2$**	$Ce_4Te_3N_2$	Ce_2Te_5	CeN
**$Ce_3N_2I_2$**	CeI_3	Ce	CeN
**Ce_4N_3**	CeN	Ce	
**$Ce_4N_3O_2$**	CeN_2	Ce_5O_9	CeN
**$Ce_4N_3F_2$**	Ce	CeN	CeF_3
**$Ce_4N_3P_2$**	CeP	P_3N_5	CeN
**$Ce_4N_3S_2$**	Ce_3S_3N	CeN_2	CeN

**$Ce_4N_3Cl_2$**	Ce_2NCl_3	Ce	CeN
**$Ce_4N_3As_2$**	$CeAs_2$	CeN	
**$Ce_4N_3Se_2$**	Ce_2SeN_2	$Ce_4Se_3N_2$	CeN
**$Ce_4N_3Br_2$**	Ce_2Br_3N	Ce	CeN
**$Ce_4N_3Sb_2$**	$CeSb_2$	CeN	
**$Ce_4N_3Te_2$**	$Ce_4Te_3N_2$	Ce_2Te_5	CeN
**$Ce_4N_3I_2$**	CeI_3	Ce	CeN
**Ce_5N_4**	CeN	Ce	
**$Ce_5N_4O_2$**	CeN_2	Ce_5O_9	CeN
**$Ce_5N_4F_2$**	Ce	CeN	CeF_3
**$Ce_5N_4P_2$**	CeP	P_3N_5	CeN
**$Ce_5N_4S_2$**	Ce_3S_3N	CeN_2	CeN
**$Ce_5N_4Cl_2$**	Ce_2NCl_3	Ce	CeN
**$Ce_5N_4As_2$**	$CeAs_2$	CeN	
**$Ce_5N_4Se_2$**	Ce_2SeN_2	$Ce_4Se_3N_2$	CeN
**$Ce_5N_4Br_2$**	Ce_2Br_3N	Ce	CeN
**$Ce_5N_4Sb_2$**	$CeSb_2$	CeN	
**$Ce_5N_4Te_2$**	$Ce_4Te_3N_2$	Ce_2Te_5	CeN
**$Ce_5N_4I_2$**	CeI_3	Ce	CeN
**Pr_2C**	Pr_2C_3	Pr	
**Pr_2CO_2**	Pr_2O_3	Pr_2C_3	
**Pr_2CF_2**	Pr_2C_3	Pr	PrF_3
**Pr_2CP_2**	PrP	C	
**Pr_2CS_2**	C	PrS	
**Pr_2CCl_2**	$Pr_4C_2Cl_5$	$Pr_8C_8Cl_5$	Pr
**Pr_2CAs_2**	PrAs	C	
**Pr_2CSe_2**	C	PrSe	
**Pr_2CBr_2**	$Pr_4C_2Br_5$	Pr_2CBr	
**Pr_2CSb_2**	PrSb	C	
**Pr_2CTe_2**	PrTe	C	
**Pr_2CI_2**	Pr_2C_3	$Pr_{12}C_6I_{17}$	Pr
**Pr_3C_2**	Pr_2C_3	Pr	
**$Pr_3C_2O_2$**	Pr_2O_3	Pr_2C_3	Pr
**$Pr_3C_2F_2$**	Pr_2C_3	Pr	PrF_3
**$Pr_3C_2P_2$**	Pr_2C_3	PrP	C
**$Pr_3C_2S_2$**	Pr_2C_3	C	PrS
**$Pr_3C_2Cl_2$**	$Pr_4C_2Cl_5$	$Pr_8C_8Cl_5$	Pr
**$Pr_3C_2As_2$**	Pr_2C_3	PrAs	C
**$Pr_3C_2Se_2$**	Pr_2C_3	C	PrSe
**$Pr_3C_2Br_2$**	$Pr_4C_2Br_5$	Pr_2CBr	$Pr_5(C_2Br)_3$
**$Pr_3C_2Sb_2$**	Pr_2C_3	PrSb	C

**$Pr_3C_2Te_2$**	$PrTe$	Pr_2C_3	C
**$Pr_3C_2I_2$**	Pr_2C_3	$Pr_{12}C_6I_{17}$	Pr
**Pr_4C_3**	Pr_2C_3	Pr	
**$Pr_4C_3O_2$**	Pr_2O_3	Pr_2C_3	Pr
**$Pr_4C_3F_2$**	Pr_2C_3	Pr	PrF_3
**$Pr_4C_3P_2$**	Pr_2C_3	PrP	
**$Pr_4C_3S_2$**	Pr_2C_3	PrS	
**$Pr_4C_3Cl_2$**	$Pr_4C_2Cl_5$	$Pr_8C_8Cl_5$	Pr
**$Pr_4C_3As_2$**	Pr_2C_3	$PrAs$	
**$Pr_4C_3Se_2$**	Pr_2C_3	$PrSe$	
**$Pr_4C_3Br_2$**	Pr_2CBr	Pr_2C_3	$Pr_5(C_2Br)_3$
**$Pr_4C_3Sb_2$**	Pr_2C_3	$PrSb$	
**$Pr_4C_3Te_2$**	$PrTe$	Pr_2C_3	
**$Pr_4C_3I_2$**	Pr_2C_3	$Pr_{12}C_6I_{17}$	Pr
**Pr_5C_4**	Pr_2C_3	Pr	
**$Pr_5C_4O_2$**	Pr_2O_3	Pr_2C_3	Pr
**$Pr_5C_4F_2$**	Pr_2C_3	Pr	PrF_3
**$Pr_5C_4P_2$**	Pr_2C_3	PrP	Pr
**$Pr_5C_4S_2$**	Pr_2C_3	Pr	PrS
**$Pr_5C_4Cl_2$**	Pr_2C_3	$Pr_8C_8Cl_5$	Pr
**$Pr_5C_4As_2$**	Pr_2C_3	$PrAs$	Pr
**$Pr_5C_4Se_2$**	Pr_2C_3	Pr	$PrSe$
**$Pr_5C_4Br_2$**	Pr_2CBr	Pr_2C_3	$Pr_5(C_2Br)_3$
**$Pr_5C_4Sb_2$**	Pr_4Sb_3	Pr_2C_3	$PrSb$
**$Pr_5C_4Te_2$**	$PrTe$	Pr_2C_3	Pr
**$Pr_5C_4I_2$**	Pr_2C_3	$Pr_{12}C_6I_{17}$	Pr
**Pr_2N**	PrN	Pr	
**Pr_2NO_2**	PrN	Pr_2O_3	N_2
**Pr_2NF_2**	Pr	PrN	PrF_3
**Pr_2NP_2**	PrP	P_3N_5	PrN
**Pr_2NS_2**	Pr_3S_3N	N_2	
**Pr_2NCl_2**	Pr_2NCl_3	Pr	PrN
**Pr_2NAs_2**	$PrAs$	N_2	
**Pr_2NSe_2**	Pr_3Se_3N	N_2	
**Pr_2NBr_2**	Pr_2Br_5	Pr	PrN
**Pr_2NSb_2**	$PrSb_2$	PrN	
**Pr_2NTe_2**	$Pr_4Te_3N_2$	$PrTe_3$	N_2
**Pr_2NI_2**	PrI_2	PrN	
**Pr_3N_2**	PrN	Pr	
**$Pr_3N_2O_2$**	PrN	Pr_2O_3	N_2
**$Pr_3N_2F_2$**	Pr	PrN	PrF_3

**$Pr_3N_2P_2$**	PrP	P_3N_5	PrN
**$Pr_3N_2S_2$**	$Pr_4S_3N_2$	N_2	PrN
**$Pr_3N_2Cl_2$**	Pr_2NCl_3	Pr	PrN
**$Pr_3N_2As_2$**	$PrAs$	N_2	PrN
**$Pr_3N_2Se_2$**	$Pr_4Se_3N_2$	N_2	PrN
**$Pr_3N_2Br_2$**	Pr_2Br_5	Pr	PrN
**$Pr_3N_2Sb_2$**	$PrSb_2$	PrN	
**$Pr_3N_2Te_2$**	$Pr_4Te_3N_2$	N_2	PrN
**$Pr_3N_2I_2$**	PrI_2	PrN	
**Pr_4N_3**	PrN	Pr	
**$Pr_4N_3O_2$**	PrN	Pr_2O_3	N_2
**$Pr_4N_3F_2$**	Pr	PrN	PrF_3
**$Pr_4N_3P_2$**	PrP	P_3N_5	PrN
**$Pr_4N_3S_2$**	$Pr_4S_3N_2$	N_2	PrN
**$Pr_4N_3Cl_2$**	Pr_2NCl_3	Pr	PrN
**$Pr_4N_3As_2$**	$PrAs$	N_2	PrN
**$Pr_4N_3Se_2$**	$Pr_4Se_3N_2$	N_2	PrN
**$Pr_4N_3Br_2$**	Pr_2Br_5	Pr	PrN
**$Pr_4N_3Sb_2$**	$PrSb_2$	PrN	
**$Pr_4N_3Te_2$**	$Pr_4Te_3N_2$	N_2	PrN
**$Pr_4N_3I_2$**	PrI_2	PrN	
**Pr_5N_4**	PrN	Pr	
**$Pr_5N_4O_2$**	PrN	Pr_2O_3	N_2
**$Pr_5N_4F_2$**	Pr	PrN	PrF_3
**$Pr_5N_4P_2$**	PrP	P_3N_5	PrN
**$Pr_5N_4S_2$**	$Pr_4S_3N_2$	N_2	PrN
**$Pr_5N_4Cl_2$**	Pr_2NCl_3	Pr	PrN
**$Pr_5N_4As_2$**	$PrAs$	N_2	PrN
**$Pr_5N_4Se_2$**	$Pr_4Se_3N_2$	N_2	PrN
**$Pr_5N_4Br_2$**	Pr_2Br_5	Pr	PrN
**$Pr_5N_4Sb_2$**	$PrSb_2$	PrN	
**$Pr_5N_4Te_2$**	$Pr_4Te_3N_2$	N_2	PrN
**$Pr_5N_4I_2$**	PrI_2	PrN	
**Nd_2C**	Nd_2C_3	Nd	
**Nd_2CO_2**	Nd_2O_3	Nd_2C_3	
**Nd_2CF_2**	Nd_2C_3	Nd	NdF_3
**Nd_2CP_2**	NdP	C	
**Nd_2CS_2**	C	NdS	
**Nd_2CCl_2**	Nd_2C_3	$NdCl_3$	Nd
**Nd_2CAs_2**	$NdAs$	C	
**Nd_2CSe_2**	C	$NdSe$	

**Nd_2CBr_2**	Nd_2C_3	$NdBr_3$	Nd
**Nd_2CSb_2**	NdSb	C	
**Nd_2CTe_2**	NdTe	C	
**Nd_2CI_2**	Nd_2C_3	$Nd_{12}C_6I_{17}$	Nd
**Nd_3C_2**	Nd_2C_3	Nd	
**$Nd_3C_2O_2$**	Nd_2O_3	Nd_2C_3	Nd
**$Nd_3C_2F_2$**	Nd_2C_3	Nd	NdF_3
**$Nd_3C_2P_2$**	Nd_2C_3	NdP	C
**$Nd_3C_2S_2$**	Nd_2C_3	C	NdS
**$Nd_3C_2Cl_2$**	Nd_2C_3	$NdCl_3$	Nd
**$Nd_3C_2As_2$**	Nd_2C_3	NdAs	C
**$Nd_3C_2Se_2$**	Nd_2C_3	C	NdSe
**$Nd_3C_2Br_2$**	Nd_2C_3	$NdBr_3$	Nd
**$Nd_3C_2Sb_2$**	Nd_2C_3	NdSb	C
**$Nd_3C_2Te_2$**	NdTe	Nd_2C_3	C
**$Nd_3C_2I_2$**	Nd_2C_3	$Nd_{12}C_6I_{17}$	Nd
**Nd_4C_3**	Nd_2C_3	Nd	
**$Nd_4C_3O_2$**	Nd_2O_3	Nd_2C_3	Nd
**$Nd_4C_3F_2$**	Nd_2C_3	Nd	NdF_3
**$Nd_4C_3P_2$**	Nd_2C_3	NdP	
**$Nd_4C_3S_2$**	Nd_2C_3	NdS	
**$Nd_4C_3Cl_2$**	Nd_2C_3	$NdCl_3$	Nd
**$Nd_4C_3As_2$**	Nd_2C_3	NdAs	
**$Nd_4C_3Se_2$**	Nd_2C_3	NdSe	
**$Nd_4C_3Br_2$**	Nd_2C_3	$NdBr_3$	Nd
**$Nd_4C_3Sb_2$**	Nd_2C_3	NdSb	
**$Nd_4C_3Te_2$**	NdTe	Nd_2C_3	
**$Nd_4C_3I_2$**	Nd_2C_3	$Nd_{12}C_6I_{17}$	Nd
**Nd_5C_4**	Nd_2C_3	Nd	
**$Nd_5C_4O_2$**	Nd_2O_3	Nd_2C_3	Nd
**$Nd_5C_4F_2$**	Nd_2C_3	Nd	NdF_3
**$Nd_5C_4P_2$**	Nd_2C_3	NdP	Nd
**$Nd_5C_4S_2$**	Nd_2C_3	Nd	NdS
**$Nd_5C_4Cl_2$**	Nd_2C_3	$NdCl_3$	Nd
**$Nd_5C_4As_2$**	Nd_2C_3	NdAs	Nd
**$Nd_5C_4Se_2$**	Nd_2C_3	Nd	NdSe
**$Nd_5C_4Br_2$**	Nd_2C_3	$NdBr_3$	Nd
**$Nd_5C_4Sb_2$**	Nd_4Sb_3	Nd_2C_3	NdSb
**$Nd_5C_4Te_2$**	NdTe	Nd_2C_3	Nd
**$Nd_5C_4I_2$**	Nd_2C_3	$Nd_{12}C_6I_{17}$	Nd
**Nd_2N**	NdN	Nd	

**Nd_2NO_2**	NdN	Nd_2O_3	N_2
**Nd_2NF_2**	Nd	NdN	NdF_3
**Nd_2NP_2**	NdP	P_3N_5	NdN
**Nd_2NS_2**	Nd_3S_3N	N_2	
**Nd_2NCl_2**	Nd_2NCl_3	NdN	Nd
**Nd_2NAs_2**	NdAs	As	NdN
**Nd_2NSe_2**	Nd_3Se_3N	N_2	
**Nd_2NBr_2**	$NdBr_3$	Nd	NdN
**Nd_2NSb_2**	$NdSb_2$	NdN	
**Nd_2NTe_2**	$Nd_4Te_3N_2$	$NdTe_3$	N_2
**Nd_2NI_2**	NdI_2	NdN	
**Nd_3N_2**	NdN	Nd	
**$Nd_3N_2O_2$**	NdN	Nd_2O_3	N_2
**$Nd_3N_2F_2$**	Nd	NdN	NdF_3
**$Nd_3N_2P_2$**	NdP	P_3N_5	NdN
**$Nd_3N_2S_2$**	Nd_3S_3N	N_2	NdN
**$Nd_3N_2Cl_2$**	Nd_2NCl_3	NdN	Nd
**$Nd_3N_2As_2$**	NdAs	As	NdN
**$Nd_3N_2Se_2$**	$Nd_4Se_3N_2$	N_2	NdN
**$Nd_3N_2Br_2$**	$NdBr_3$	Nd	NdN
**$Nd_3N_2Sb_2$**	$NdSb_2$	NdN	
**$Nd_3N_2Te_2$**	$Nd_4Te_3N_2$	N_2	NdN
**$Nd_3N_2I_2$**	NdI_2	NdN	
**Nd_4N_3**	NdN	Nd	
**$Nd_4N_3O_2$**	NdN	Nd_2O_3	N_2
**$Nd_4N_3F_2$**	Nd	NdN	NdF_3
**$Nd_4N_3P_2$**	NdP	P_3N_5	NdN
**$Nd_4N_3S_2$**	Nd_3S_3N	N_2	NdN
**$Nd_4N_3Cl_2$**	Nd_2NCl_3	NdN	Nd
**$Nd_4N_3As_2$**	NdAs	As	NdN
**$Nd_4N_3Se_2$**	$Nd_4Se_3N_2$	N_2	NdN
**$Nd_4N_3Br_2$**	$NdBr_3$	Nd	NdN
**$Nd_4N_3Sb_2$**	$NdSb_2$	NdN	
**$Nd_4N_3Te_2$**	$Nd_4Te_3N_2$	N_2	NdN
**$Nd_4N_3I_2$**	NdI_2	NdN	
**Nd_5N_4**	NdN	Nd	
**$Nd_5N_4O_2$**	NdN	Nd_2O_3	N_2
**$Nd_5N_4F_2$**	Nd	NdN	NdF_3
**$Nd_5N_4P_2$**	NdP	P_3N_5	NdN
**$Nd_5N_4S_2$**	Nd_3S_3N	N_2	NdN
**$Nd_5N_4Cl_2$**	Nd_2NCl_3	NdN	Nd

**$Nd_5N_4As_2$**	NdAs	As	NdN
**$Nd_5N_4Se_2$**	$Nd_4Se_3N_2$	N_2	NdN
**$Nd_5N_4Br_2$**	$NdBr_3$	Nd	NdN
**$Nd_5N_4Sb_2$**	$NdSb_2$	NdN	
**$Nd_5N_4Te_2$**	$Nd_4Te_3N_2$	N_2	NdN
**$Nd_5N_4I_2$**	NdI_2	NdN	
**Sm_2C**	Sm_2C		
**Sm_2CO_2**	Sm_2O_3	Sm_2C_3	
**Sm_2CF_2**	Sm_2C	SmF_3	Sm_2C_3
**Sm_2CP_2**	SmP	C	
**Sm_2CS_2**	C	SmS	
**Sm_2CCl_2**	Sm_2C	Sm_2C_3	$SmCl_3$
**Sm_2CAs_2**	SmAs	C	
**Sm_2CSe_2**	C	SmSe	
**Sm_2CBr_2**	Sm_2C	Sm_2C_3	$SmBr_3$
**Sm_2CSb_2**	SmSb	C	
**Sm_2CTe_2**	SmTe	C	
**Sm_2CI_2**	Sm_2C	Sm_2C_3	SmI_3
**Sm_3C_2**	Sm_2C	Sm_2C_3	
**$Sm_3C_2O_2$**	Sm_2C	Sm_2O_3	Sm_2C_3
**$Sm_3C_2F_2$**	Sm_2C	SmF_3	Sm_2C_3
**$Sm_3C_2P_2$**	Sm_2C_3	SmP	C
**$Sm_3C_2S_2$**	Sm_2C_3	C	SmS
**$Sm_3C_2Cl_2$**	Sm_2C	Sm_2C_3	$SmCl_3$
**$Sm_3C_2As_2$**	Sm_2C_3	SmAs	C
**$Sm_3C_2Se_2$**	Sm_2C_3	C	SmSe
**$Sm_3C_2Br_2$**	Sm_2C	Sm_2C_3	$SmBr_3$
**$Sm_3C_2Sb_2$**	Sm_2C_3	SmSb	C
**$Sm_3C_2Te_2$**	SmTe	Sm_2C_3	C
**$Sm_3C_2I_2$**	Sm_2C	Sm_2C_3	SmI_3
**Sm_4C_3**	Sm_2C	Sm_2C_3	
**$Sm_4C_3O_2$**	Sm_2C	Sm_2O_3	Sm_2C_3
**$Sm_4C_3F_2$**	Sm_2C	SmF_3	Sm_2C_3
**$Sm_4C_3P_2$**	Sm_2C_3	SmP	
**$Sm_4C_3S_2$**	Sm_2C_3	SmS	
**$Sm_4C_3Cl_2$**	Sm_2C	Sm_2C_3	$SmCl_3$
**$Sm_4C_3As_2$**	Sm_2C_3	SmAs	
**$Sm_4C_3Se_2$**	Sm_2C_3	SmSe	
**$Sm_4C_3Br_2$**	Sm_2C	Sm_2C_3	$SmBr_3$
**$Sm_4C_3Sb_2$**	Sm_2C_3	SmSb	
**$Sm_4C_3Te_2$**	SmTe	Sm_2C_3	

**$Sm_4C_3I_2$**	Sm_2C	Sm_2C_3	SmI_3
**Sm_5C_4**	Sm_2C	Sm_2C_3	
**$Sm_5C_4O_2$**	Sm_2C	Sm_2O_3	Sm_2C_3
**$Sm_5C_4F_2$**	Sm_2C	SmF_3	Sm_2C_3
**$Sm_5C_4P_2$**	Sm_2C	Sm_2C_3	SmP
**$Sm_5C_4S_2$**	Sm_2C	Sm_2C_3	SmS
**$Sm_5C_4Cl_2$**	Sm_2C	Sm_2C_3	$SmCl_3$
**$Sm_5C_4As_2$**	Sm_2C	Sm_2C_3	SmAs
**$Sm_5C_4Se_2$**	Sm_2C	Sm_2C_3	SmSe
**$Sm_5C_4Br_2$**	Sm_2C	Sm_2C_3	$SmBr_3$
**$Sm_5C_4Sb_2$**	Sm_5Sb_3	Sm_2C_3	SmSb
**$Sm_5C_4Te_2$**	Sm_2C	SmTe	Sm_2C_3
**$Sm_5C_4I_2$**	Sm_2C	Sm_2C_3	SmI_3
**Sm_2N**	SmN	Sm	
**Sm_2NO_2**	SmN	Sm_2O_3	N_2
**Sm_2NF_2**	SmN	SmF_3	Sm
**Sm_2NP_2**	SmP	P_3N_5	SmN
**Sm_2NS_2**	$Sm_4S_3N_2$	Sm_2S_3	N_2
**Sm_2NCl_2**	SmN	$SmCl_3$	Sm
**Sm_2NAs_2**	SmAs	As	SmN
**Sm_2NSe_2**	Sm_2Se_3	N_2	SmN
**Sm_2NBr_2**	$SmBr_3$	Sm	SmN
**Sm_2NSb_2**	Sm_2Sb_5	SmSb	SmN
**Sm_2NTe_2**	Sm_2Te_3	$SmTe_3$	SmN
**Sm_2NI_2**	SmI_3	Sm	SmN
**Sm_3N_2**	SmN	Sm	
**$Sm_3N_2O_2$**	SmN	Sm_2O_3	N_2
**$Sm_3N_2F_2$**	SmN	SmF_3	Sm
**$Sm_3N_2P_2$**	SmP	P_3N_5	SmN
**$Sm_3N_2S_2$**	$Sm_4S_3N_2$	N_2	SmN
**$Sm_3N_2Cl_2$**	SmN	$SmCl_3$	Sm
**$Sm_3N_2As_2$**	SmAs	As	SmN
**$Sm_3N_2Se_2$**	Sm_2Se_3	N_2	SmN
**$Sm_3N_2Br_2$**	$SmBr_3$	Sm	SmN
**$Sm_3N_2Sb_2$**	Sm_2Sb_5	SmSb	SmN
**$Sm_3N_2Te_2$**	Sm_2Te_3	$SmTe_3$	SmN
**$Sm_3N_2I_2$**	SmI_3	Sm	SmN
**Sm_4N_3**	SmN	Sm	
**$Sm_4N_3O_2$**	SmN	Sm_2O_3	N_2
**$Sm_4N_3F_2$**	SmN	SmF_3	Sm
**$Sm_4N_3P_2$**	SmP	P_3N_5	SmN

**$Sm_4N_3S_2$**	$Sm_4S_3N_2$	N_2	SmN
**$Sm_4N_3Cl_2$**	SmN	$SmCl_3$	Sm
**$Sm_4N_3As_2$**	SmAs	As	SmN
**$Sm_4N_3Se_2$**	Sm_2Se_3	N_2	SmN
**$Sm_4N_3Br_2$**	$SmBr_3$	Sm	SmN
**$Sm_4N_3Sb_2$**	Sm_2Sb_5	SmSb	SmN
**$Sm_4N_3Te_2$**	Sm_2Te_3	$SmTe_3$	SmN
**$Sm_4N_3I_2$**	SmI_3	Sm	SmN
**Sm_5N_4**	SmN	Sm	
**$Sm_5N_4O_2$**	SmN	Sm_2O_3	N_2
**$Sm_5N_4F_2$**	SmN	SmF_3	Sm
**$Sm_5N_4P_2$**	SmP	P_3N_5	SmN
**$Sm_5N_4S_2$**	$Sm_4S_3N_2$	N_2	SmN
**$Sm_5N_4Cl_2$**	SmN	$SmCl_3$	Sm
**$Sm_5N_4As_2$**	SmAs	As	SmN
**$Sm_5N_4Se_2$**	Sm_2Se_3	N_2	SmN
**$Sm_5N_4Br_2$**	$SmBr_3$	Sm	SmN
**$Sm_5N_4Sb_2$**	Sm_2Sb_5	SmSb	SmN
**$Sm_5N_4Te_2$**	Sm_2Te_3	$SmTe_3$	SmN
**$Sm_5N_4I_2$**	SmI_3	Sm	SmN
**Eu_2C**	EuC_6	Eu	
**Eu_2CO_2**	C	EuO	
**Eu_2CF_2**	EuC_6	Eu	EuF_2
**Eu_2CP_2**	EuP	C	
**Eu_2CS_2**	C	EuS	
**Eu_2CCl_2**	EuC_6	Eu	$EuCl_2$
**Eu_2CAs_2**	EuAs	C	
**Eu_2CSe_2**	C	EuSe	
**Eu_2CBr_2**	EuC_6	Eu	$EuBr_2$
**Eu_2CSb_2**	Eu_2Sb_3	$Eu_{16}Sb_{11}$	C
**Eu_2CTe_2**	EuTe	C	
**Eu_2CI_2**	EuC_6	Eu	EuI_2
**Eu_3C_2**	EuC_6	Eu	
**$Eu_3C_2O_2$**	EuC_6	Eu	EuO
**$Eu_3C_2F_2$**	EuC_6	Eu	EuF_2
**$Eu_3C_2P_2$**	EuC_6	EuP	Eu
**$Eu_3C_2S_2$**	EuC_6	Eu	EuS
**$Eu_3C_2Cl_2$**	EuC_6	Eu	$EuCl_2$
**$Eu_3C_2As_2$**	EuC_6	Eu_4As_3	
**$Eu_3C_2Se_2$**	EuC_6	Eu	EuSe
**$Eu_3C_2Br_2$**	EuC_6	Eu	$EuBr_2$

**$Eu_3C_2Sb_2$**	Eu_5Sb_3	$Eu_{16}Sb_{11}$	C
**$Eu_3C_2Te_2$**	EuC_6	EuTe	Eu
**$Eu_3C_2I_2$**	EuC_6	Eu	EuI_2
**Eu_4C_3**	EuC_6	Eu	
**$Eu_4C_3O_2$**	EuC_6	Eu	EuO
**$Eu_4C_3F_2$**	EuC_6	Eu	EuF_2
**$Eu_4C_3P_2$**	EuC_6	EuP	Eu
**$Eu_4C_3S_2$**	EuC_6	Eu	EuS
**$Eu_4C_3Cl_2$**	EuC_6	Eu	$EuCl_2$
**$Eu_4C_3As_2$**	EuC_6	Eu_5As_3	Eu
**$Eu_4C_3Se_2$**	EuC_6	Eu	EuSe
**$Eu_4C_3Br_2$**	EuC_6	Eu	$EuBr_2$
**$Eu_4C_3Sb_2$**	EuC_6	Eu_5Sb_3	Eu
**$Eu_4C_3Te_2$**	EuC_6	EuTe	Eu
**$Eu_4C_3I_2$**	EuC_6	Eu	EuI_2
**Eu_5C_4**	EuC_6	Eu	
**$Eu_5C_4O_2$**	EuC_6	Eu	EuO
**$Eu_5C_4F_2$**	EuC_6	Eu	EuF_2
**$Eu_5C_4P_2$**	EuC_6	EuP	Eu
**$Eu_5C_4S_2$**	EuC_6	Eu	EuS
**$Eu_5C_4Cl_2$**	EuC_6	Eu	$EuCl_2$
**$Eu_5C_4As_2$**	EuC_6	Eu_5As_3	Eu
**$Eu_5C_4Se_2$**	EuC_6	Eu	EuSe
**$Eu_5C_4Br_2$**	EuC_6	Eu	$EuBr_2$
**$Eu_5C_4Sb_2$**	EuC_6	Eu_5Sb_3	Eu
**$Eu_5C_4Te_2$**	EuC_6	EuTe	Eu
**$Eu_5C_4I_2$**	EuC_6	Eu	EuI_2
**Eu_2N**	Eu_2N		
**Eu_2NO_2**	N_2	EuO	
**Eu_2NF_2**	EuN	EuF_2	
**Eu_2NP_2**	Eu_3P_4	P_3N_5	EuN
**Eu_2NS_2**	EuS	N_2	
**Eu_2NCl_2**	EuN	$EuCl_2$	
**Eu_2NAs_2**	EuAs	N_2	
**Eu_2NSe_2**	EuSe	N_2	
**Eu_2NBr_2**	$EuBr_2$	EuN	
**Eu_2NSb_2**	$EuSb_2$	EuN	
**Eu_2NTe_2**	EuTe	N_2	
**Eu_2NI_2**	EuI_2	EuN	
**Eu_3N_2**	Eu_2N	EuN	
**$Eu_3N_2O_2$**	EuN	N_2	EuO

**$Eu_3N_2F_2$**	EuN	EuF_2	
**$Eu_3N_2P_2$**	Eu_3P_4	P_3N_5	EuN
**$Eu_3N_2S_2$**	EuS	N_2	EuN
**$Eu_3N_2Cl_2$**	EuN	$EuCl_2$	
**$Eu_3N_2As_2$**	EuAs	N_2	EuN
**$Eu_3N_2Se_2$**	EuSe	N_2	EuN
**$Eu_3N_2Br_2$**	$EuBr_2$	EuN	
**$Eu_3N_2Sb_2$**	$EuSb_2$	EuN	
**$Eu_3N_2Te_2$**	EuTe	N_2	EuN
**$Eu_3N_2I_2$**	EuI_2	EuN	
**Eu_4N_3**	Eu_2N	EuN	
**$Eu_4N_3O_2$**	EuN	N_2	EuO
**$Eu_4N_3F_2$**	EuN	EuF_2	
**$Eu_4N_3P_2$**	Eu_3P_4	P_3N_5	EuN
**$Eu_4N_3S_2$**	EuS	N_2	EuN
**$Eu_4N_3Cl_2$**	EuN	$EuCl_2$	
**$Eu_4N_3As_2$**	EuAs	N_2	EuN
**$Eu_4N_3Se_2$**	EuSe	N_2	EuN
**$Eu_4N_3Br_2$**	$EuBr_2$	EuN	
**$Eu_4N_3Sb_2$**	$EuSb_2$	EuN	
**$Eu_4N_3Te_2$**	EuTe	N_2	EuN
**$Eu_4N_3I_2$**	EuI_2	EuN	
**Eu_5N_4**	Eu_2N	EuN	
**$Eu_5N_4O_2$**	EuN	N_2	EuO
**$Eu_5N_4F_2$**	EuN	EuF_2	
**$Eu_5N_4P_2$**	Eu_3P_4	P_3N_5	EuN
**$Eu_5N_4S_2$**	EuS	N_2	EuN
**$Eu_5N_4Cl_2$**	EuN	$EuCl_2$	
**$Eu_5N_4As_2$**	EuAs	N_2	EuN
**$Eu_5N_4Se_2$**	EuSe	N_2	EuN
**$Eu_5N_4Br_2$**	$EuBr_2$	EuN	
**$Eu_5N_4Sb_2$**	$EuSb_2$	EuN	
**$Eu_5N_4Te_2$**	EuTe	N_2	EuN
**$Eu_5N_4I_2$**	EuI_2	EuN	
**Gd_2C**	Gd_2C		
**Gd_2CO_2**	Gd_2C_3	Gd_2O_3	
**Gd_2CF_2**	Gd_2CF_2		
**Gd_2CP_2**	GdP	C	
**Gd_2CS_2**	C	GdS	
**Gd_2CCl_2**	$Gd_4C_2Cl_3$	$Gd_5(C_2Cl)_3$	$Gd_5C_2Cl_9$
**Gd_2CAs_2**	GdAs	C	

**Gd_2CSe_2**	C	GdSe	
**Gd_2CBr_2**	GdCBr	Gd_2CBr	$GdBr_3$
**Gd_2CSb_2**	GdSb	C	
**Gd_2CTe_2**	GdTe	C	
**Gd_2CI_2**	Gd_3CI_3	C	
**Gd_3C_2**	Gd_2C_3	Gd_2C	
**$Gd_3C_2O_2$**	Gd_2C_3	Gd_2O_3	Gd_2C
**$Gd_3C_2F_2$**	Gd_2C_3	Gd_2C	Gd_2CF_2
**$Gd_3C_2P_2$**	Gd_2C_3	GdP	C
**$Gd_3C_2S_2$**	Gd_2C_3	C	GdS
**$Gd_3C_2Cl_2$**	$Gd_4C_2Cl_3$	$Gd_5(C_2Cl)_3$	Gd_2CCl
**$Gd_3C_2As_2$**	Gd_2C_3	GdAs	C
**$Gd_3C_2Se_2$**	Gd_2C_3	C	GdSe
**$Gd_3C_2Br_2$**	GdCBr	Gd_2CBr	
**$Gd_3C_2Sb_2$**	Gd_2C_3	GdSb	C
**$Gd_3C_2Te_2$**	Gd_2C_3	GdTe	C
**$Gd_3C_2I_2$**	Gd_2CI	Gd_3CI_3	C
**Gd_4C_3**	Gd_2C_3	Gd_2C	
**$Gd_4C_3O_2$**	Gd_2C_3	Gd_2O_3	Gd_2C
**$Gd_4C_3F_2$**	Gd_2C_3	Gd_2C	Gd_2CF_2
**$Gd_4C_3P_2$**	Gd_2C_3	GdP	
**$Gd_4C_3S_2$**	Gd_2C_3	GdS	
**$Gd_4C_3Cl_2$**	$Gd_5(C_2Cl)_3$	Gd_2C_3	Gd_2CCl
**$Gd_4C_3As_2$**	Gd_2C_3	GdAs	
**$Gd_4C_3Se_2$**	Gd_2C_3	GdSe	
**$Gd_4C_3Br_2$**	Gd_2C_3	GdCBr	Gd_2CBr
**$Gd_4C_3Sb_2$**	Gd_2C_3	GdSb	
**$Gd_4C_3Te_2$**	Gd_2C_3	GdTe	
**$Gd_4C_3I_2$**	Gd_2CI	C	
**Gd_5C_4**	Gd_2C_3	Gd_2C	
**$Gd_5C_4O_2$**	Gd_2C_3	Gd_2O_3	Gd_2C
**$Gd_5C_4F_2$**	Gd_2C_3	Gd_2C	Gd_2CF_2
**$Gd_5C_4P_2$**	Gd_2C_3	Gd_2C	GdP
**$Gd_5C_4S_2$**	Gd_2C_3	Gd_2C	GdS
**$Gd_5C_4Cl_2$**	$Gd_5(C_2Cl)_3$	Gd_2C_3	Gd_2CCl
**$Gd_5C_4As_2$**	Gd_2C_3	Gd_2C	GdAs
**$Gd_5C_4Se_2$**	Gd_2C_3	Gd_2C	GdSe
**$Gd_5C_4Br_2$**	Gd_2C_3	GdCBr	Gd_2CBr
**$Gd_5C_4Sb_2$**	Gd_4Sb_3	Gd_2C_3	GdSb
**$Gd_5C_4Te_2$**	Gd_2C_3	Gd_2C	GdTe
**$Gd_5C_4I_2$**	Gd_2CI	Gd_2C_3	C

**Gd_2N**	GdN	Gd	
**Gd_2NO_2**	GdN	Gd_2O_3	N_2
**Gd_2NF_2**	GdN	Gd	GdF_3
**Gd_2NP_2**	GdP	P_3N_5	GdN
**Gd_2NS_2**	Gd_3S_3N	N_2	
**Gd_2NCl_2**	Gd_2NCl_3	GdN	Gd
**Gd_2NAs_2**	$GdAs$	As	GdN
**Gd_2NSe_2**	Gd_3Se_3N	N_2	
**Gd_2NBr_2**	Gd_2Br_3N	Gd	GdN
**Gd_2NSb_2**	$GdSb$	Sb	GdN
**Gd_2NTe_2**	$GdTe_2$	GdN	
**Gd_2NI_2**	Gd_3I	I	GdN
**Gd_3N_2**	GdN	Gd	
**$Gd_3N_2O_2$**	GdN	Gd_2O_3	N_2
**$Gd_3N_2F_2$**	GdN	Gd	GdF_3
**$Gd_3N_2P_2$**	GdP	P_3N_5	GdN
**$Gd_3N_2S_2$**	Gd_3S_3N	N_2	GdN
**$Gd_3N_2Cl_2$**	Gd_2NCl_3	GdN	Gd
**$Gd_3N_2As_2$**	$GdAs$	As	GdN
**$Gd_3N_2Se_2$**	Gd_3Se_3N	N_2	GdN
**$Gd_3N_2Br_2$**	Gd_2Br_3N	Gd	GdN
**$Gd_3N_2Sb_2$**	$GdSb$	Sb	GdN
**$Gd_3N_2Te_2$**	$GdTe_2$	GdN	
**$Gd_3N_2I_2$**	Gd_3I	I	GdN
**Gd_4N_3**	GdN	Gd	
**$Gd_4N_3O_2$**	GdN	Gd_2O_3	N_2
**$Gd_4N_3F_2$**	GdN	Gd	GdF_3
**$Gd_4N_3P_2$**	GdP	P_3N_5	GdN
**$Gd_4N_3S_2$**	Gd_3S_3N	N_2	GdN
**$Gd_4N_3Cl_2$**	Gd_2NCl_3	GdN	Gd
**$Gd_4N_3As_2$**	$GdAs$	As	GdN
**$Gd_4N_3Se_2$**	Gd_3Se_3N	N_2	GdN
**$Gd_4N_3Br_2$**	Gd_2Br_3N	Gd	GdN
**$Gd_4N_3Sb_2$**	$GdSb$	Sb	GdN
**$Gd_4N_3Te_2$**	$GdTe_2$	GdN	
**$Gd_4N_3I_2$**	Gd_3I	I	GdN
**Gd_5N_4**	GdN	Gd	
**$Gd_5N_4O_2$**	GdN	Gd_2O_3	N_2
**$Gd_5N_4F_2$**	GdN	Gd	GdF_3
**$Gd_5N_4P_2$**	GdP	P_3N_5	GdN
**$Gd_5N_4S_2$**	Gd_3S_3N	N_2	GdN

**$Gd_5N_4Cl_2$**	Gd_2NCl_3	GdN	Gd
**$Gd_5N_4As_2$**	GdAs	As	GdN
**$Gd_5N_4Se_2$**	Gd_3Se_3N	N_2	GdN
**$Gd_5N_4Br_2$**	Gd_2Br_3N	Gd	GdN
**$Gd_5N_4Sb_2$**	GdSb	Sb	GdN
**$Gd_5N_4Te_2$**	$GdTe_2$	GdN	
**$Gd_5N_4I_2$**	Gd_3I	I	GdN
**Tb_2C**	Tb_2C		
**Tb_2CO_2**	Tb_2O_3	Tb_4C_5	C
**Tb_2CF_2**	Tb_2C	Tb_4C_5	TbF_3
**Tb_2CP_2**	TbP	C	
**Tb_2CS_2**	C	TbS	
**Tb_2CCl_2**	Tb_2C	Tb_4C_5	$TbCl_3$
**Tb_2CAs_2**	TbAs	C	
**Tb_2CSe_2**	C	TbSe	
**Tb_2CBr_2**	TbCBr	$Tb_4C_2Br_3$	$Tb_5C_2Br_9$
**Tb_2CSb_2**	TbSb	C	
**Tb_2CTe_2**	TbTe	C	
**Tb_2CI_2**	Tb_2CI	C	TbI_3
**Tb_3C_2**	Tb_2C	Tb_4C_5	
**$Tb_3C_2O_2$**	Tb_2C	Tb_2O_3	Tb_4C_5
**$Tb_3C_2F_2$**	Tb_2C	Tb_4C_5	TbF_3
**$Tb_3C_2P_2$**	Tb_4C_5	TbP	C
**$Tb_3C_2S_2$**	Tb_4C_5	C	TbS
**$Tb_3C_2Cl_2$**	Tb_2C	Tb_4C_5	$TbCl_3$
**$Tb_3C_2As_2$**	Tb_4C_5	TbAs	C
**$Tb_3C_2Se_2$**	Tb_4C_5	C	TbSe
**$Tb_3C_2Br_2$**	TbCBr	$Tb_4C_2Br_3$	Tb_4C_5
**$Tb_3C_2Sb_2$**	Tb_4C_5	TbSb	C
**$Tb_3C_2Te_2$**	TbTe	Tb_4C_5	C
**$Tb_3C_2I_2$**	Tb_2CI	C	TbI_3
**Tb_4C_3**	Tb_2C	Tb_4C_5	
**$Tb_4C_3O_2$**	Tb_2C	Tb_2O_3	Tb_4C_5
**$Tb_4C_3F_2$**	Tb_2C	Tb_4C_5	TbF_3
**$Tb_4C_3P_2$**	Tb_4C_5	TbP	C
**$Tb_4C_3S_2$**	Tb_4C_5	C	TbS
**$Tb_4C_3Cl_2$**	Tb_2C	Tb_4C_5	$TbCl_3$
**$Tb_4C_3As_2$**	Tb_4C_5	TbAs	C
**$Tb_4C_3Se_2$**	Tb_4C_5	C	TbSe
**$Tb_4C_3Br_2$**	$Tb_4C_2Br_3$	Tb_4C_5	
**$Tb_4C_3Sb_2$**	Tb_4C_5	TbSb	C

**$Tb_4C_3Te_2$**	TbTe	Tb_4C_5	C
**$Tb_4C_3I_2$**	Tb_2CI	C	
**Tb_5C_4**	Tb_2C	Tb_4C_5	
**$Tb_5C_4O_2$**	Tb_2C	Tb_2O_3	Tb_4C_5
**$Tb_5C_4F_2$**	Tb_2C	Tb_4C_5	TbF_3
**$Tb_5C_4P_2$**	Tb_4C_5	TbP	C
**$Tb_5C_4S_2$**	Tb_4C_5	C	TbS
**$Tb_5C_4Cl_2$**	Tb_2C	Tb_4C_5	$TbCl_3$
**$Tb_5C_4As_2$**	Tb_4C_5	TbAs	C
**$Tb_5C_4Se_2$**	Tb_4C_5	C	TbSe
**$Tb_5C_4Br_2$**	$Tb_4C_2Br_3$	Tb_4C_5	Tb_2CBr
**$Tb_5C_4Sb_2$**	Tb_4C_5	TbSb	C
**$Tb_5C_4Te_2$**	TbTe	Tb_4C_5	C
**$Tb_5C_4I_2$**	Tb_4C_5	Tb_2CI	C
**Tb_2N**	TbN	Tb	
**Tb_2NO_2**	Tb_2O_3	TbN	N_2
**Tb_2NF_2**	Tb	TbN	TbF_3
**Tb_2NP_2**	TbP	P_3N_5	TbN
**Tb_2NS_2**	Tb_3S_3N	N_2	
**Tb_2NCl_2**	Tb_2Cl_3	TbN	$TbCl_3$
**Tb_2NAs_2**	TbAs	As	TbN
**Tb_2NSe_2**	Tb_2Se_3	$Tb_4Se_3N_2$	N_2
**Tb_2NBr_2**	$TbBr_3$	Tb	TbN
**Tb_2NSb_2**	Tb_2Sb_5	TbSb	TbN
**Tb_2NTe_2**	$Tb_4Te_3N_2$	$TbTe_3$	TbN
**Tb_2NI_2**	TbI_3	Tb	TbN
**Tb_3N_2**	TbN	Tb	
**$Tb_3N_2O_2$**	Tb_2O_3	TbN	N_2
**$Tb_3N_2F_2$**	Tb	TbN	TbF_3
**$Tb_3N_2P_2$**	TbP	P_3N_5	TbN
**$Tb_3N_2S_2$**	Tb_3S_3N	N_2	TbN
**$Tb_3N_2Cl_2$**	Tb_2Cl_3	TbN	$TbCl_3$
**$Tb_3N_2As_2$**	TbAs	As	TbN
**$Tb_3N_2Se_2$**	$Tb_4Se_3N_2$	N_2	TbN
**$Tb_3N_2Br_2$**	$TbBr_3$	Tb	TbN
**$Tb_3N_2Sb_2$**	Tb_2Sb_5	TbSb	TbN
**$Tb_3N_2Te_2$**	$Tb_4Te_3N_2$	$TbTe_3$	TbN
**$Tb_3N_2I_2$**	TbI_3	Tb	TbN
**Tb_4N_3**	TbN	Tb	
**$Tb_4N_3O_2$**	Tb_2O_3	TbN	N_2
**$Tb_4N_3F_2$**	Tb	TbN	TbF_3

**$Tb_4N_3P_2$**	TbP	P_3N_5	TbN
**$Tb_4N_3S_2$**	Tb_3S_3N	N_2	TbN
**$Tb_4N_3Cl_2$**	Tb_2Cl_3	TbN	$TbCl_3$
**$Tb_4N_3As_2$**	TbAs	As	TbN
**$Tb_4N_3Se_2$**	$Tb_4Se_3N_2$	N_2	TbN
**$Tb_4N_3Br_2$**	$TbBr_3$	Tb	TbN
**$Tb_4N_3Sb_2$**	Tb_2Sb_5	TbSb	TbN
**$Tb_4N_3Te_2$**	$Tb_4Te_3N_2$	$TbTe_3$	TbN
**$Tb_4N_3I_2$**	TbI_3	Tb	TbN
**Tb_5N_4**	TbN	Tb	
**$Tb_5N_4O_2$**	Tb_2O_3	TbN	N_2
**$Tb_5N_4F_2$**	Tb	TbN	TbF_3
**$Tb_5N_4P_2$**	TbP	P_3N_5	TbN
**$Tb_5N_4S_2$**	Tb_3S_3N	N_2	TbN
**$Tb_5N_4Cl_2$**	Tb_2Cl_3	TbN	$TbCl_3$
**$Tb_5N_4As_2$**	TbAs	As	TbN
**$Tb_5N_4Se_2$**	$Tb_4Se_3N_2$	N_2	TbN
**$Tb_5N_4Br_2$**	$TbBr_3$	Tb	TbN
**$Tb_5N_4Sb_2$**	Tb_2Sb_5	TbSb	TbN
**$Tb_5N_4Te_2$**	$Tb_4Te_3N_2$	$TbTe_3$	TbN
**$Tb_5N_4I_2$**	TbI_3	Tb	TbN
**Dy_2C**	Dy_2C		
**Dy_2CO_2**	Dy_3C_4	Dy_2O_3	C
**Dy_2CF_2**	Dy_4C_5	Dy_2C	DyF_3
**Dy_2CP_2**	DyP	C	
**Dy_2CS_2**	C	DyS	
**Dy_2CCl_2**	Dy_2C	Dy_4C_5	$DyCl_3$
**Dy_2CAs_2**	DyAs	C	
**Dy_2CSe_2**	C	DySe	
**Dy_2CBr_2**	Dy_2C	$Dy_5C_2Br_9$	Dy_4C_5
**Dy_2CSb_2**	DySb	C	
**Dy_2CTe_2**	Dy_3C_4	Dy_2Te_3	C
**Dy_2CI_2**	Dy_2C	Dy_2CI_3	
**Dy_3C_2**	Dy_2C	Dy_4C_5	
**$Dy_3C_2O_2$**	Dy_2C	Dy_2O_3	Dy_4C_5
**$Dy_3C_2F_2$**	Dy_4C_5	Dy_2C	DyF_3
**$Dy_3C_2P_2$**	Dy_3C_4	DyP	C
**$Dy_3C_2S_2$**	Dy_3C_4	C	DyS
**$Dy_3C_2Cl_2$**	Dy_2C	Dy_4C_5	$DyCl_3$
**$Dy_3C_2As_2$**	Dy_3C_4	DyAs	C
**$Dy_3C_2Se_2$**	Dy_3C_4	C	DySe

**$Dy_3C_2Br_2$**	Dy_2C	$Dy_5C_2Br_9$	Dy_4C_5
**$Dy_3C_2Sb_2$**	Dy_3C_4	DySb	C
**$Dy_3C_2Te_2$**	DyTe	Dy_3C_4	Dy_2Te_3
**$Dy_3C_2I_2$**	Dy_4C_5	Dy_2C	Dy_2CI_3
**Dy_4C_3**	Dy_2C	Dy_4C_5	
**$Dy_4C_3O_2$**	Dy_2C	Dy_2O_3	Dy_4C_5
**$Dy_4C_3F_2$**	Dy_4C_5	Dy_2C	DyF_3
**$Dy_4C_3P_2$**	Dy_3C_4	DyP	C
**$Dy_4C_3S_2$**	Dy_3C_4	C	DyS
**$Dy_4C_3Cl_2$**	Dy_2C	Dy_4C_5	$DyCl_3$
**$Dy_4C_3As_2$**	Dy_3C_4	DyAs	C
**$Dy_4C_3Se_2$**	Dy_3C_4	C	DySe
**$Dy_4C_3Br_2$**	Dy_2C	$Dy_5C_2Br_9$	Dy_4C_5
**$Dy_4C_3Sb_2$**	Dy_3C_4	DySb	C
**$Dy_4C_3Te_2$**	DyTe	Dy_3C_4	Dy_2Te_3
**$Dy_4C_3I_2$**	Dy_4C_5	Dy_2C	Dy_2CI_3
**Dy_5C_4**	Dy_2C	Dy_4C_5	
**$Dy_5C_4O_2$**	Dy_2C	Dy_2O_3	Dy_4C_5
**$Dy_5C_4F_2$**	Dy_4C_5	Dy_2C	DyF_3
**$Dy_5C_4P_2$**	Dy_3C_4	DyP	
**$Dy_5C_4S_2$**	Dy_3C_4	DyS	
**$Dy_5C_4Cl_2$**	Dy_2C	Dy_4C_5	$DyCl_3$
**$Dy_5C_4As_2$**	Dy_3C_4	DyAs	
**$Dy_5C_4Se_2$**	Dy_3C_4	DySe	
**$Dy_5C_4Br_2$**	Dy_2C	$Dy_5C_2Br_9$	Dy_4C_5
**$Dy_5C_4Sb_2$**	Dy_3C_4	DySb	
**$Dy_5C_4Te_2$**	DyTe	Dy_3C_4	
**$Dy_5C_4I_2$**	Dy_4C_5	Dy_2C	Dy_2CI_3
**Dy_2N**	DyN	Dy	
**Dy_2NO_2**	Dy_2O_3	DyN	N_2
**Dy_2NF_2**	Dy	DyN	DyF_3
**Dy_2NP_2**	DyP	P_3N_5	DyN
**Dy_2NS_2**	Dy_3S_3N	N_2	
**Dy_2NCl_2**	DyN	$DyCl_3$	Dy
**Dy_2NAs_2**	DyAs	As	DyN
**Dy_2NSe_2**	Dy_3Se_3N	N_2	
**Dy_2NBr_2**	$DyBr_3$	Dy	DyN
**Dy_2NSb_2**	Dy_2Sb_5	DySb	DyN
**Dy_2NTe_2**	$Dy_4Te_3N_2$	$DyTe_3$	DyN
**Dy_2NI_2**	DyI_3	Dy	DyN
**Dy_3N_2**	DyN	Dy	

**$Dy_3N_2O_2$**	Dy_2O_3	DyN	N_2
**$Dy_3N_2F_2$**	Dy	DyN	DyF_3
**$Dy_3N_2P_2$**	DyP	P_3N_5	DyN
**$Dy_3N_2S_2$**	Dy_3S_3N	N_2	DyN
**$Dy_3N_2Cl_2$**	DyN	$DyCl_3$	Dy
**$Dy_3N_2As_2$**	DyAs	As	DyN
**$Dy_3N_2Se_2$**	Dy_3Se_3N	N_2	DyN
**$Dy_3N_2Br_2$**	$DyBr_3$	Dy	DyN
**$Dy_3N_2Sb_2$**	Dy_2Sb_5	DySb	DyN
**$Dy_3N_2Te_2$**	$Dy_4Te_3N_2$	$DyTe_3$	DyN
**$Dy_3N_2I_2$**	DyI_3	Dy	DyN
**Dy_4N_3**	DyN	Dy	
**$Dy_4N_3O_2$**	Dy_2O_3	DyN	N_2
**$Dy_4N_3F_2$**	Dy	DyN	DyF_3
**$Dy_4N_3P_2$**	DyP	P_3N_5	DyN
**$Dy_4N_3S_2$**	Dy_3S_3N	N_2	DyN
**$Dy_4N_3Cl_2$**	DyN	$DyCl_3$	Dy
**$Dy_4N_3As_2$**	DyAs	As	DyN
**$Dy_4N_3Se_2$**	Dy_3Se_3N	N_2	DyN
**$Dy_4N_3Br_2$**	$DyBr_3$	Dy	DyN
**$Dy_4N_3Sb_2$**	Dy_2Sb_5	DySb	DyN
**$Dy_4N_3Te_2$**	$Dy_4Te_3N_2$	$DyTe_3$	DyN
**$Dy_4N_3I_2$**	DyI_3	Dy	DyN
**Dy_5N_4**	DyN	Dy	
**$Dy_5N_4O_2$**	Dy_2O_3	DyN	N_2
**$Dy_5N_4F_2$**	Dy	DyN	DyF_3
**$Dy_5N_4P_2$**	DyP	P_3N_5	DyN
**$Dy_5N_4S_2$**	Dy_3S_3N	N_2	DyN
**$Dy_5N_4Cl_2$**	DyN	$DyCl_3$	Dy
**$Dy_5N_4As_2$**	DyAs	As	DyN
**$Dy_5N_4Se_2$**	Dy_3Se_3N	N_2	DyN
**$Dy_5N_4Br_2$**	$DyBr_3$	Dy	DyN
**$Dy_5N_4Sb_2$**	Dy_2Sb_5	DySb	DyN
**$Dy_5N_4Te_2$**	$Dy_4Te_3N_2$	$DyTe_3$	DyN
**$Dy_5N_4I_2$**	DyI_3	Dy	DyN
**Ho_2C**	Ho_2C		
**Ho_2CO_2**	Ho_3C_4	Ho_4C_7	Ho_2O_3
**Ho_2CF_2**	Ho_2CF_2		
**Ho_2CP_2**	HoP	C	
**Ho_2CS_2**	C	HoS	
**Ho_2CCl_2**	Ho_2C	Ho_4C_5	$HoCl_3$

**Ho_2CAs_2**	HoAs	C	
**Ho_2CSe_2**	C	HoSe	
**Ho_2CBr_2**	Ho_2C	Ho_4C_5	$HoBr_3$
**Ho_2CSb_2**	HoSb	C	
**Ho_2CTe_2**	Ho_4C_7	Ho_3C_4	Ho_2Te_3
**Ho_2CI_2**	Ho_4C_5	Ho_2C	HoI_3
**Ho_3C_2**	Ho_2C	Ho_4C_5	
**$Ho_3C_2O_2$**	Ho_2C	Ho_2O_3	Ho_4C_5
**$Ho_3C_2F_2$**	Ho_4C_5	Ho_2C	Ho_2CF_2
**$Ho_3C_2P_2$**	Ho_4C_7	HoP	C
**$Ho_3C_2S_2$**	Ho_4C_7	C	HoS
**$Ho_3C_2Cl_2$**	Ho_2C	Ho_4C_5	$HoCl_3$
**$Ho_3C_2As_2$**	Ho_4C_7	HoAs	C
**$Ho_3C_2Se_2$**	Ho_4C_7	C	HoSe
**$Ho_3C_2Br_2$**	Ho_2C	Ho_4C_5	$HoBr_3$
**$Ho_3C_2Sb_2$**	Ho_4C_7	HoSb	C
**$Ho_3C_2Te_2$**	Ho_4C_5	HoTe	Ho_2Te_3
**$Ho_3C_2I_2$**	Ho_4C_5	Ho_2C	HoI_3
**Ho_4C_3**	Ho_2C	Ho_4C_5	
**$Ho_4C_3O_2$**	Ho_2C	Ho_2O_3	Ho_4C_5
**$Ho_4C_3F_2$**	Ho_4C_5	Ho_2C	Ho_2CF_2
**$Ho_4C_3P_2$**	Ho_4C_7	Ho_3C_4	HoP
**$Ho_4C_3S_2$**	Ho_3C_4	Ho_4C_7	HoS
**$Ho_4C_3Cl_2$**	Ho_2C	Ho_4C_5	$HoCl_3$
**$Ho_4C_3As_2$**	Ho_4C_7	Ho_3C_4	HoAs
**$Ho_4C_3Se_2$**	Ho_3C_4	Ho_4C_7	HoSe
**$Ho_4C_3Br_2$**	Ho_2C	Ho_4C_5	$HoBr_3$
**$Ho_4C_3Sb_2$**	Ho_4C_7	Ho_3C_4	HoSb
**$Ho_4C_3Te_2$**	Ho_4C_5	HoTe	Ho_2Te_3
**$Ho_4C_3I_2$**	Ho_4C_5	Ho_2C	HoI_3
**Ho_5C_4**	Ho_2C	Ho_4C_5	
**$Ho_5C_4O_2$**	Ho_2C	Ho_2O_3	Ho_4C_5
**$Ho_5C_4F_2$**	Ho_4C_5	Ho_2C	Ho_2CF_2
**$Ho_5C_4P_2$**	Ho_3C_4	HoP	
**$Ho_5C_4S_2$**	Ho_3C_4	HoS	
**$Ho_5C_4Cl_2$**	Ho_2C	Ho_4C_5	$HoCl_3$
**$Ho_5C_4As_2$**	Ho_3C_4	HoAs	
**$Ho_5C_4Se_2$**	Ho_3C_4	HoSe	
**$Ho_5C_4Br_2$**	Ho_2C	Ho_4C_5	$HoBr_3$
**$Ho_5C_4Sb_2$**	Ho_3C_4	HoSb	
**$Ho_5C_4Te_2$**	Ho_4C_5	HoTe	Ho_2Te_3

**$Ho_5C_4I_2$**	Ho_4C_5	Ho_2C	HoI_3
**Ho_2N**	HoN	Ho	
**Ho_2NO_2**	HoN	Ho_2O_3	N_2
**Ho_2NF_2**	Ho	HoN	HoF_3
**Ho_2NP_2**	HoP	P_3N_5	HoN
**Ho_2NS_2**	Ho_2S_3	N_2	HoN
**Ho_2NCl_2**	HoN	$HoCl_3$	Ho
**Ho_2NAs_2**	HoAs	As	HoN
**Ho_2NSe_2**	Ho_3Se_3N	N_2	
**Ho_2NBr_2**	$HoBr_3$	Ho	HoN
**Ho_2NSb_2**	Ho_2Sb_5	HoSb	HoN
**Ho_2NTe_2**	Ho_3Te_3N	$HoTe_3$	HoN
**Ho_2NI_2**	HoI_3	Ho	HoN
**Ho_3N_2**	HoN	Ho	
**$Ho_3N_2O_2$**	HoN	Ho_2O_3	N_2
**$Ho_3N_2F_2$**	Ho	HoN	HoF_3
**$Ho_3N_2P_2$**	HoP	P_3N_5	HoN
**$Ho_3N_2S_2$**	Ho_2S_3	N_2	HoN
**$Ho_3N_2Cl_2$**	HoN	$HoCl_3$	Ho
**$Ho_3N_2As_2$**	HoAs	As	HoN
**$Ho_3N_2Se_2$**	Ho_3Se_3N	N_2	HoN
**$Ho_3N_2Br_2$**	$HoBr_3$	Ho	HoN
**$Ho_3N_2Sb_2$**	Ho_2Sb_5	HoSb	HoN
**$Ho_3N_2Te_2$**	Ho_3Te_3N	$HoTe_3$	HoN
**$Ho_3N_2I_2$**	HoI_3	Ho	HoN
**Ho_4N_3**	HoN	Ho	
**$Ho_4N_3O_2$**	HoN	Ho_2O_3	N_2
**$Ho_4N_3F_2$**	Ho	HoN	HoF_3
**$Ho_4N_3P_2$**	HoP	P_3N_5	HoN
**$Ho_4N_3S_2$**	Ho_2S_3	N_2	HoN
**$Ho_4N_3Cl_2$**	HoN	$HoCl_3$	Ho
**$Ho_4N_3As_2$**	HoAs	As	HoN
**$Ho_4N_3Se_2$**	Ho_3Se_3N	N_2	HoN
**$Ho_4N_3Br_2$**	$HoBr_3$	Ho	HoN
**$Ho_4N_3Sb_2$**	Ho_2Sb_5	HoSb	HoN
**$Ho_4N_3Te_2$**	Ho_3Te_3N	$HoTe_3$	HoN
**$Ho_4N_3I_2$**	HoI_3	Ho	HoN
**Ho_5N_4**	HoN	Ho	
**$Ho_5N_4O_2$**	HoN	Ho_2O_3	N_2
**$Ho_5N_4F_2$**	Ho	HoN	HoF_3
**$Ho_5N_4P_2$**	HoP	P_3N_5	HoN

**$Ho_5N_4S_2$**	Ho_2S_3	N_2	HoN
**$Ho_5N_4Cl_2$**	HoN	$HoCl_3$	Ho
**$Ho_5N_4As_2$**	HoAs	As	HoN
**$Ho_5N_4Se_2$**	Ho_3Se_3N	N_2	HoN
**$Ho_5N_4Br_2$**	$HoBr_3$	Ho	HoN
**$Ho_5N_4Sb_2$**	Ho_2Sb_5	HoSb	HoN
**$Ho_5N_4Te_2$**	Ho_3Te_3N	$HoTe_3$	HoN
**$Ho_5N_4I_2$**	HoI_3	Ho	HoN
**Er_2C**	Er_2C		
**Er_2CO_2**	Er_4C_7	Er_2O_3	Er_3C_4
**Er_2CF_2**	Er_2C	Er_3C_4	ErF_3
**Er_2CP_2**	ErP	C	
**Er_2CS_2**	C	ErS	
**Er_2CCl_2**	Er_2C	Er_3C_4	$ErCl_3$
**Er_2CAs_2**	ErAs	C	
**Er_2CSe_2**	Er_4C_7	Er_3C_4	Er_2Se_3
**Er_2CBr_2**	Er_2C	Er_3C_4	$Er_5C_2Br_9$
**Er_2CSb_2**	ErSb	C	
**Er_2CTe_2**	Er_4C_7	Er_3C_4	Er_2Te_3
**Er_2CI_2**	Er_2C	Er_3C_4	ErI_3
**Er_3C_2**	Er_2C	Er_3C_4	
**$Er_3C_2O_2$**	Er_2C	Er_2O_3	Er_3C_4
**$Er_3C_2F_2$**	Er_2C	Er_3C_4	ErF_3
**$Er_3C_2P_2$**	Er_4C_7	ErP	C
**$Er_3C_2S_2$**	Er_4C_7	C	ErS
**$Er_3C_2Cl_2$**	Er_2C	Er_3C_4	$ErCl_3$
**$Er_3C_2As_2$**	Er_4C_7	ErAs	C
**$Er_3C_2Se_2$**	Er_3C_4	Er_2Se_3	ErSe
**$Er_3C_2Br_2$**	Er_2C	Er_3C_4	$Er_5C_2Br_9$
**$Er_3C_2Sb_2$**	Er_4C_7	ErSb	C
**$Er_3C_2Te_2$**	ErTe	Er_3C_4	Er_2Te_3
**$Er_3C_2I_2$**	Er_2C	Er_3C_4	ErI_3
**Er_4C_3**	Er_2C	Er_3C_4	
**$Er_4C_3O_2$**	Er_2C	Er_2O_3	Er_3C_4
**$Er_4C_3F_2$**	Er_2C	Er_3C_4	ErF_3
**$Er_4C_3P_2$**	Er_4C_7	Er_3C_4	ErP
**$Er_4C_3S_2$**	Er_4C_7	Er_3C_4	ErS
**$Er_4C_3Cl_2$**	Er_2C	Er_3C_4	$ErCl_3$
**$Er_4C_3As_2$**	Er_4C_7	Er_3C_4	ErAs
**$Er_4C_3Se_2$**	Er_3C_4	Er_2Se_3	ErSe
**$Er_4C_3Br_2$**	Er_2C	Er_3C_4	$Er_5C_2Br_9$

**$Er_4C_3Sb_2$**	Er_4C_7	Er_3C_4	ErSb
**$Er_4C_3Te_2$**	ErTe	Er_3C_4	Er_2Te_3
**$Er_4C_3I_2$**	Er_2C	Er_3C_4	ErI_3
**Er_5C_4**	Er_2C	Er_3C_4	
**$Er_5C_4O_2$**	Er_2C	Er_2O_3	Er_3C_4
**$Er_5C_4F_2$**	Er_2C	Er_3C_4	ErF_3
**$Er_5C_4P_2$**	Er_3C_4	ErP	
**$Er_5C_4S_2$**	Er_3C_4	ErS	
**$Er_5C_4Cl_2$**	Er_2C	Er_3C_4	$ErCl_3$
**$Er_5C_4As_2$**	Er_3C_4	ErAs	
**$Er_5C_4Se_2$**	Er_3C_4	ErSe	
**$Er_5C_4Br_2$**	Er_2C	Er_3C_4	$Er_5C_2Br_9$
**$Er_5C_4Sb_2$**	Er_3C_4	ErSb	
**$Er_5C_4Te_2$**	ErTe	Er_3C_4	
**$Er_5C_4I_2$**	Er_2C	Er_3C_4	ErI_3
**Er_2N**	ErN	Er	
**Er_2NO_2**	ErN	Er_2O_3	N_2
**Er_2NF_2**	Er	ErN	ErF_3
**Er_2NP_2**	ErP	P_3N_5	ErN
**Er_2NS_2**	Er_2S_3	N_2	ErN
**Er_2NCl_2**	ErN	$ErCl_3$	Er
**Er_2NAs_2**	ErAs	As	ErN
**Er_2NSe_2**	Er_2Se_3	N_2	ErN
**Er_2NBr_2**	Er	Br	ErN
**Er_2NSb_2**	ErSb	Sb	ErN
**Er_2NTe_2**	Er_2Te_3	$ErTe_3$	ErN
**Er_2NI_2**	ErI_3	Er	ErN
**Er_3N_2**	ErN	Er	
**$Er_3N_2O_2$**	ErN	Er_2O_3	N_2
**$Er_3N_2F_2$**	Er	ErN	ErF_3
**$Er_3N_2P_2$**	ErP	P_3N_5	ErN
**$Er_3N_2S_2$**	Er_2S_3	N_2	ErN
**$Er_3N_2Cl_2$**	ErN	$ErCl_3$	Er
**$Er_3N_2As_2$**	ErAs	As	ErN
**$Er_3N_2Se_2$**	Er_2Se_3	N_2	ErN
**$Er_3N_2Br_2$**	Er	Br	ErN
**$Er_3N_2Sb_2$**	ErSb	Sb	ErN
**$Er_3N_2Te_2$**	Er_2Te_3	$ErTe_3$	ErN
**$Er_3N_2I_2$**	ErI_3	Er	ErN
**Er_4N_3**	ErN	Er	
**$Er_4N_3O_2$**	ErN	Er_2O_3	N_2

**$Er_4N_3F_2$**	Er	ErN	ErF_3
**$Er_4N_3P_2$**	ErP	P_3N_5	ErN
**$Er_4N_3S_2$**	Er_2S_3	N_2	ErN
**$Er_4N_3Cl_2$**	ErN	$ErCl_3$	Er
**$Er_4N_3As_2$**	ErAs	As	ErN
**$Er_4N_3Se_2$**	Er_2Se_3	N_2	ErN
**$Er_4N_3Br_2$**	Er	Br	ErN
**$Er_4N_3Sb_2$**	ErSb	Sb	ErN
**$Er_4N_3Te_2$**	Er_2Te_3	$ErTe_3$	ErN
**$Er_4N_3I_2$**	ErI_3	Er	ErN
**Er_5N_4**	ErN	Er	
**$Er_5N_4O_2$**	ErN	Er_2O_3	N_2
**$Er_5N_4F_2$**	Er	ErN	ErF_3
**$Er_5N_4P_2$**	ErP	P_3N_5	ErN
**$Er_5N_4S_2$**	Er_2S_3	N_2	ErN
**$Er_5N_4Cl_2$**	ErN	$ErCl_3$	Er
**$Er_5N_4As_2$**	ErAs	As	ErN
**$Er_5N_4Se_2$**	Er_2Se_3	N_2	ErN
**$Er_5N_4Br_2$**	Er	Br	ErN
**$Er_5N_4Sb_2$**	ErSb	Sb	ErN
**$Er_5N_4Te_2$**	Er_2Te_3	$ErTe_3$	ErN
**$Er_5N_4I_2$**	ErI_3	Er	ErN
**Tm_2C**	Tm_2C		
**Tm_2CO_2**	Tm_4C_7	Tm_2O_3	Tm_3C_4
**Tm_2CF_2**	Tm_2C	Tm_3C_4	TmF_3
**Tm_2CP_2**	TmP	C	
**Tm_2CS_2**	C	TmS	
**Tm_2CCl_2**	Tm_2C	Tm_3C_4	$TmCl_3$
**Tm_2CAs_2**	TmAs	C	
**Tm_2CSe_2**	Tm_4C_7	Tm_3C_4	Tm_2Se_3
**Tm_2CBr_2**	Tm_2C	Tm_3C_4	$TmBr_3$
**Tm_2CSb_2**	TmSb	C	
**Tm_2CTe_2**	Tm_4C_7	Tm_3C_4	Tm_2Te_3
**Tm_2CI_2**	Tm_2C	Tm_3C_4	TmI_3
**Tm_3C_2**	Tm_2C	Tm_3C_4	
**$Tm_3C_2O_2$**	Tm_2C	Tm_2O_3	Tm_3C_4
**$Tm_3C_2F_2$**	Tm_2C	Tm_3C_4	TmF_3
**$Tm_3C_2P_2$**	Tm_4C_7	TmP	C
**$Tm_3C_2S_2$**	Tm_4C_7	C	TmS
**$Tm_3C_2Cl_2$**	Tm_2C	Tm_3C_4	$TmCl_3$
**$Tm_3C_2As_2$**	Tm_4C_7	TmAs	C

**$Tm_3C_2Se_2$**	Tm_3C_4	Tm_2Se_3	TmSe
**$Tm_3C_2Br_2$**	Tm_2C	Tm_3C_4	$TmBr_3$
**$Tm_3C_2Sb_2$**	Tm_4C_7	TmSb	C
**$Tm_3C_2Te_2$**	TmTe	Tm_3C_4	Tm_2Te_3
**$Tm_3C_2I_2$**	Tm_2C	Tm_3C_4	TmI_3
**Tm_4C_3**	Tm_2C	Tm_3C_4	
**$Tm_4C_3O_2$**	Tm_2C	Tm_2O_3	Tm_3C_4
**$Tm_4C_3F_2$**	Tm_2C	Tm_3C_4	TmF_3
**$Tm_4C_3P_2$**	Tm_4C_7	Tm_3C_4	TmP
**$Tm_4C_3S_2$**	Tm_4C_7	Tm_3C_4	TmS
**$Tm_4C_3Cl_2$**	Tm_2C	Tm_3C_4	$TmCl_3$
**$Tm_4C_3As_2$**	Tm_4C_7	Tm_3C_4	TmAs
**$Tm_4C_3Se_2$**	Tm_3C_4	Tm_2Se_3	TmSe
**$Tm_4C_3Br_2$**	Tm_2C	Tm_3C_4	$TmBr_3$
**$Tm_4C_3Sb_2$**	Tm_4C_7	Tm_3C_4	TmSb
**$Tm_4C_3Te_2$**	TmTe	Tm_3C_4	Tm_2Te_3
**$Tm_4C_3I_2$**	Tm_2C	Tm_3C_4	TmI_3
**Tm_5C_4**	Tm_2C	Tm_3C_4	
**$Tm_5C_4O_2$**	Tm_2C	Tm_2O_3	Tm_3C_4
**$Tm_5C_4F_2$**	Tm_2C	Tm_3C_4	TmF_3
**$Tm_5C_4P_2$**	Tm_3C_4	TmP	
**$Tm_5C_4S_2$**	Tm_3C_4	TmS	
**$Tm_5C_4Cl_2$**	Tm_2C	Tm_3C_4	$TmCl_3$
**$Tm_5C_4As_2$**	Tm_3C_4	TmAs	
**$Tm_5C_4Se_2$**	Tm_3C_4	TmSe	
**$Tm_5C_4Br_2$**	Tm_2C	Tm_3C_4	$TmBr_3$
**$Tm_5C_4Sb_2$**	Tm_3C_4	TmSb	
**$Tm_5C_4Te_2$**	TmTe	Tm_3C_4	
**$Tm_5C_4I_2$**	Tm_2C	Tm_3C_4	TmI_3
**Tm_2N**	TmN	Tm	
**Tm_2NO_2**	Tm_2O_3	TmN	N_2
**Tm_2NF_2**	Tm	TmN	TmF_3
**Tm_2NP_2**	TmP	P_3N_5	TmN
**Tm_2NS_2**	Tm_2S_3	N_2	TmN
**Tm_2NCl_2**	TmN	$TmCl_3$	Tm
**Tm_2NAs_2**	TmAs	As	TmN
**Tm_2NSe_2**	Tm_2Se_3	N_2	TmN
**Tm_2NBr_2**	$TmBr_3$	Tm	TmN
**Tm_2NSb_2**	TmSb	Sb	TmN
**Tm_2NTe_2**	Tm_2Te_3	$TmTe_3$	TmN
**Tm_2NI_2**	TmI_3	Tm	TmN

**Tm_3N_2**	TmN	Tm	
**$Tm_3N_2O_2$**	Tm_2O_3	TmN	N_2
**$Tm_3N_2F_2$**	Tm	TmN	TmF_3
**$Tm_3N_2P_2$**	TmP	P_3N_5	TmN
**$Tm_3N_2S_2$**	Tm_2S_3	N_2	TmN
**$Tm_3N_2Cl_2$**	TmN	$TmCl_3$	Tm
**$Tm_3N_2As_2$**	TmAs	As	TmN
**$Tm_3N_2Se_2$**	Tm_2Se_3	N_2	TmN
**$Tm_3N_2Br_2$**	$TmBr_3$	Tm	TmN
**$Tm_3N_2Sb_2$**	TmSb	Sb	TmN
**$Tm_3N_2Te_2$**	Tm_2Te_3	$TmTe_3$	TmN
**$Tm_3N_2I_2$**	TmI_3	Tm	TmN
**Tm_4N_3**	TmN	Tm	
**$Tm_4N_3O_2$**	Tm_2O_3	TmN	N_2
**$Tm_4N_3F_2$**	Tm	TmN	TmF_3
**$Tm_4N_3P_2$**	TmP	P_3N_5	TmN
**$Tm_4N_3S_2$**	Tm_2S_3	N_2	TmN
**$Tm_4N_3Cl_2$**	TmN	$TmCl_3$	Tm
**$Tm_4N_3As_2$**	TmAs	As	TmN
**$Tm_4N_3Se_2$**	Tm_2Se_3	N_2	TmN
**$Tm_4N_3Br_2$**	$TmBr_3$	Tm	TmN
**$Tm_4N_3Sb_2$**	TmSb	Sb	TmN
**$Tm_4N_3Te_2$**	Tm_2Te_3	$TmTe_3$	TmN
**$Tm_4N_3I_2$**	TmI_3	Tm	TmN
**Tm_5N_4**	TmN	Tm	
**$Tm_5N_4O_2$**	Tm_2O_3	TmN	N_2
**$Tm_5N_4F_2$**	Tm	TmN	TmF_3
**$Tm_5N_4P_2$**	TmP	P_3N_5	TmN
**$Tm_5N_4S_2$**	Tm_2S_3	N_2	TmN
**$Tm_5N_4Cl_2$**	TmN	$TmCl_3$	Tm
**$Tm_5N_4As_2$**	TmAs	As	TmN
**$Tm_5N_4Se_2$**	Tm_2Se_3	N_2	TmN
**$Tm_5N_4Br_2$**	$TmBr_3$	Tm	TmN
**$Tm_5N_4Sb_2$**	TmSb	Sb	TmN
**$Tm_5N_4Te_2$**	Tm_2Te_3	$TmTe_3$	TmN
**$Tm_5N_4I_2$**	TmI_3	Tm	TmN
**Lu_2C**	Lu_2C		
**Lu_2CO_2**	Lu_4C_7	Lu_2O_3	Lu_3C_4
**Lu_2CF_2**	Lu_2C	Lu_3C_4	LuF_3
**Lu_2CP_2**	LuP	C	
**Lu_2CS_2**	C	LuS	

**Lu_2CCl_2**	Lu_2CCl_2		
**Lu_2CAs_2**	LuAs	C	
**Lu_2CSe_2**	Lu_4C_7	Lu_3C_4	Lu_2Se_3
**Lu_2CBr_2**	Lu_2C	Lu_3C_4	$LuBr_3$
**Lu_2CSb_2**	LuSb	C	
**Lu_2CTe_2**	Lu_4C_7	Lu_3C_4	Lu_2Te_3
**Lu_2CI_2**	Lu_2C	Lu_3C_4	LuI_3
**Lu_3C_2**	Lu_2C	Lu_3C_4	
**$Lu_3C_2O_2$**	Lu_2C	Lu_2O_3	Lu_3C_4
**$Lu_3C_2F_2$**	Lu_2C	Lu_3C_4	LuF_3
**$Lu_3C_2P_2$**	Lu_4C_7	LuP	C
**$Lu_3C_2S_2$**	Lu_4C_7	C	LuS
**$Lu_3C_2Cl_2$**	Lu_2C	Lu_3C_4	Lu_2CCl_2
**$Lu_3C_2As_2$**	Lu_4C_7	LuAs	C
**$Lu_3C_2Se_2$**	Lu_2C	Lu_3C_4	Lu_2Se_3
**$Lu_3C_2Br_2$**	Lu_2C	Lu_3C_4	$LuBr_3$
**$Lu_3C_2Sb_2$**	Lu_4C_7	LuSb	C
**$Lu_3C_2Te_2$**	Lu_2C	Lu_3C_4	Lu_2Te_3
**$Lu_3C_2I_2$**	Lu_2C	Lu_3C_4	LuI_3
**Lu_4C_3**	Lu_2C	Lu_3C_4	
**$Lu_4C_3O_2$**	Lu_2C	Lu_2O_3	Lu_3C_4
**$Lu_4C_3F_2$**	Lu_2C	Lu_3C_4	LuF_3
**$Lu_4C_3P_2$**	Lu_4C_7	Lu_3C_4	LuP
**$Lu_4C_3S_2$**	Lu_4C_7	Lu_3C_4	LuS
**$Lu_4C_3Cl_2$**	Lu_2C	Lu_3C_4	Lu_2CCl_2
**$Lu_4C_3As_2$**	Lu_4C_7	Lu_3C_4	LuAs
**$Lu_4C_3Se_2$**	Lu_2C	Lu_3C_4	Lu_2Se_3
**$Lu_4C_3Br_2$**	Lu_2C	Lu_3C_4	$LuBr_3$
**$Lu_4C_3Sb_2$**	Lu_4C_7	Lu_3C_4	LuSb
**$Lu_4C_3Te_2$**	Lu_2C	Lu_3C_4	Lu_2Te_3
**$Lu_4C_3I_2$**	Lu_2C	Lu_3C_4	LuI_3
**Lu_5C_4**	Lu_2C	Lu_3C_4	
**$Lu_5C_4O_2$**	Lu_2C	Lu_2O_3	Lu_3C_4
**$Lu_5C_4F_2$**	Lu_2C	Lu_3C_4	LuF_3
**$Lu_5C_4P_2$**	Lu_3C_4	LuP	
**$Lu_5C_4S_2$**	Lu_3C_4	LuS	
**$Lu_5C_4Cl_2$**	Lu_2C	Lu_3C_4	Lu_2CCl_2
**$Lu_5C_4As_2$**	Lu_3C_4	LuAs	
**$Lu_5C_4Se_2$**	Lu_2C	Lu_3C_4	Lu_2Se_3
**$Lu_5C_4Br_2$**	Lu_2C	Lu_3C_4	$LuBr_3$
**$Lu_5C_4Sb_2$**	Lu_3C_4	LuSb	

**$Lu_5C_4Te_2$**	Lu_2C	Lu_3C_4	Lu_2Te_3
**$Lu_5C_4I_2$**	Lu_2C	Lu_3C_4	LuI_3
**Lu_2N**	LuN	Lu	
**Lu_2NO_2**	Lu_2O_3	LuN	N_2
**Lu_2NF_2**	Lu	LuN	LuF_3
**Lu_2NP_2**	LuP	P	LuN
**Lu_2NS_2**	Lu_2S_3	N_2	LuN
**Lu_2NCl_2**	Lu	LuN	$LuCl_3$
**Lu_2NAs_2**	LuAs	As	LuN
**Lu_2NSe_2**	Lu_2Se_3	N_2	LuN
**Lu_2NBr_2**	$LuBr_3$	Lu	LuN
**Lu_2NSb_2**	LuSb	Sb	LuN
**Lu_2NTe_2**	Lu_2Te_3	$LuTe_3$	LuN
**Lu_2NI_2**	LuI_3	Lu	LuN
**Lu_3N_2**	LuN	Lu	
**$Lu_3N_2O_2$**	Lu_2O_3	LuN	N_2
**$Lu_3N_2F_2$**	Lu	LuN	LuF_3
**$Lu_3N_2P_2$**	LuP	P	LuN
**$Lu_3N_2S_2$**	Lu_2S_3	N_2	LuN
**$Lu_3N_2Cl_2$**	Lu	LuN	$LuCl_3$
**$Lu_3N_2As_2$**	LuAs	As	LuN
**$Lu_3N_2Se_2$**	Lu_2Se_3	N_2	LuN
**$Lu_3N_2Br_2$**	$LuBr_3$	Lu	LuN
**$Lu_3N_2Sb_2$**	LuSb	Sb	LuN
**$Lu_3N_2Te_2$**	Lu_2Te_3	$LuTe_3$	LuN
**$Lu_3N_2I_2$**	LuI_3	Lu	LuN
**Lu_4N_3**	LuN	Lu	
**$Lu_4N_3O_2$**	Lu_2O_3	LuN	N_2
**$Lu_4N_3F_2$**	Lu	LuN	LuF_3
**$Lu_4N_3P_2$**	LuP	P	LuN
**$Lu_4N_3S_2$**	Lu_2S_3	N_2	LuN
**$Lu_4N_3Cl_2$**	Lu	LuN	$LuCl_3$
**$Lu_4N_3As_2$**	LuAs	As	LuN
**$Lu_4N_3Se_2$**	Lu_2Se_3	N_2	LuN
**$Lu_4N_3Br_2$**	$LuBr_3$	Lu	LuN
**$Lu_4N_3Sb_2$**	LuSb	Sb	LuN
**$Lu_4N_3Te_2$**	Lu_2Te_3	$LuTe_3$	LuN
**$Lu_4N_3I_2$**	LuI_3	Lu	LuN
**Lu_5N_4**	LuN	Lu	
**$Lu_5N_4O_2$**	Lu_2O_3	LuN	N_2
**$Lu_5N_4F_2$**	Lu	LuN	LuF_3

**$Lu_5N_4P_2$**	LuP	P	LuN
**$Lu_5N_4S_2$**	Lu_2S_3	N_2	LuN
**$Lu_5N_4Cl_2$**	Lu	LuN	$LuCl_3$
**$Lu_5N_4As_2$**	LuAs	As	LuN
**$Lu_5N_4Se_2$**	Lu_2Se_3	N_2	LuN
**$Lu_5N_4Br_2$**	$LuBr_3$	Lu	LuN
**$Lu_5N_4Sb_2$**	LuSb	Sb	LuN
**$Lu_5N_4Te_2$**	Lu_2Te_3	$LuTe_3$	LuN
**$Lu_5N_4I_2$**	LuI_3	Lu	LuN
**Th_2C**	Th_5C	Th_4C_3	
**Th_2CO_2**	ThC	ThO_2	
**Th_2CF_2**	Th_5C	Th_4C_3	ThF_4
**Th_2CP_2**	ThP	C	
**Th_2CS_2**	C	ThS	
**Th_2CCl_2**	Th_5C	Th_4C_3	$ThCl_4$
**Th_2CAs_2**	ThAs	C	
**Th_2CSe_2**	C	ThSe	
**Th_2CBr_2**	Th_4C_3	Th_6CBr_{14}	
**Th_2CSb_2**	ThC_2	Th_3Sb_4	
**Th_2CTe_2**	ThTe	ThC_2	Th_7Te_{12}
**Th_2CI_2**	ThI_3	Th_4C_3	
**Th_3C_2**	Th_5C	Th_4C_3	
**$Th_3C_2O_2$**	ThC	ThO_2	
**$Th_3C_2F_2$**	Th_4C_3	ThC	ThF_4
**$Th_3C_2P_2$**	ThC_2	ThP	
**$Th_3C_2S_2$**	ThC_2	ThS	
**$Th_3C_2Cl_2$**	Th_4C_3	ThC	$ThCl_4$
**$Th_3C_2As_2$**	ThC_2	ThAs	
**$Th_3C_2Se_2$**	ThC_2	ThSe	
**$Th_3C_2Br_2$**	Th_4C_3	Th_6CBr_{14}	ThC
**$Th_3C_2Sb_2$**	ThSb	ThC_2	
**$Th_3C_2Te_2$**	ThTe	ThC_2	
**$Th_3C_2I_2$**	ThI_3	Th_4C_3	ThC
**Th_4C_3**	Th_4C_3		
**$Th_4C_3O_2$**	ThC	ThO_2	
**$Th_4C_3F_2$**	Th_4C_3	ThC	ThF_4
**$Th_4C_3P_2$**	Th_2C_3	ThP	
**$Th_4C_3S_2$**	Th_2C_3	ThS	
**$Th_4C_3Cl_2$**	Th_4C_3	ThC	$ThCl_4$
**$Th_4C_3As_2$**	Th_2C_3	ThAs	
**$Th_4C_3Se_2$**	Th_2C_3	ThSe	

**$Th_4C_3Br_2$**	Th_4C_3	Th_6CBr_{14}	ThC
**$Th_4C_3Sb_2$**	Th_2C_3	ThSb	
**$Th_4C_3Te_2$**	Th_2C_3	ThTe	
**$Th_4C_3I_2$**	ThI_3	Th_4C_3	ThC
**Th_5C_4**	Th_4C_3	ThC	
**$Th_5C_4O_2$**	ThC	ThO_2	
**$Th_5C_4F_2$**	Th_4C_3	ThC	ThF_4
**$Th_5C_4P_2$**	Th_2C_3	ThP	ThC
**$Th_5C_4S_2$**	Th_2C_3	ThC	ThS
**$Th_5C_4Cl_2$**	Th_4C_3	ThC	$ThCl_4$
**$Th_5C_4As_2$**	Th_2C_3	ThAs	ThC
**$Th_5C_4Se_2$**	Th_2C_3	ThC	ThSe
**$Th_5C_4Br_2$**	Th_4C_3	Th_6CBr_{14}	ThC
**$Th_5C_4Sb_2$**	Th_2C_3	ThSb	ThC
**$Th_5C_4Te_2$**	Th_2C_3	ThTe	ThC
**$Th_5C_4I_2$**	ThI_3	Th_4C_3	ThC
**Th_2N**	ThN	Th	
**Th_2NO_2**	ThN	ThO_2	
**Th_2NF_2**	ThNF	Th	ThF_4
**Th_2NP_2**	Th_3N_4	Th_3P_4	P_3N_5
**Th_2NS_2**	Th_2S_3	Th_2SN_2	
**Th_2NCl_2**	ThNCl	$ThCl_4$	Th
**Th_2NAs_2**	$ThAs_2$	Th_3As_4	Th_3N_4
**Th_2NSe_2**	Th_2Se_3	Th_2SeN_2	
**Th_2NBr_2**	ThBrN	$ThBr_4$	Th
**Th_2NSb_2**	$ThSb_2$	Th_2SbN_2	Th_3Sb_4
**Th_2NTe_2**	Th_7Te_{12}	ThTe	Th_2TeN_2
**Th_2NI_2**	ThI_3	ThIN	Th
**Th_3N_2**	ThN	Th	
**$Th_3N_2O_2$**	ThN	ThO_2	
**$Th_3N_2F_2$**	ThNF	Th	
**$Th_3N_2P_2$**	Th_3N_4	Th_3P_4	
**$Th_3N_2S_2$**	ThS	Th_2SN_2	
**$Th_3N_2Cl_2$**	ThNCl	Th	
**$Th_3N_2As_2$**	Th_3As_4	Th_3N_4	
**$Th_3N_2Se_2$**	ThSe	Th_2SeN_2	
**$Th_3N_2Br_2$**	ThBrN	Th	
**$Th_3N_2Sb_2$**	ThSb	Th_2SbN_2	
**$Th_3N_2Te_2$**	ThTe	Th_2TeN_2	
**$Th_3N_2I_2$**	ThIN	Th	
**Th_4N_3**	ThN	Th	

**$Th_4N_3O_2$**	ThN	ThO_2	
**$Th_4N_3F_2$**	ThNF	Th	ThN
**$Th_4N_3P_2$**	Th_3N_4	Th_3P_4	ThP
**$Th_4N_3S_2$**	ThS	Th_2SN_2	Th
**$Th_4N_3Cl_2$**	ThN	ThNCl	Th
**$Th_4N_3As_2$**	Th_3As_4	ThAs	Th_3N_4
**$Th_4N_3Se_2$**	ThSe	Th_2SeN_2	Th
**$Th_4N_3Br_2$**	ThBrN	Th	ThN
**$Th_4N_3Sb_2$**	ThSb	Th_2SbN_2	ThN
**$Th_4N_3Te_2$**	ThTe	Th_2TeN_2	Th
**$Th_4N_3I_2$**	ThIN	Th	ThN
**Th_5N_4**	ThN	Th	
**$Th_5N_4O_2$**	ThN	ThO_2	
**$Th_5N_4F_2$**	ThNF	Th	ThN
**$Th_5N_4P_2$**	Th_3N_4	ThP	
**$Th_5N_4S_2$**	Th_2SN_2	Th	
**$Th_5N_4Cl_2$**	ThN	ThNCl	Th
**$Th_5N_4As_2$**	ThAs	Th_3N_4	
**$Th_5N_4Se_2$**	Th_2SeN_2	Th	
**$Th_5N_4Br_2$**	ThBrN	Th	ThN
**$Th_5N_4Sb_2$**	ThSb	Th_2SbN_2	ThN
**$Th_5N_4Te_2$**	Th_2TeN_2	Th	
**$Th_5N_4I_2$**	ThIN	Th	ThN
**Pa_2C**	PaC	Pa	
**Pa_2CO_2**	PaC	PaO_2	
**Pa_2CF_2**	CF_4	Pa	PaC
**Pa_2CP_2**	Pa_3P_4	C	PaC
**Pa_2CS_2**	Pa_2S_3	PaC	C
**Pa_2CCl_2**	Pa	PaC	$PaCl_4$
**Pa_2CAs_2**	PaAs	C	
**Pa_2CSe_2**	Pa	PaC	$PaSe_3$
**Pa_2CBr_2**	Pa	PaC	$PaBr_4$
**Pa_2CSb_2**	$PaSb_2$	PaC	
**Pa_2CTe_2**	Pa_3Te	$PaTe_3$	PaC
**Pa_2CI_2**	Pa	PaC	PaI_3
**Pa_3C_2**	PaC	Pa	
**$Pa_3C_2O_2$**	PaC	PaO_2	
**$Pa_3C_2F_2$**	CF_4	Pa	PaC
**$Pa_3C_2P_2$**	Pa_3P_4	C	PaC
**$Pa_3C_2S_2$**	Pa_2S_3	PaC	C
**$Pa_3C_2Cl_2$**	Pa	PaC	$PaCl_4$

**$Pa_3C_2As_2$**	PaAs	C	PaC
**$Pa_3C_2Se_2$**	Pa	PaC	$PaSe_3$
**$Pa_3C_2Br_2$**	Pa	PaC	$PaBr_4$
**$Pa_3C_2Sb_2$**	$PaSb_2$	PaC	
**$Pa_3C_2Te_2$**	Pa_3Te	$PaTe_3$	PaC
**$Pa_3C_2I_2$**	Pa	PaC	PaI_3
**Pa_4C_3**	PaC	Pa	
**$Pa_4C_3O_2$**	PaC	PaO_2	
**$Pa_4C_3F_2$**	CF_4	Pa	PaC
**$Pa_4C_3P_2$**	Pa_3P_4	C	PaC
**$Pa_4C_3S_2$**	Pa_2S_3	PaC	C
**$Pa_4C_3Cl_2$**	Pa	PaC	$PaCl_4$
**$Pa_4C_3As_2$**	PaAs	C	PaC
**$Pa_4C_3Se_2$**	Pa	PaC	$PaSe_3$
**$Pa_4C_3Br_2$**	Pa	PaC	$PaBr_4$
**$Pa_4C_3Sb_2$**	$PaSb_2$	PaC	
**$Pa_4C_3Te_2$**	Pa_3Te	$PaTe_3$	PaC
**$Pa_4C_3I_2$**	Pa	PaC	PaI_3
**Pa_5C_4**	PaC	Pa	
**$Pa_5C_4O_2$**	PaC	PaO_2	
**$Pa_5C_4F_2$**	CF_4	Pa	PaC
**$Pa_5C_4P_2$**	Pa_3P_4	C	PaC
**$Pa_5C_4S_2$**	Pa_2S_3	PaC	C
**$Pa_5C_4Cl_2$**	Pa	PaC	$PaCl_4$
**$Pa_5C_4As_2$**	PaAs	C	PaC
**$Pa_5C_4Se_2$**	Pa	PaC	$PaSe_3$
**$Pa_5C_4Br_2$**	Pa	PaC	$PaBr_4$
**$Pa_5C_4Sb_2$**	$PaSb_2$	PaC	
**$Pa_5C_4Te_2$**	Pa_3Te	$PaTe_3$	PaC
**$Pa_5C_4I_2$**	Pa	PaC	PaI_3
**Pa_2N**	PaN	Pa	
**Pa_2NO_2**	PaN	PaO_2	
**Pa_2NF_2**	Pa	PaN	F_2
**Pa_2NP_2**	PaP_2	PaN	
**Pa_2NS_2**	Pa_2S_3	PaS_3	PaN
**Pa_2NCl_2**	Pa	PaN	$PaCl_4$
**Pa_2NAs_2**	$PaAs_2$	PaN	
**Pa_2NSe_2**	$PaSe_3$	Pa	PaN
**Pa_2NBr_2**	$PaBr_4$	Pa	PaN
**Pa_2NSb_2**	$PaSb_2$	PaN	
**Pa_2NTe_2**	Pa_3Te	$PaTe_3$	PaN

**Pa_2NI_2**	PaI_3	Pa	PaN
**Pa_3N_2**	PaN	Pa	
**$Pa_3N_2O_2$**	PaN	PaO_2	
**$Pa_3N_2F_2$**	Pa	PaN	F_2
**$Pa_3N_2P_2$**	PaP_2	PaN	
**$Pa_3N_2S_2$**	Pa_2S_3	PaS_3	PaN
**$Pa_3N_2Cl_2$**	Pa	PaN	$PaCl_4$
**$Pa_3N_2As_2$**	$PaAs_2$	PaN	
**$Pa_3N_2Se_2$**	$PaSe_3$	Pa	PaN
**$Pa_3N_2Br_2$**	$PaBr_4$	Pa	PaN
**$Pa_3N_2Sb_2$**	$PaSb_2$	PaN	
**$Pa_3N_2Te_2$**	Pa_3Te	$PaTe_3$	PaN
**$Pa_3N_2I_2$**	PaI_3	Pa	PaN
**Pa_4N_3**	PaN	Pa	
**$Pa_4N_3O_2$**	PaN	PaO_2	
**$Pa_4N_3F_2$**	Pa	PaN	F_2
**$Pa_4N_3P_2$**	PaP_2	PaN	
**$Pa_4N_3S_2$**	Pa_2S_3	PaS_3	PaN
**$Pa_4N_3Cl_2$**	Pa	PaN	$PaCl_4$
**$Pa_4N_3As_2$**	$PaAs_2$	PaN	
**$Pa_4N_3Se_2$**	$PaSe_3$	Pa	PaN
**$Pa_4N_3Br_2$**	$PaBr_4$	Pa	PaN
**$Pa_4N_3Sb_2$**	$PaSb_2$	PaN	
**$Pa_4N_3Te_2$**	Pa_3Te	$PaTe_3$	PaN
**$Pa_4N_3I_2$**	PaI_3	Pa	PaN
**Pa_5N_4**	PaN	Pa	
**$Pa_5N_4O_2$**	PaN	PaO_2	
**$Pa_5N_4F_2$**	Pa	PaN	F_2
**$Pa_5N_4P_2$**	PaP_2	PaN	
**$Pa_5N_4S_2$**	Pa_2S_3	PaS_3	PaN
**$Pa_5N_4Cl_2$**	Pa	PaN	$PaCl_4$
**$Pa_5N_4As_2$**	$PaAs_2$	PaN	
**$Pa_5N_4Se_2$**	$PaSe_3$	Pa	PaN
**$Pa_5N_4Br_2$**	$PaBr_4$	Pa	PaN
**$Pa_5N_4Sb_2$**	$PaSb_2$	PaN	
**$Pa_5N_4Te_2$**	Pa_3Te	$PaTe_3$	PaN
**$Pa_5N_4I_2$**	PaI_3	Pa	PaN
**U_2C**	UC	U	
**U_2CO_2**	$U_{17}O_{31}$	U_2C_3	UC
**U_2CF_2**	UC	UF_3	U
**U_2CP_2**	UP	C	

**U_2CS_2**	C	US	
**U_2CCl_2**	UC	UCl_3	U
**U_2CAs_2**	UAs	C	
**U_2CSe_2**	U_2C_3	U_2Se_3	
**U_2CBr_2**	UBr_3	UC	U
**U_2CSb_2**	U_3Sb_4	UC_2	
**U_2CTe_2**	U_2C_3	U_2Te_3	
**U_2CI_2**	UC	U	UI_3
**U_3C_2**	UC	U	
**$U_3C_2O_2$**	$U_{17}O_{31}$	U_2C_3	UC
**$U_3C_2F_2$**	UC	UF_3	U
**$U_3C_2P_2$**	UP	UC_2	
**$U_3C_2S_2$**	UC_2	US	
**$U_3C_2Cl_2$**	UC	UCl_3	U
**$U_3C_2As_2$**	UAs	UC_2	
**$U_3C_2Se_2$**	U_2C_3	U_2Se_3	UC
**$U_3C_2Br_2$**	UBr_3	UC	U
**$U_3C_2Sb_2$**	U_2C_3	U_3Sb_4	UC
**$U_3C_2Te_2$**	U_2C_3	U_2Te_3	UC
**$U_3C_2I_2$**	UC	U	UI_3
**U_4C_3**	UC	U	
**$U_4C_3O_2$**	$U_{17}O_{31}$	U_2C_3	UC
**$U_4C_3F_2$**	UC	UF_3	U
**$U_4C_3P_2$**	U_2C_3	UP	
**$U_4C_3S_2$**	U_2C_3	US	
**$U_4C_3Cl_2$**	UC	UCl_3	U
**$U_4C_3As_2$**	U_2C_3	UAs	
**$U_4C_3Se_2$**	U_2C_3	U_2Se_3	UC
**$U_4C_3Br_2$**	UBr_3	UC	U
**$U_4C_3Sb_2$**	U_2C_3	U_3Sb_4	UC
**$U_4C_3Te_2$**	U_2C_3	U_2Te_3	UC
**$U_4C_3I_2$**	UC	U	UI_3
**U_5C_4**	UC	U	
**$U_5C_4O_2$**	$U_{17}O_{31}$	U_2C_3	UC
**$U_5C_4F_2$**	UC	UF_3	U
**$U_5C_4P_2$**	U_2C_3	UP	UC
**$U_5C_4S_2$**	U_2C_3	U_2CS	US
**$U_5C_4Cl_2$**	UC	UCl_3	U
**$U_5C_4As_2$**	U_2C_3	UAs	UC
**$U_5C_4Se_2$**	U_2C_3	U_2Se_3	UC
**$U_5C_4Br_2$**	UBr_3	UC	U

**$U_5C_4Sb_2$**	U_2C_3	U_3Sb_4	UC
**$U_5C_4Te_2$**	U_2C_3	U_2Te_3	UC
**$U_5C_4I_2$**	UC	U	UI_3
**U_2N**	UN	U	
**U_2NO_2**	UO_2	UN	
**U_2NF_2**	UN	UF_3	U
**U_2NP_2**	U_3P_4	UP_2	U_2PN_2
**U_2NS_2**	U_2S_3	U_2SN_2	
**U_2NCl_2**	UN	UCl_3	U
**U_2NAs_2**	UAs_2	U_2AsN_2	U_3As_4
**U_2NSe_2**	U_2Se_3	U_2SeN_2	
**U_2NBr_2**	UBrN	UBr_3	U
**U_2NSb_2**	U_3Sb_4	U_2SbN_2	USb_2
**U_2NTe_2**	U_2TeN_2	U_2Te_3	
**U_2NI_2**	UIN	UI_3	U
**U_3N_2**	UN	U	
**$U_3N_2O_2$**	UO_2	UN	
**$U_3N_2F_2$**	UN	UF_3	U
**$U_3N_2P_2$**	U_2PN_2	UP	
**$U_3N_2S_2$**	U_2SN_2	US	
**$U_3N_2Cl_2$**	UN	UCl_3	U
**$U_3N_2As_2$**	U_2AsN_2	UAs	
**$U_3N_2Se_2$**	U_2SeN_2	USe	
**$U_3N_2Br_2$**	UBrN	U	
**$U_3N_2Sb_2$**	U_3Sb_4	U_2SbN_2	U
**$U_3N_2Te_2$**	U_2TeN_2	U_2Te_3	U
**$U_3N_2I_2$**	UIN	U	
**U_4N_3**	UN	U	
**$U_4N_3O_2$**	UO_2	UN	
**$U_4N_3F_2$**	UN	UF_3	U
**$U_4N_3P_2$**	U_2PN_2	UP	UN
**$U_4N_3S_2$**	U_2SN_2	US	UN
**$U_4N_3Cl_2$**	UN	UCl_3	U
**$U_4N_3As_2$**	U_2AsN_2	UAs	UN
**$U_4N_3Se_2$**	U_2SeN_2	USe	U
**$U_4N_3Br_2$**	UBrN	U	UN
**$U_4N_3Sb_2$**	U_3Sb_4	U_2SbN_2	U
**$U_4N_3Te_2$**	U_2TeN_2	U_2Te_3	U
**$U_4N_3I_2$**	UIN	U	UN
**U_5N_4**	UN	U	
**$U_5N_4O_2$**	UO_2	UN	

**$U_5N_4F_2$**	UN	UF_3	U
**$U_5N_4P_2$**	U_2PN_2	UP	UN
**$U_5N_4S_2$**	U_2SN_2	US	UN
**$U_5N_4Cl_2$**	UN	UCl_3	U
**$U_5N_4As_2$**	U_2AsN_2	UAs	UN
**$U_5N_4Se_2$**	U_2SeN_2	U	
**$U_5N_4Br_2$**	UBrN	U	UN
**$U_5N_4Sb_2$**	U_2SbN_2	U	
**$U_5N_4Te_2$**	U_2TeN_2	U	
**$U_5N_4I_2$**	UIN	U	UN

Table S2: The number of valence electrons of the pseudopotentials for different elements.

Element	Number of valence electrons
C	4
N	5
O	6
F	7
P	5
S	6
Cl	7
Sc	11
Ti	12
V	13
Cr	12

Element	Number of valence electrons
Mn	13
As	5
Se	6
Br	7
Y	11
Zr	12
Nb	13
Mo	14
Sb	5
Te	6
I	7

Element	Number of valence electrons
La	11
Ce	12
Pr	11
Nd	11
Sm	11
Eu	8
Gd	9
Tb	9
Dy	9
Ho	9
Er	9

Element	Number of valence electrons
Tm	9
Lu	9
Hf	10
Ta	11
W	14
Re	7
Th	12
Pa	13
U	14

Table S3: Experimentally known single transition metal MXene compositions synthesized in aqueous media

Composition	Synthesis method	Reference
Ti_2CT_x	Direct HF etching	2012, M. Naguib et al.[3]
V_2CT_x	Direct HF etching	2013, M. Naguib et al.[4]
Nb_2CT_x	Direct HF etching	2013, M. Naguib et al.[4]
Mo_2CT_x	Direct HF etching	2015, R. Meshkian et al.[5]
Mo_2NT_x	Ammoniation of carbide MXene, carbide obtained through direct HF etching	2017, P. Urbankowski et al.[6]
V_2NT_x	Ammoniation of carbide MXene, carbide obtained through direct HF etching	2017, P. Urbankowski et al.[6]
Ti_2NT_x	*In situ* HF etching	2017, B. Soundiraraju et al.[7]
$Ti_3C_2T_x$	Direct HF etching	2011, M. Naguib et al.[8]
$Zr_3C_2T_x$	Direct HF etching	2016, J. Zhou et al.[9]
$Hf_3C_2T_x$	Direct HF etching	2017, J. Zhou et al.[10]
$Ti_4N_3T_x$	Molten salt etching, washing in water	2016, P. Urabnkowski et al.[11]
$Nb_4C_3T_x$	Direct HF etching	2014, M. Ghidiu et al.[12]
$Ta_4C_3T_x$	Direct HF etching	2012, M. Naguib et al.[3]
$V_4C_3T_x$	Direct HF etching	2018, M. H. Tran et al.[13]

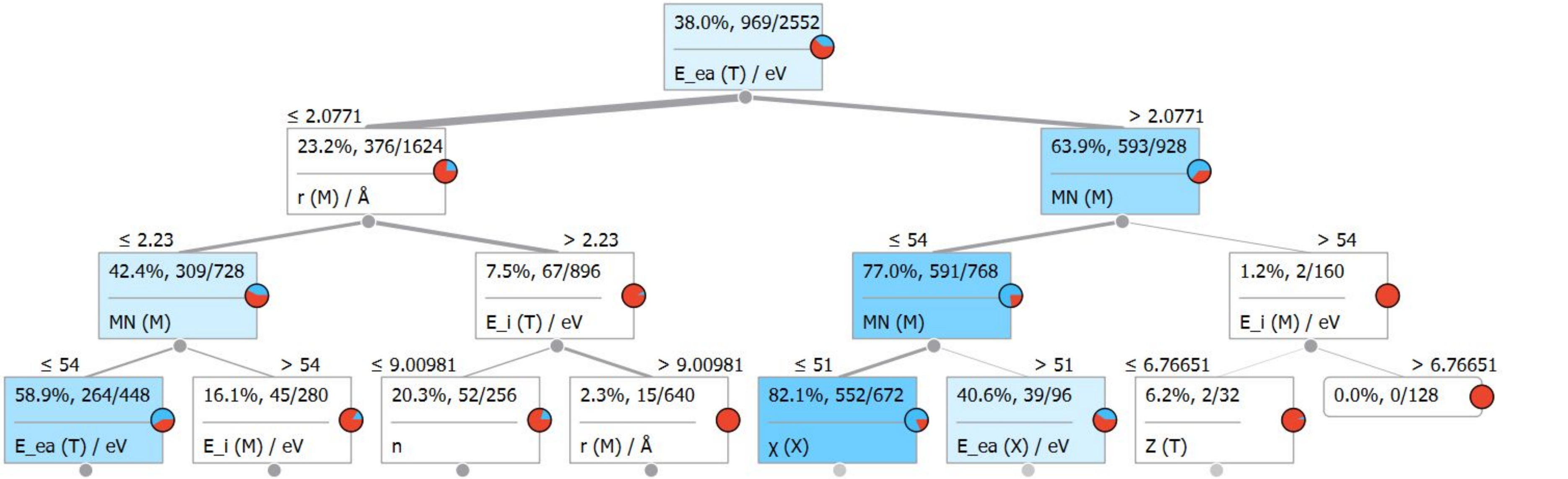

Figure S1: The first four node levels of the decision tree for classification of MXenes as thermodynamically (meta)stable or unstable. Target class is set as (meta)stable composition, i.e., ratios and percentages indicate the proportion of (meta)stable compositions. In pie charts, (meta)stable and unstable compositions are shown as blue and red, respectively. E_ea denotes electronic affinity in eV, r van der Waals radius in Å, MN Mendeleev number, Z the atomic number, and E_i the first ionization energy in eV. (T), (X), and (M) indicate that the characteristics corresponds to surface termination atom, carbon/nitrogen, and transition metal atom, respectively.

Table S4: Key performance metrics of the decision tree in Figure S1 based on the stratified 10-fold cross-validation. S and U in the confusion matrix indicate (meta)stable and unstable compositions, respectively.

Metric	Value				
F_1 score	0.917				
Precision	0.918				
Recall	0.917				
Confusion matrix			Predicted		
			S	U	Σ
	Actual	S	869	100	969
		U	111	1472	1583
		Σ	980	1572	2552

Table S5: List of parameters used for building a decision tree with interacting parameters (Figure S2)

Parameter groups	Parameters/interactions used in the model
n	*n*
van der Waals radius (r)	r(M) / r(X)
	r(M) / r(T)
	r(X) / r(T)
Pauling electronegativity (χ)	χ(M) – χ(X)
	χ(M) – χ(T)
	χ(X) – χ(T)
First ionization energy (E_i)	E_i(M) · E_i(X)
	E_i(M) · E_i(T)
	E_i(X) · E_i(T)
Electron affinity (E_{ea})	E_{ea}(M) · E_{ea}(X)
	E_{ea}(M) · E_{ea}(T)
	E_{ea}(X) · E_{ea}(T)
Mendeley number (MN)	MN(M) – MN(X)
	MN(M) – MN(T)
	MN(X) – MN(T)
First ionization energy (E_i) & electron affinity (E_{ea})	E_i(M) – E_{ea}(X)
	E_i(M) – E_{ea}(T)

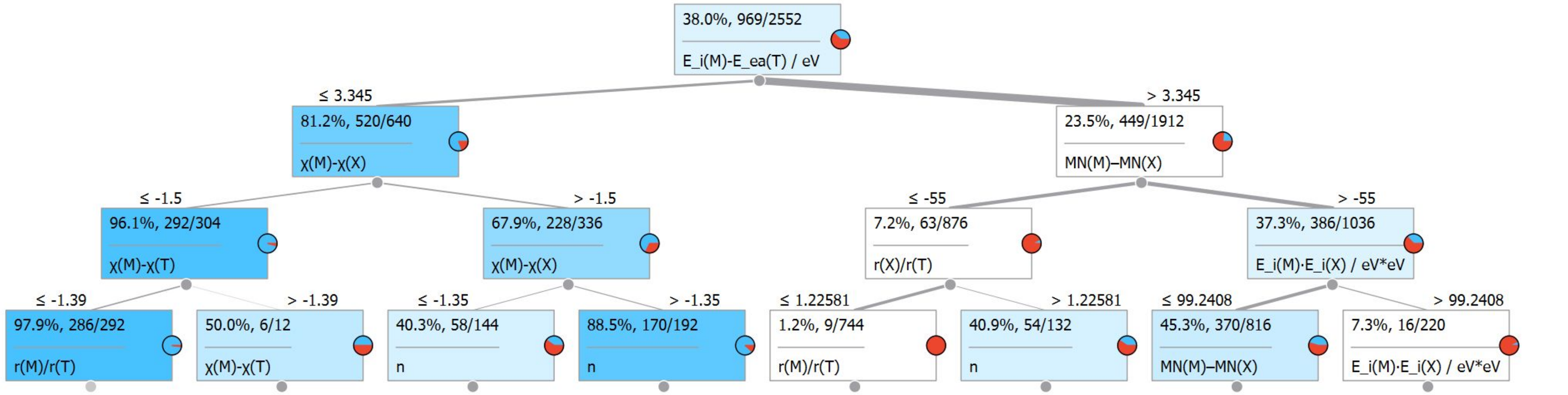

Figure S2: The first four node levels of the decision tree for classification of MXenes as thermodynamically (meta)stable or unstable based on the interacting parameters from Table S5. Target class is set as (meta)stable composition, i.e., ratios and percentages indicate the proportion of (meta)stable compositions. In pie charts, (meta)stable and unstable compositions are shown as blue and red, respectively. E_ea denotes electronic affinity in eV, r van der Waals radius in Å, MN Mendeleev number, χ the electronegativity, and E_i the first ionization energy in eV. (T), (X), and (M) indicate that the characteristics corresponds to surface termination atom, carbon/nitrogen, and transition metal atom, respectively.

Table S6: Key performance metrics of the decision tree in Figure S2 based on the stratified 10-fold cross-validation. S and U in the confusion matrix indicate (meta)stable and unstable compositions, respectively.

<table>
<tr><th>Metric</th><th>Value</th></tr>
<tr><td>F_1 score</td><td>0.926</td></tr>
<tr><td>Precision</td><td>0.926</td></tr>
<tr><td>Recall</td><td>0.926</td></tr>
<tr><td>Confusion matrix</td><td>
<table>
<tr><td></td><td></td><td colspan="3">Predicted</td></tr>
<tr><td></td><td></td><td>S</td><td>U</td><td>Σ</td></tr>
<tr><td rowspan="3">Actual</td><td>S</td><td>873</td><td>96</td><td>969</td></tr>
<tr><td>U</td><td>92</td><td>1491</td><td>1583</td></tr>
<tr><td>Σ</td><td>965</td><td>1587</td><td>2552</td></tr>
</table>
</td></tr>
</table>

Table S7: **Permutation Feature Importance analysis of the decision tree model in Figure 6a and Figure S1.** The five features with the highest decrease in area under the receiver operating characteristic curve (AUC) are listed along with standard deviation scores for each feature.

Feature	Mean AUC	Standard deviation
E_{ea}(T)	0.3158	0.007
MN(M)	0.1906	0.006
r(M)	0.1846	0.008
N	0.0886	0.005
χ(T)	0.0860	0.002

Table S8: **Permutation Feature Importance analysis of the decision tree model in Figure S2.** The five features with the highest decrease in area under the receiver operating characteristic curve (AUC) are listed along with standard deviation scores for each feature.

Feature	Mean AUC	Standard deviation
E_i(M) – E_{ea}(T)	0.2509	0.005
MN(M) – MN(X)	0.2018	0.008
χ(M) – χ(X)	0.1191	0.008
E_{ea}(X) · E_{ea}(T)	0.0923	0.004
n	0.0907	0.005

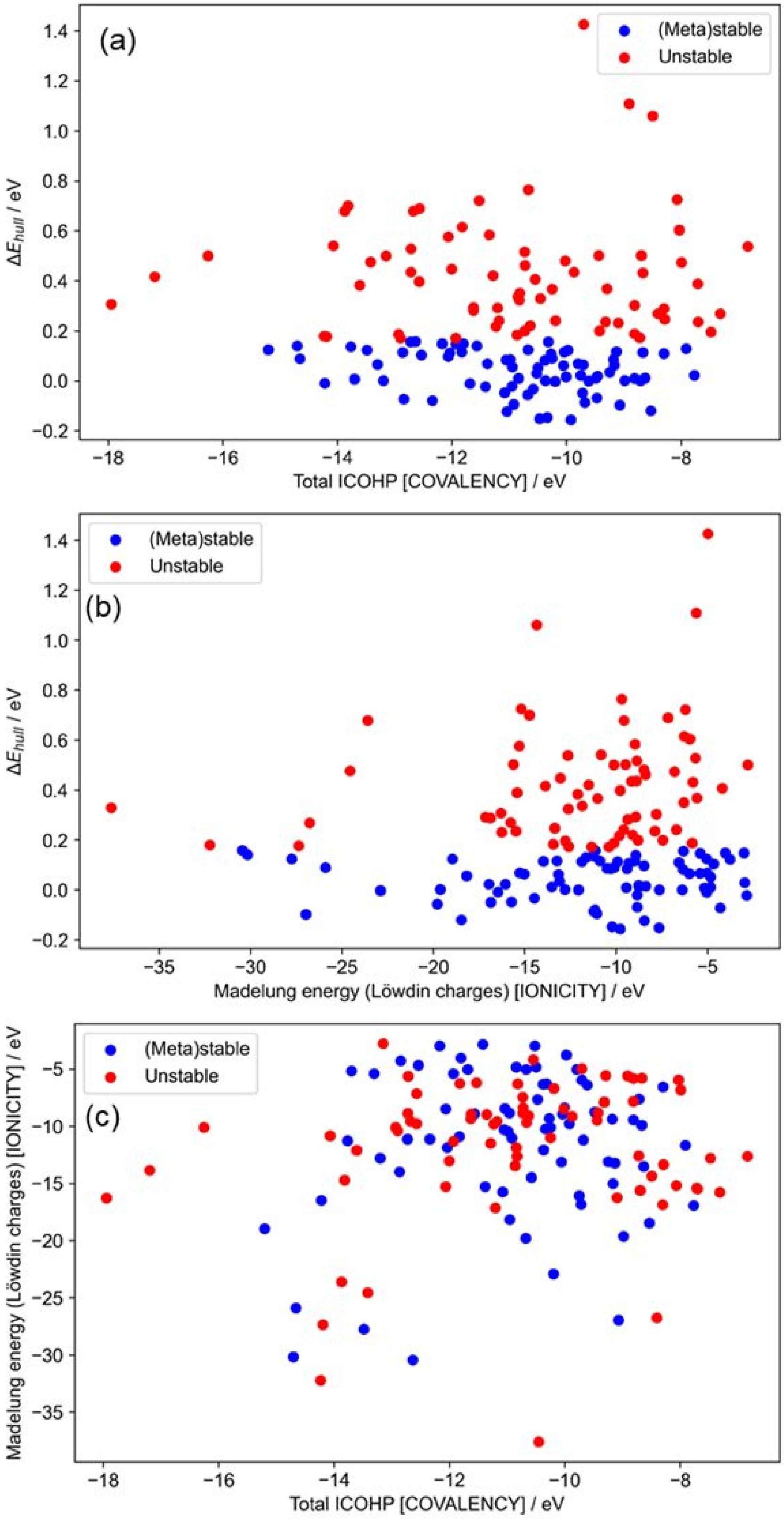

Figure S3: Analysis of the impact of the nature of chemical bonding on the thermodynamic stability for M_2CT_2 MXenes, where M is a d block transition metal: energy above the hull as a function of (a) total Integrated Crystal Orbital Hamilton Population (ICOHP – covalency) and (b) Madelung energy based on Löwdin partial charges (ionicity). (c) shows the Madelung energy as a function of total ICOHP. (Meta)stable and unstable compositions are marked in blue and red, respectively.

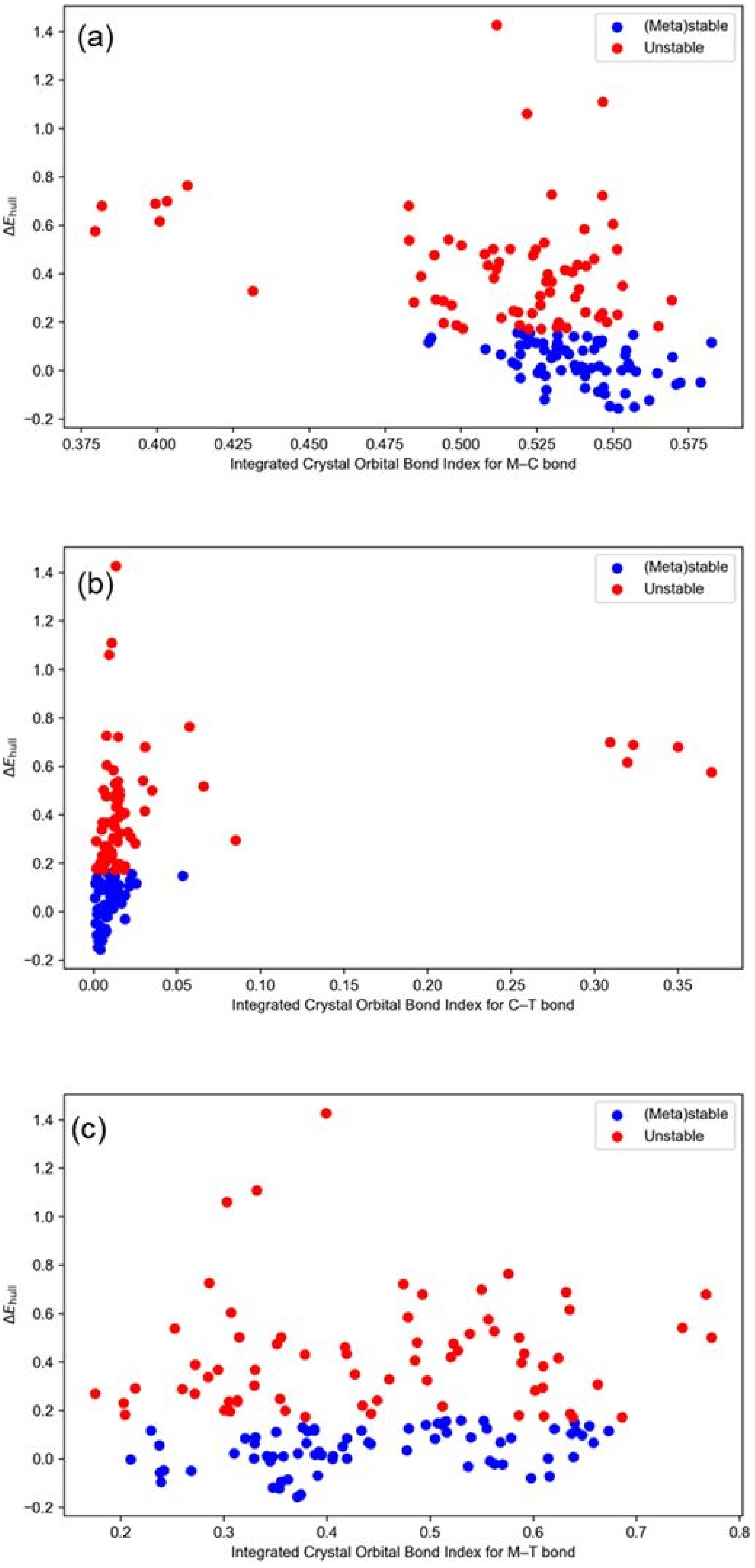

Figure S4: Energy above the hull as a function of Integrated Crystal Orbital Bond Index (ICOBI) for M_2CT_2 MXenes, where M is a d block transition metal: (a) M–C bond, (b) C–T bond, (c) M–T bond. (Meta)stable and unstable compositions are marked in blue and red, respectively.

References

(1) *Entry sets use wrong Yb pseudo-potential · Issue #2968 · materialsproject/pymatgen*. GitHub. https://github.com/materialsproject/pymatgen/issues/2968 (accessed 2024-02-09).

(2) *Database Versions*. https://docs.materialsproject.org/changes/database-versions (accessed 2024-02-09).

(3) Naguib, M.; Mashtalir, O.; Carle, J.; Presser, V.; Lu, J.; Hultman, L.; Gogotsi, Y.; Barsoum, M. W. Two-Dimensional Transition Metal Carbides. *ACS Nano* **2012**, *6* (2), 1322–1331. https://doi.org/10.1021/nn204153h.

(4) Naguib, M.; Halim, J.; Lu, J.; Cook, K. M.; Hultman, L.; Gogotsi, Y.; Barsoum, M. W. New Two-Dimensional Niobium and Vanadium Carbides as Promising Materials for Li-Ion Batteries. *J. Am. Chem. Soc.* **2013**, *135* (43), 15966–15969. https://doi.org/10.1021/ja405735d.

(5) Meshkian, R.; Näslund, L.-Å.; Halim, J.; Lu, J.; Barsoum, M. W.; Rosen, J. Synthesis of Two-Dimensional Molybdenum Carbide, Mo_2C, from the Gallium Based Atomic Laminate Mo_2Ga_2C. *Scr. Mater.* **2015**, *108*, 147–150. https://doi.org/10.1016/j.scriptamat.2015.07.003.

(6) Urbankowski, P.; Anasori, B.; Hantanasirisakul, K.; Yang, L.; Zhang, L.; Haines, B.; May, S. J.; Billinge, S. J. L.; Gogotsi, Y. 2D Molybdenum and Vanadium Nitrides Synthesized by Ammoniation of 2D Transition Metal Carbides (MXenes). *Nanoscale* **2017**, *9* (45), 17722–17730. https://doi.org/10.1039/C7NR06721F.

(7) Soundiraraju, B.; George, B. K. Two-Dimensional Titanium Nitride (Ti_2N) MXene: Synthesis, Characterization, and Potential Application as Surface-Enhanced Raman Scattering Substrate. *ACS Nano* **2017**, *11* (9), 8892–8900. https://doi.org/10.1021/acsnano.7b03129.

(8) Naguib, M.; Kurtoglu, M.; Presser, V.; Lu, J.; Niu, J.; Heon, M.; Hultman, L.; Gogotsi, Y.; Barsoum, M. W. Two-Dimensional Nanocrystals Produced by Exfoliation of Ti_3AlC_2. *Adv. Mater.* **2011**, *23* (37), 4248–4253. https://doi.org/10.1002/adma.201102306.

(9) Zhou, J.; Zha, X.; Chen, F. Y.; Ye, Q.; Eklund, P.; Du, S.; Huang, Q. A Two-Dimensional Zirconium Carbide by Selective Etching of Al_3C_3 from Nanolaminated $Zr_3Al_3C_5$. *Angew. Chem. Int. Ed.* **2016**, *55* (16), 5008–5013. https://doi.org/10.1002/anie.201510432.

(10) Zhou, J.; Zha, X.; Zhou, X.; Chen, F.; Gao, G.; Wang, S.; Shen, C.; Chen, T.; Zhi, C.; Eklund, P.; Du, S.; Xue, J.; Shi, W.; Chai, Z.; Huang, Q. Synthesis and Electrochemical Properties of Two-Dimensional Hafnium Carbide. *ACS Nano* **2017**, *11* (4), 3841–3850. https://doi.org/10.1021/acsnano.7b00030.

(11) Urbankowski, P.; Anasori, B.; Makaryan, T.; Er, D.; Kota, S.; Walsh, P. L.; Zhao, M.; Shenoy, V. B.; Barsoum, M. W.; Gogotsi, Y. Synthesis of Two-Dimensional Titanium Nitride Ti_4N_3 (MXene). *Nanoscale* **2016**, *8* (22), 11385–11391. https://doi.org/10.1039/C6NR02253G.

(12) Ghidiu, M.; Naguib, M.; Shi, C.; Mashtalir, O.; Pan, L. M.; Zhang, B.; Yang, J.; Gogotsi, Y.; Billinge, S. J. L.; Barsoum, M. W. Synthesis and Characterization of Two-Dimensional Nb_4C_3 (MXene). *Chem. Commun.* **2014**, *50* (67), 9517–9520. https://doi.org/10.1039/C4CC03366C.

(13) Tran, M. H.; Schäfer, T.; Shahraei, A.; Dürrschnabel, M.; Molina-Luna, L.; Kramm, U. I.; Birkel, C. S. Adding a New Member to the MXene Family: Synthesis, Structure, and Electrocatalytic Activity for the Hydrogen Evolution Reaction of $V_4C_3T_x$. *ACS Appl. Energy Mater.* **2018**, *1* (8), 3908–3914. https://doi.org/10.1021/acsaem.8b00652.
